# Schwann Cell Synthesized Cholesterol Orchestrates Peripheral Nerve Regeneration via Structural and IGF1‐Dependent Signaling Mechanisms

**DOI:** 10.1002/advs.202520323

**Published:** 2026-01-04

**Authors:** Shuyi Xu, Ye He, Ying Zou, Mengyao Zhao, Jiaqi Zhang, Yizhou Xu, Jiale Cai, Xiongbo Luo, Xinrui Ma, Saini Wu, Yuling Huang, Xianghai Wang, Jiasong Guo

**Affiliations:** ^1^ Department of Histology and Embryology, Guangdong Provincial Key Laboratory of Construction and Detection in Tissue Engineering, National Demonstration Center for Experimental Education, School of Basic Medical Sciences, Department of Neurosurgery, Institute of Brain Diseases, Nanfang Hospital Southern Medical University Guangzhou Guangdong China; ^2^ Key Laboratory of Mental Health of the Ministry of Education, Guangdong‐Hong Kong‐Macao Greater Bay Area Center For Brain Science and Brain‐Inspired Intelligence Guangdong Province Key Laboratory of Psychiatric Disorders Guangzhou Guangdong China; ^3^ Department of Histology and Embryology, School of Basic Medical Sciences Guangdong Pharmaceutical University Guangzhou Guangdong China

**Keywords:** FDFT1, IGF1, cholesterol, peripheral nerve injury, Schwann cell

## Abstract

Peripheral nerve myelin is the most cholesterol‐rich structure in the body, with the majority of cholesterol being synthesized by Schwann Cells (SCs). Following peripheral nerve injury, myelin disintegration leads to substantial cholesterol release and accumulation, which has been suggested to aggravate neuroinflammation and hinder nerve repair in the central nervous system. However, whether cholesterol synthesis by SCs is detrimental or beneficial for peripheral nerve regeneration remains a critical and unresolved question. Present findings reveal that FDFT1, a key cholesterol biosynthesis enzyme, is downregulated within two weeks post‐injury but significantly upregulated thereafter. Conditional knockout (cKO) of *Fdft1* in SCs markedly impaired structural and functional recovery in mice after the sciatic nerve crush injury. Mechanistically, SCs’ *Fdft1* deficiency not only disrupts the cholesterol supply for remyelination but also suppresses the secretion of insulin‐like growth factor 1 (IGF1). This impairment of IGF1 signaling further attenuates the axonal regeneration by paracrine mechanisms and disrupts the remyelination via a novel IGF1R/Rap1/PI3K/AKT axis within SCs. In conclusion, this work demonstrates that SC synthesized cholesterol plays dual roles in orchestrating nerve regeneration: it serves as an essential structural component of myelin and also works as a regulator of IGF1 expression to enhance axonal regeneration and remyelination.

## Graphical abstract

This study reveals that Schwann cell FDFT1‐mediated cholesterol synthesis is essential for peripheral nerve regeneration via dual roles: as a structural component for myelin and as a metabolic signal that upregulates IGF1. IGF1 promotes axonal growth through paracrine action and enhances Schwann cell differentiation/ myelination via an intrinsic IGF1R/Rap1/PI3K/AKT axis.

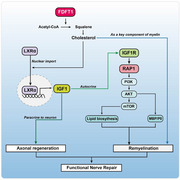

## 1 Introduction

Peripheral nerves are among the most cholesterol‐enriched tissues in the human body [1, 2]. Cholesterol serves as a fundamental structural component of myelin sheaths [3, 4], which are essential for insulating axons and ensuring efficient saltatory conduction of nerve impulses. The cholesterol content in peripheral myelin is highly dependent on *de novo* synthesis by SCs [5, 6, 7], the principal glial cells of the peripheral nervous system [8, 9]. In contrast to nerve development, where the role of SCs in cholesterol production for myelination is clear, the situation during nerve injury and repair is much more complex. First, a substantial amount of cholesterol is released from disintegrated myelin debris during Wallerian degeneration following nerve injury [10, 11]. Cholesterol cannot be catabolized by mammalian cells and cholesterol efflux in nervous tissue is challenging [5, 12, 13]. In injured spinal cord, cholesterol accumulation is thought to contribute to neuroinflammation, thereby impeding effective nerve repair [14, 15, 16]. This leads to the suggestion that inhibiting cholesterol biosynthesis (e.g., with statins) could promote axonal regeneration after spinal cord injury [17, 18, 19]. However, the situation in the peripheral nerve injury is distinct due to its efficient debris clearance [20, 21, 22]. Moreover, successful nerve regeneration and remyelination have an undeniable demand for cholesterol [3, 23, 24, 25]. This raises a fundamental question: Is cholesterol biosynthesis by SCs detrimental or beneficial for peripheral nerve repair? This question remains unresolved.

Previous studies have demonstrated that the specific knockout of farnesyl‐diphosphate farnesyltransferase 1 (*Fdft1*), a key enzyme in the cholesterol biosynthesis pathway, in SCs leads to hypomyelination in peripheral nerves [6]. However, how FDFT1 expression and function are regulated in SCs after peripheral nerve injury remains unclear. Intriguingly, beyond its canonical role in cholesterol synthesis, emerging evidence from cancer biology suggests that FDFT1 can influence cell proliferation and survival by modulating the PI3K/AKT signaling pathway [26, 27, 28, 29]. The PI3K/AKT axis is also well known for its pivotal role in SC biology. For instance, activation of the PI3K/AKT pathway promotes SCs migration, proliferation, and remyelination [30, 31, 32, 33]. However, the mechanistic link between FDFT1 and PI3K/AKT signaling remains entirely unexplored.

Here, we find that FDFT1 exhibits a biphasic expression pattern after sciatic nerve crush, down‐regulated early but significantly up‐regulated during the regenerative phase. Using inducible SC‐specific Fdft1 knockout mice, we demonstrate that FDFT1‐mediated cholesterol biosynthesis in SCs is essential for axonal regeneration, remyelination and functional recovery. Mechanistically, FDFT1‐derived cholesterol not only supplies structural component for myelination but also activates a signaling pathway via LXRα to up‐regulate IGF1 expression in SCs. Secreted IGF1 then promotes axonal regeneration through paracrine mechanisms while simultaneously acting on SCs via the IGF1R/Rap1/PI3K/AKT axis to drive their differentiation and remyelination. This study reveals that SC‐intrinsic cholesterol synthesis plays an indispensable dual role in nerve repair and identifies FDFT1 as a novel therapeutic target for nerve regeneration.

## 2 Result

### 2.1 The Expression Pattern of Cholesterol Synthesis‐related Genes in SCs Following PNI

**FIGURE 1:**
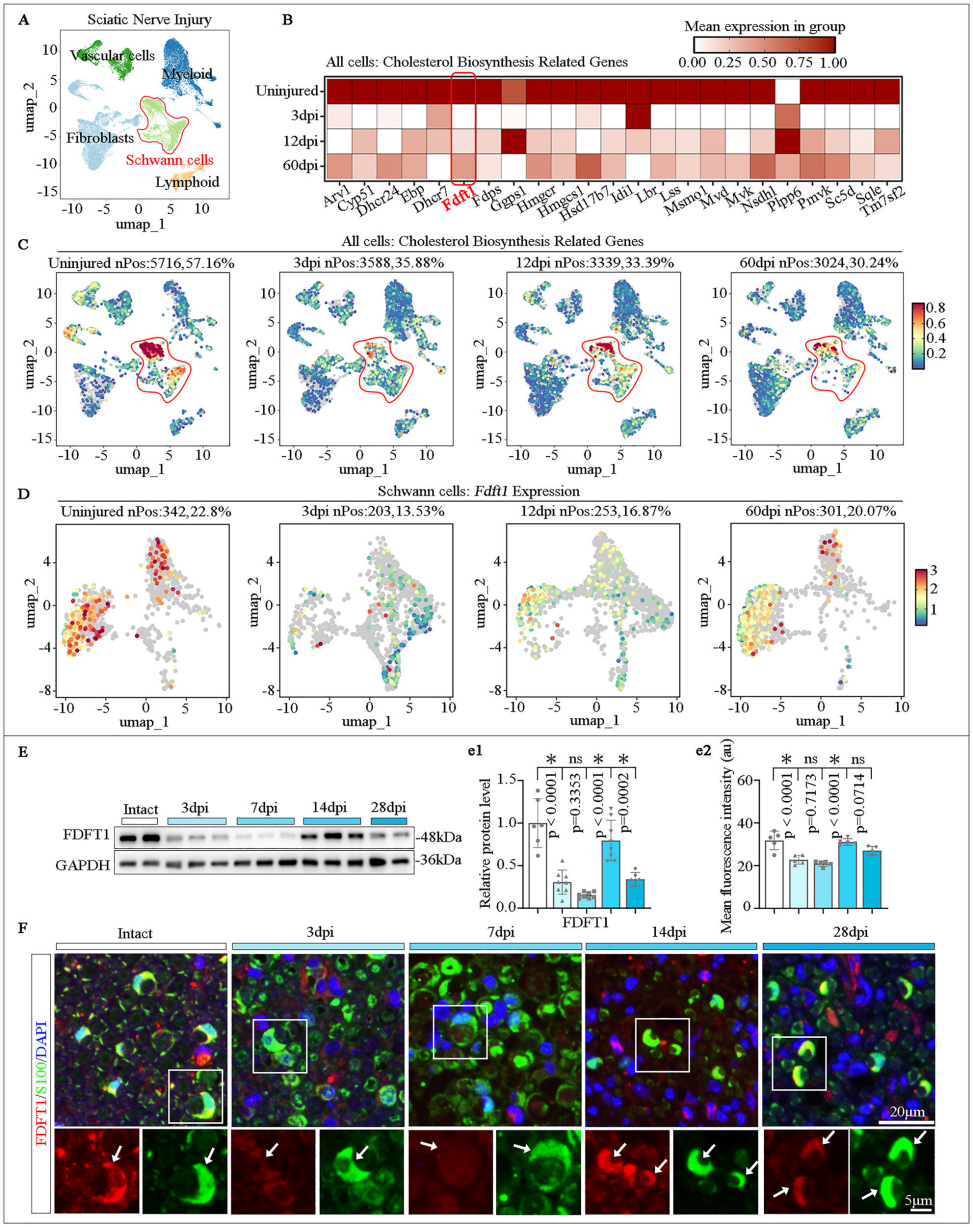
The expression pattern of cholesterol synthesis‐related genes in SCs following PNI. (A) Subpopulation analysis based on reprocessed single‐cell RNA‐sequencing data (GSE216665). (B,C) Altered expression and cellular localization patterns of key enzymes in the cholesterol synthesis pathways at 3, 12 and 60 dpi. (D) The expression dynamics of FDFT1 in SCs at 3, 12 and 60 dpi. (E) Western blot analysis of FDFT1 protein levels in the sciatic nerve at 3, 7, 14 and 28 dpi (*n* = 6–8). (F) Double immunofluorescence staining and quantification (e1) for FDFT1 (red) and SCs marker S100 (green) in transverse sections of the injured nerves at 3, 7, 14 and 28 dpi. Scale bar = 20 µm, zoom in, 5 µm, (*n* = 5). Data are presented as mean ± SD. Statistical analysis was performed using one‐way ANOVA for multiple comparisons, “ns” indicating no significance, *p < 0.05.

Bioinformatic analysis based on single‐cell RNA sequencing data from the GEO database (GSE216665 [34]) indicated that cholesterol synthesis‐related genes in SCs exhibited a trend of initial decline and late up in the rat sciatic nerve chronic constriction injury model (Figure 1A–D). Similarly, in our mouse sciatic nerve crush injury model, Western blot analysis confirmed the downregulation of FDFT1 during the 3–7 days post injury (dpi), followed by upregulation at later stages (14‐28 dpi). Double immunofluorescence (IF) staining of FDFT1 and S100 revealed that FDFT1 is predominantly expressed in SCs, with its abundance patterns aligning with the results from Western blot analysis and database findings (Figure 1E,F). Furthermore, Filipin III staining demonstrated an increase in free cholesterol levels during the early stage of Wallerian degeneration, and a subsequent decrease in the later stage of nerve injury (Figure S1), which was contrary to the expression pattern of FDFT1. These findings suggest that FDFT1 is primarily expressed in SCs, and it is negatively regulated by the accumulation of free cholesterol during Wallerian degeneration. This phenomenon was also verified by an in vitro experiment, which indicates that squalene or cholesterol treatment can significantly alleviate FDFT1 expression in the cultured Schwann cells (Figure S2). However, the potential role of FDFT1 upregulation in the later stage after peripheral nerve injury warrants further investigation.

### 2.2 Identification of the FDFT1‐Specific Depletion in SCs

**FIGURE 2:**
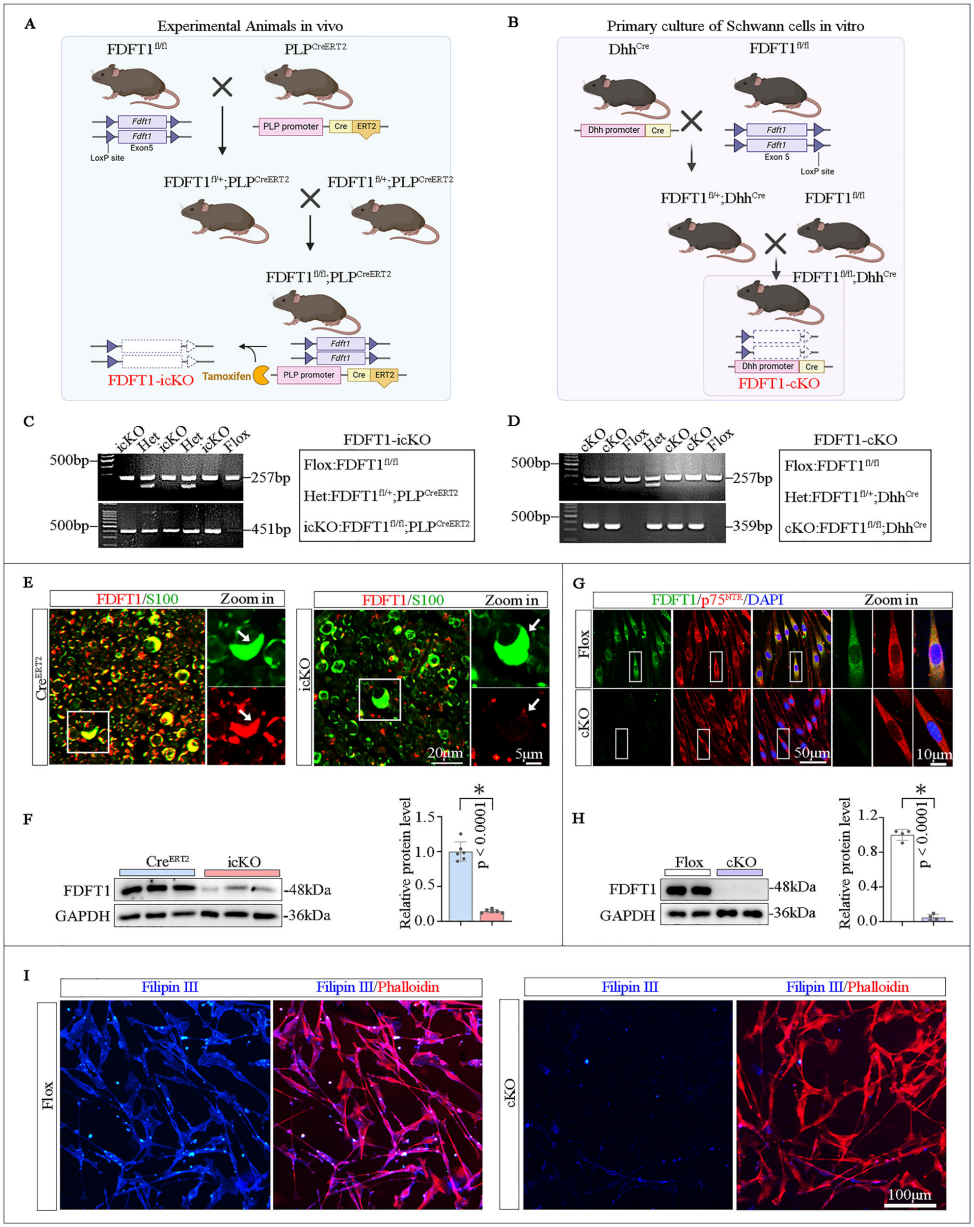
Generation and identification of SC‐specific FDFT1 knockout mice. (A,B) Breeding strategies of the two lines of conditional knockout mice. (C,D) Representative genotyping images of icKO (*Fdft1* ^flox/flox^; *Plp* ^CreERT2^) and cKO (*Fdft1* ^flox/flox^; *Dhh* ^Cre^) mice. (E,F) FDFT1 deletion efficiency in adult icKO mice, as shown by double immunofluorescence staining for FDFT1 (red) and S100 (green), and western blotting analysis in (F) (*n* = 6). Scale bar = 20 µm, zoom in, 5 µm. (G,H) Immunofluorescence and Western blot analyses (H) confirm the FDFT1‐knockout efficiency in primary SCs isolated from cKO neonatal mice. Scale bar = 50 µm, zoom in, 10 µm (*n* = 4). I) Filipin III staining (blue) and Phalloidin staining (red) visualize the cholesterol levels in the primary cultured SCs. Scale bar = 100 µm. All data are presented as mean ± SD. Two‐tailed Student's *t*‐test, “ns” indicating no significance, *p < 0.05.

To investigate the role of FDFT1 in the repair of peripheral nerve injuries, we developed two types of mice with conditional knockout of FDFT1 in SCs. The *Fdft1* ^flox/flox^; *Dhh* ^Cre^ (cKO) combination represents an embryonic stage conditional knockout mouse model, primarily used for in vitro culture of SCs, while *Fdft1* ^flox/flox^ (Flox) serves as the littermate control. The *Fdft1* ^flox/flox^; *Plp* ^CreERT2^ (icKO) combination employs tamoxifen‐induced conditional knockout, designed for peripheral nerve injury models, with *Plp* ^CreERT2^ (Cre^ERT2^) serving as the littermate control (Figure 2A,B). This approach ensures that pre‐injury neural development is consistent with that of the Cre^ERT2^ mice, thereby minimizing experimental variability. After the transgenic mouse lines were validated via genotyping (Figure 2C,D), immunofluorescent staining and Western blotting analyses were executed to confirm the successful knockdown of FDFT1 in SCs in the nerve of icKO mice (Figure 2E,F), and the primary SCs isolated from the cKO mice (Figure 2G,H). Furthermore, Filipin III staining demonstrated that the cholesterol level is significantly decreased in the FDFT1‐deficient SCs (Figure 2I). These findings confirm the successful establishment of the FDFT1‐specific knockout models for subsequent experiments.

### 2.3 FDFT1 Deficiency in SCs Inhibits Axonal Regeneration and Remyelination Following Sciatic Nerve Crush Injury

**FIGURE 3:**
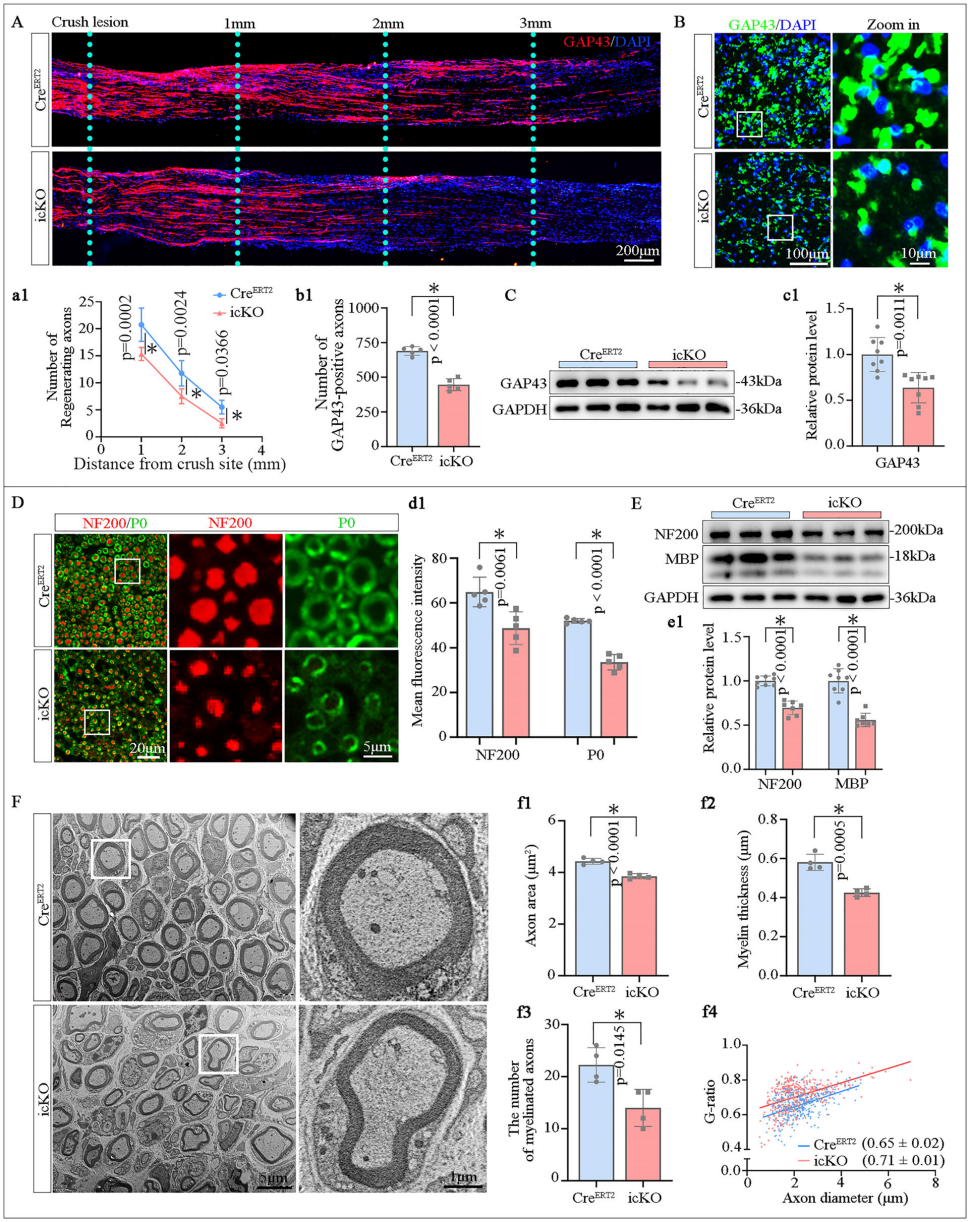
FDFT1 deficiency in SCs impairs axonal regeneration and remyelination following sciatic nerve crush injury. (A) Immunostaining and quantifications (a1) show the GAP43^+^ regenerating axons in longitudinal sections of the injured nerves at 3 dpi. Scale bars = 200 µm, (*n* = 5). (B) Immunostaining displays GAP43^+^ axons in cross sections 3 mm distal to the injury site at 3 dpi, quantification shown in (b1). Scale bar = 100 µm, zoom in, 10 µm, (*n* = 5). (C) Western blot analysis and quantification (c1) of GAP43 protein levels in the distal nerve trunks at 3 dpi (*n* = 8). (D) Immunostaining and fluorescence intensity quantification (d1) of P0 (red) and NF200 (green) on the transverse sections of the nerve at 3 mm distal to the lesion site at 28 dpi. Scale bar = 20 µm, zoom in, 5 µm, (*n* = 5). (E) Western blotting and protein levels (e1) of MBP and NF200 in the distal trunk of the injured nerve at 28 dpi (*n* = 7–8). (F) Transmission electron microscopy (TEM) analysis of the regenerated nerve 3 mm distal to the lesion at 28 dpi, showing axon area (f1), myelin thickness(f2), the number of myelinated axons (f3) and G‐ratio (f4), (*n* = 4). Data are presented as mean ± SD. Two‐tailed Student's *t*‐test, “ns” indicating no significance, *p < 0.05.

At 3 dpi, immunostaining on longitudinal sections of the injured nerves revealed that the length of GAP43^+^ regenerating axons extending from the injury site was significantly shorter and their number was fewer in the icKO mice compared to their Cre^ERT2^ littermates (Figure 3A). Meanwhile, cross‐sectional immunostaining at 3 mm distal to the injury site showed that the number of GAP43^+^ axons in the icKO group was smaller than that in the Cre^ERT2^ group (Figure 3B). Additionally, Western blot analysis confirmed a decreased protein level of GAP43 in the distal nerves of the icKO group (Figure 3C). These findings indicate that FDFT1 deficiency in SCs impedes axonal regeneration during the early stages of nerve injury.

At 28 dpi, both immunostaining and Western blot analyses showed significantly lower expression levels of P0, MBP and NF200 in the icKO group (Figure 3D,E). Quantitative analysis of the transmission electron microscopy (TEM) images revealed that the axonal area size and the thickness of myelin sheath were decreased in the icKO mice, and the G‐ratio of nerve fibers was significantly increased in the icKO group compared to the Cre^ERT2^ group (Figure 3F). Furthermore, immunofluorescent staining indicated that the density of F4/80^+^ macrophages in the crush‐injured nerve at 3 dpi or in the transection‐injured nerve at 5 dpi was similar between the Cre^ERT2^ group and icKO groups(Figure S3A,B). Meanwhile, SC‐FDFT1 deficiency did not alter the levels of markers of M1 (iNOS) and M2 (Arg1) polarization of macrophages in the injured nerve (Figure S3C). Collectively, these results demonstrate that FDFT1 deficiency in SCs attenuates axonal regeneration and remyelination in the injured nerve independent of neuroinflammation.

### 2.4 FDFT1 Deficiency in SCs Attenuates Functional Nerve Repair

**FIGURE 4:**
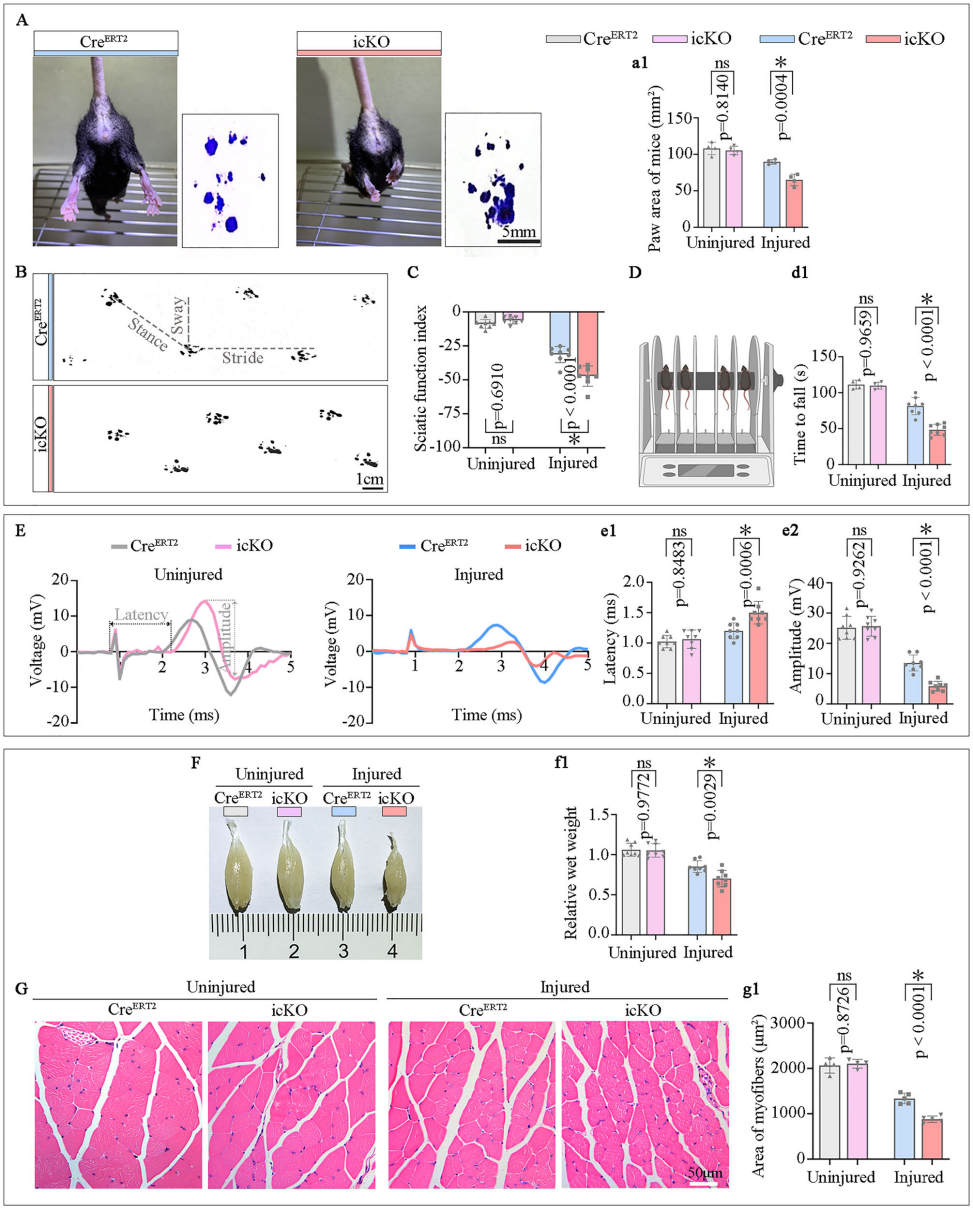
SC‐specific FDFT1 knockout hinders morphological and functional recovery after sciatic nerve injury at 28 dpi. (A) The hindlimb extension and paw area (a1) during suspension. Scale bar = 5 mm, (*n* = 4). (B,C) Representative footprints image, and the SFI scores shown in (C). Scale bar = 1 cm, (*n* = 8). (D) Motor coordination assessed by rota‐rod test at accelerating speed from 5 rpm to 40 rpm over 120 s. Quantification of time to fall (d1), (*n* = 4–8). (E) Respective CMAP images and the quantification of latency (e1) and amplitude (e2), (*n* = 8). (F) Gross morphology and wet weight ratio (f1) of the gastrocnemius muscle, (*n* = 8). (G) HE staining and fiber cross‐sectional area analyses (g1) of the gastrocnemius muscle. Scale bar = 100 µm, (*n* = 4–5). Data are presented as mean ± SD. Two‐way ANOVA, “ns” indicating no significance, *p < 0.05.

To evaluate the functional repair after the nerve injury, we executed assessments of motor behavior, nerve conduction, and amyotrophy of the target muscle. The hind limb extension during the mice was suspended (Figure 4A), as well as the sciatic nerve function index (SFI) score derived from gait analysis (Figure 4B,C) were reduced in the icKO group. Meanwhile, motor function assessments including hind limb stride length analysis, the rota‐rod test and the amplitude of CMAP were also impaired in the icKO group compared to the Cre^ERT2^ group (Figure 4D,E; Figure S4). Furthermore, the gross size, wet weight, and myofiber size ratio of the gastrocnemius muscles (Figure 4F,G), as well as the reinnervated neuromuscular junction (Figure S5) on the injured side were diminished in the icKO group. Notably, the parameters of behavior, CMAP, and gastrocnemius muscle parameters have no differences were observed in the uninjured contralateral limbs of the Cre^ERT2^ and icKO groups (Figure 4A–G; Figure S4). Moreover, no significant differences were observed in the axonal or myelin sheath structures of either the sciatic nerve or optic nerves (a central nervous system tract) between Cre^ERT2^ mice and icKO mice (Figures S6 and S7). These findings suggest that FDFT1 depletion in SCs impairs both morphological and functional recovery after nerve injury, and these phenotypes are due to impaired regeneration rather than pre‐existing developmental defects.

### 2.5 The Supplementation of Squalene Reverses the Effects of *Fdft1* icKO in the Regeneration After Nerve Injury

**FIGURE 5:**
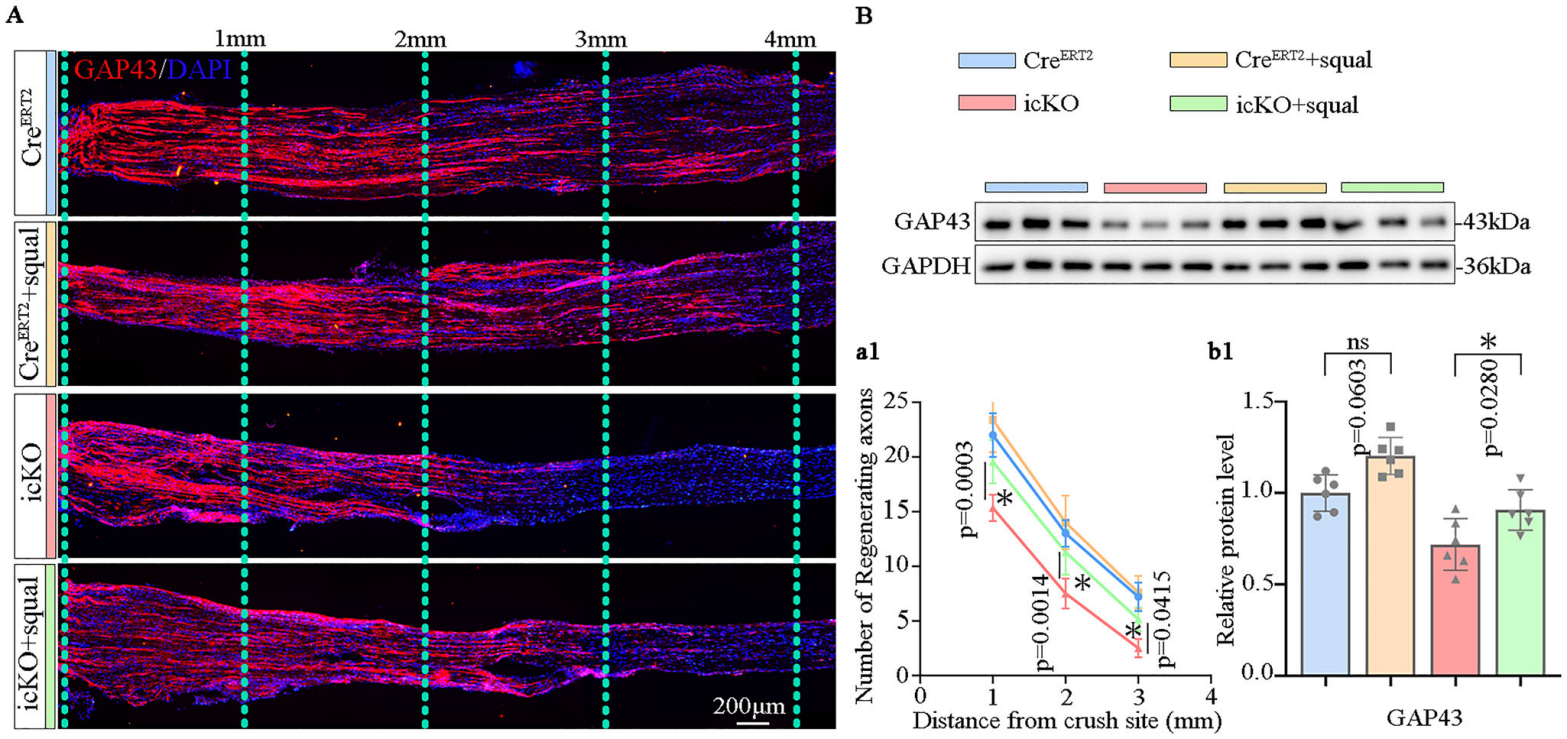
Squalene administration rescues the SC‐FDFT1 deficiency delayed axonal regeneration at 3dpi. (A) Immunofluorescent staining and quantifications (a1) of GAP43^+^ regenerating axons in the longitudinal sections of crush‐injured nerves at 3 dpi. Scale bar = 200 µm, (*n* = 4). (B) Western blotting and analysis of GAP43 protein levels (b1) in the distal trunk of the injured nerves at 3 dpi (*n* = 6). Data are presented as mean ± SD. Two‐way ANOVA, “ns” indicating no significance, *p < 0.05.

**FIGURE 6:**
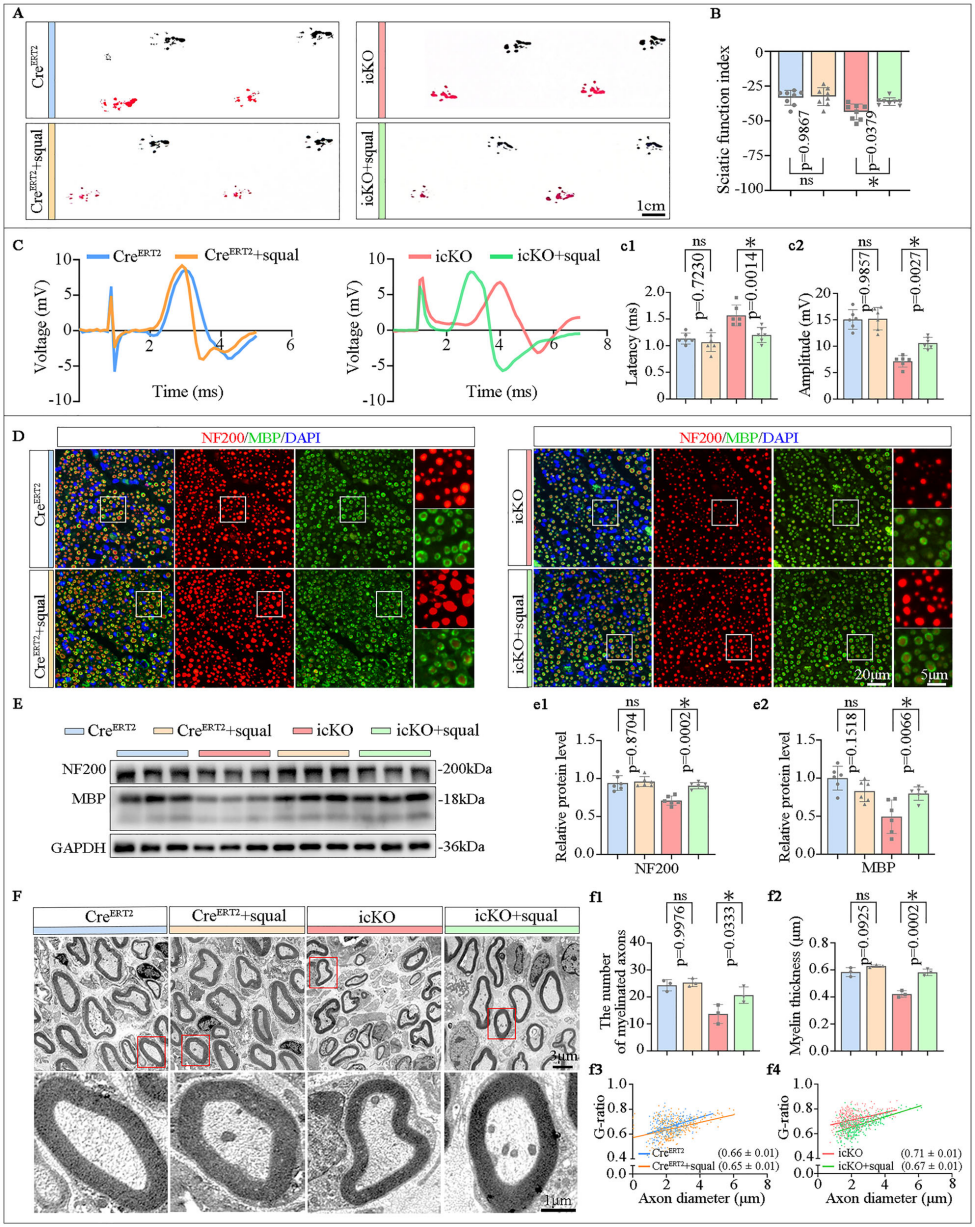
Squalene administration rescues the SC‐FDFT1 deficiency impaired functional regeneration at 28 dpi. (A) Representative footprints and the SFI scores (a1) at 28 dpi, (*n* = 6). (B) Respective CMAP images and quantified latency (b1) and amplitudes (b2), (*n* = 6). (C) Immunostaining for NF200 (red) and MBP (green) on the transverse sections of the nerve at 3 mm distal to the lesion site at 28 dpi. Scale bar = 20 µm, zoom in, 5 µm. (D) Western blotting analysis of MBP and NF200 protein levels. Quantifications shown in (d1,d2), (*n* = 6). (E) TEM analysis shows the number of myelinated axons (e1), myelin thickness (e2) and G‐ratio (e3, e4) at 3 mm from the injury site at 28 dpi. Scale bar = 3 µm, zoom in, 1 µm. (*n* = 3). Data are presented as mean ± SD. Two‐way ANOVA, “ns” indicating no significance, *p < 0.05.

As a key enzyme in the cholesterol synthesis pathway, FDFT1 catalyzes the formation of squalene, ultimately leading to cholesterol production. To investigate whether FDFT1 regulates neural regeneration by influencing squalene synthesis, squalene was administered via gavage to icKO and Cre ^ERT2^ mice (Cre^ERT2^ + squal, icKO + squal) for 3 days or 28 days post‐injury. The icKO and Cre^ERT2^ control groups received an equal volume of corn oil instead. At 3 dpi, the immunostaining revealed that the length of GAP43^+^ axons extending from the injured site was significantly longer in the icKO + squal mice compared to the icKO mice (Figure 5A). Besides, Western blotting analysis demonstrated that the protein level of GAP43 in distal nerves was also elevated in the icKO + squal group (Figure 5B). In contrast, the squalene administration did not statistically affect the length of GAP43^+^ axons and the GAP43 protein level in the Cre^ERT2^ mice. At 28 dpi, the motor function assessments including hind limb stride length analysis, SFI index, the rota‐rod test, and the amplitude of CMAP (Figure 6A–C; Figure S8A–D) showed improvements in the icKO + squal group compared to the icKO control group. FluoroMyelin staining of transverse sections revealed an increase in myelin content in the regenerated nerves located 3 mm distal from the injury site in the icKO + squal group, as opposed to the icKO group (Figure S8E). Concurrently, both immunostaining and Western blot analyses showed that the expression levels of NF200 and MBP were significantly elevated in the icKO + squal group (Figure 6C,D; Figure S8F). Additionally, TEM assessments indicated a significantly reduced G‐ratio in the icKO + squal group relative to the icKO group, accompanied by increased thickness of regenerated myelin sheaths (Figure 6E). Furthermore, the gross size, wet weight, and myofiber size ratio of the injured gastrocnemius muscles displayed marked enlargement compared to the icKO group (Figure S8G,H). These results suggest that FDFT1 icKO affects the nerve repair through its role in squalene synthesis.

### 2.6 FDFT1 Deletion Impairs SCs Differentiation, Which Can be Rescued by the Supplementation of Squalene

**FIGURE 7:**
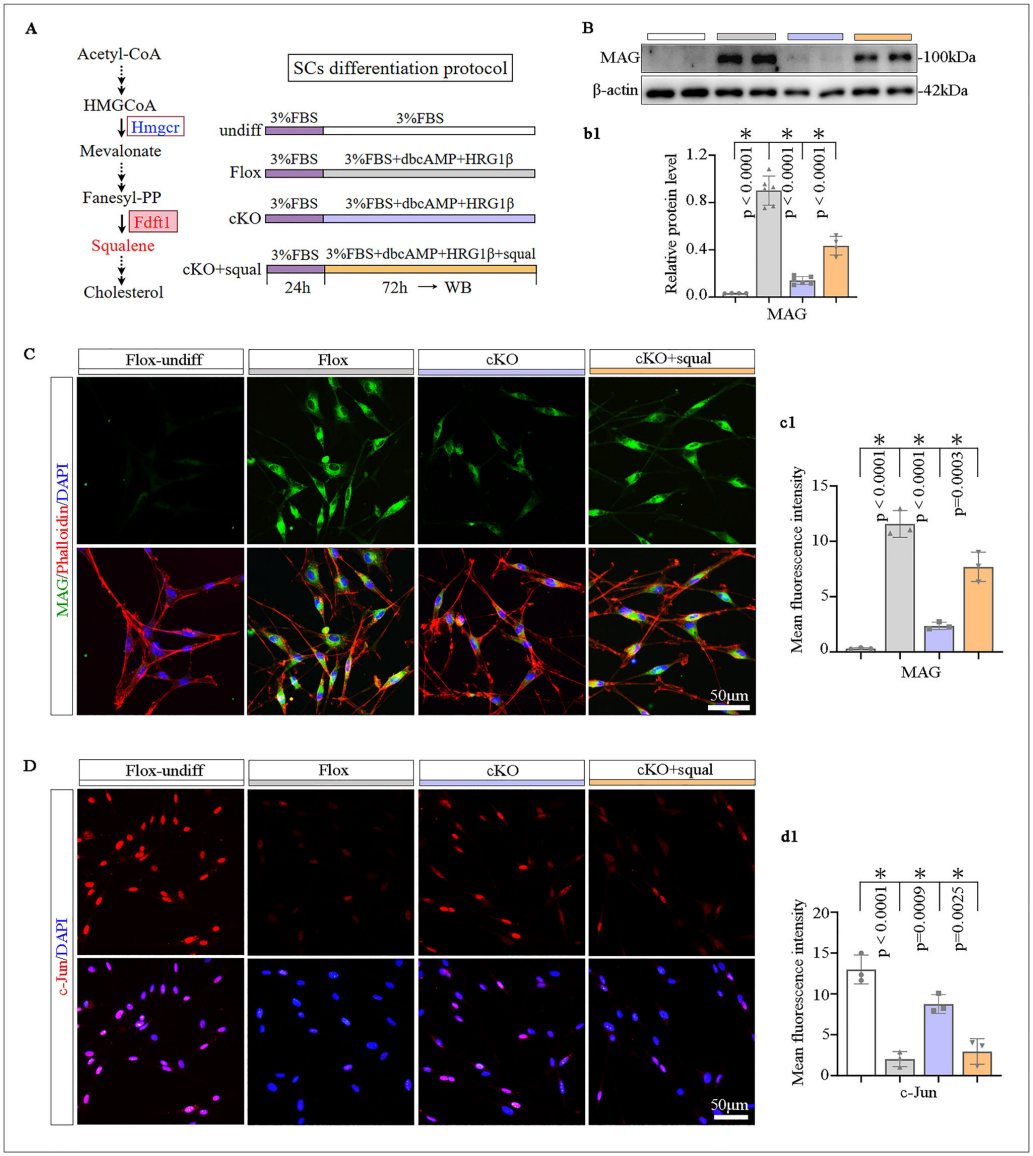
FDFT1 deletion impaired SCs differentiation, is rescued by the squalene administration. (A) Schematic of the experimental design for inducing SCs differentiation with squalene or cholesterol treatments. (B) Western blot analysis of MAG protein levels, quantification shown in (b1), (*n* = 4–6). (C) Immunofluorescent staining and quantification (c1) for MAG (green) and Phalloidin staining (red). Scale bar = 50 µm, (*n* = 4). (D) Immunofluorescent staining and quantification (d1) for c‐Jun (red). Scale bar = 50 µm, (*n* = 4). Data are presented as mean ± SD. Two‐way ANOVA, “ns” indicating no significance, *p < 0.05.

To explore the role of FDFT1 in SCs differentiation, which is the prerequisite for initiating myelination, primary SCs were isolated from cKO and Flox neonatal mice and were treated with dbcAMP and HRG1β to induce the differentiation, meanwhile, squalene was supplemented into the cKO group to evaluate the rescue effect (Figure 7A). WB and IF revealed that the MAG (a marker of mature SCs) upregulation in the differentiation‐induced SCs was dramatically weaker in the cKO group compared to the Flox group, whereas the squalene supplement could efficiently elevate MAG levels in the cKO SCs (Figure 7B,C). Meanwhile, c‐Jun (a marker of immature SCs) up‐regulation in the cKO SCs was also reversed by squalene administration (Figure 7D). We also checked O1 (another marker of mature SCs) and p75^NTR^ (another marker of immature SCs) with immunostaining. The results showed that their expression patterns were similar with MAG and c‐Jun in the Flox SCs and cKO SCs, and they were also markedly rescued by the supplementation of squalene or cholesterol (Figure S9). The optimal concentrations of squalene (200 µM) and cholesterol (1 µg mL^−1^) were selected in prior experiments (Figure S10A–D). The Filipin III staining also revealed a significant decrease in cholesterol levels in the cKO SCs, while squalene or cholesterol added to the culture medium significantly enhanced the level of cholesterol in the cKO cells (Figure S10E). These results demonstrate that FDFT1 deletion impairs SCs differentiation, which is attributed to the impaired squalene or cholesterol synthesis.

### 2.7 FDFT1 Regulates PI3K/Akt Pathway Through a Novel LXRα/IGF1/IGF1R/Rap1 Axis

**FIGURE 8:**
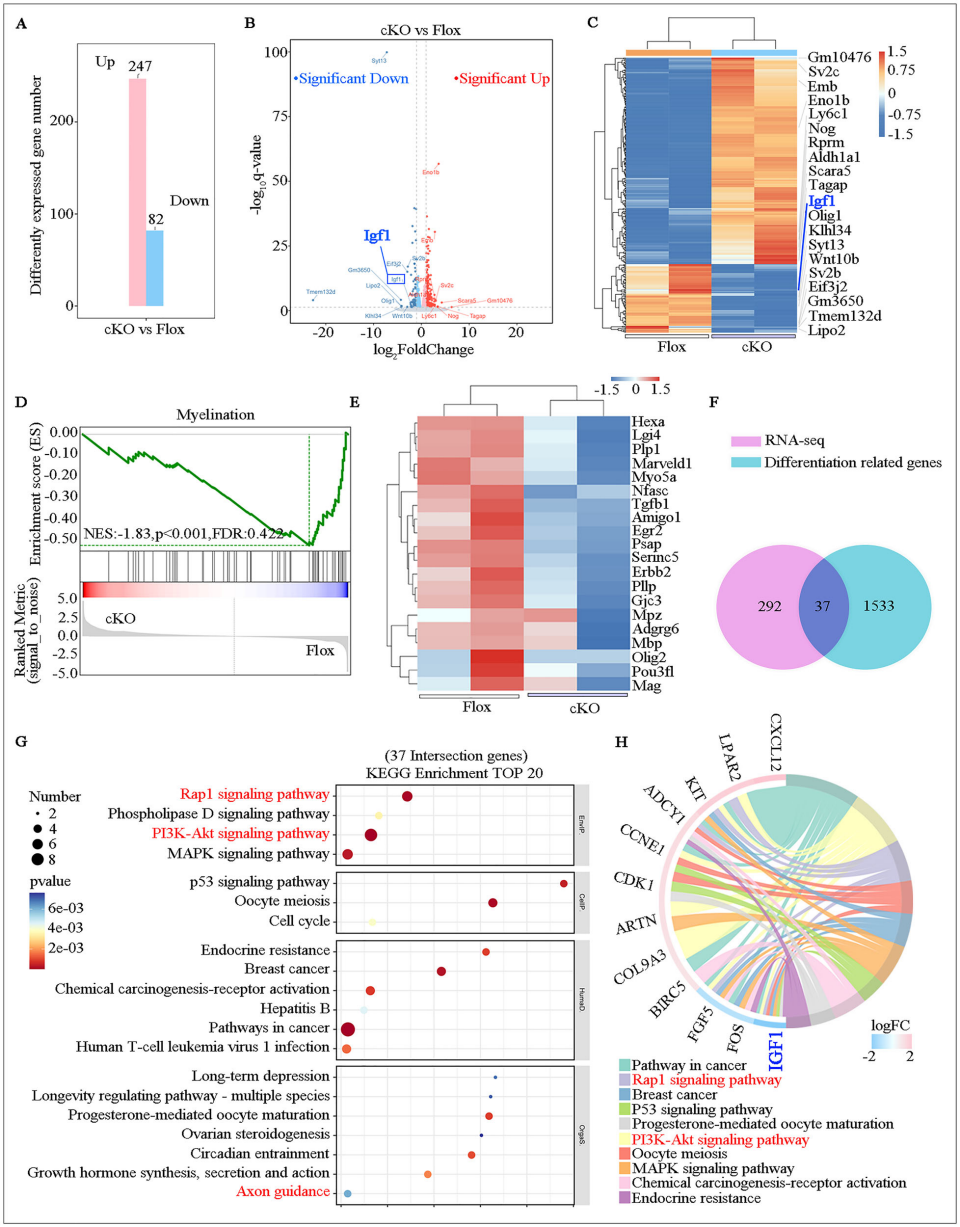
RNA sequencing and analysis of the primary SCs isolated from Flox and cKO mice. (A) Significantly upregulated and downregulated genes with *p* < 0.05 in sequencing results. (B) Volcano plot of differentially expressed genes (DEGs), Red and blue dots indicate significantly up‐ and down‐regulated genes (*p* < 0.05), respectively. (C) Heatmap of DEGs across samples. (D,E) GSEA and corresponding heatmaps show changes in myelination‐related genes. (F) Venn diagram shows the overlap between DEGs and 1570 differentiation‐related genes. (G,H) KEGG pathway analysis of the 37 overlapping genes.

To elucidate whether FDFT1 may regulate SCs biofunctions through modulating a signaling pathway rather than merely providing cholesterol as a key component of myelin, we conducted transcriptomic profiling using RNA sequencing (RNA‐seq) with Flox and cKO primary SCs. The results revealed that 247 genes were significantly up‐regulated, while 82 genes showed a significant reduction in the cKO group compared to the Flox group (fold change > 1.25; *p* < 0.05) (Figure 8A–C). Gene set enrichment analysis (GSEA) demonstrated that the myelination process was adversely affected in the cKO group, with a heatmap revealing that myelination‐related genes, including MBP and EGR2 (Krox20) were down‐regulated (Figure 8D,E). The Venn diagram illustrated the identification of 37 intersecting genes between the RNA‐seq data and 1570 differentiation‐related genes collected from GeneCards (Figure 8F). Further KEGG analysis identified the top 20 signaling pathways, including the Rap1 pathway, PI3K/Akt pathway, and axon guidance (Figure 8G,H). Notably, the level of IGF1 expression was significantly reduced (Figure 8H).

**FIGURE 9:**
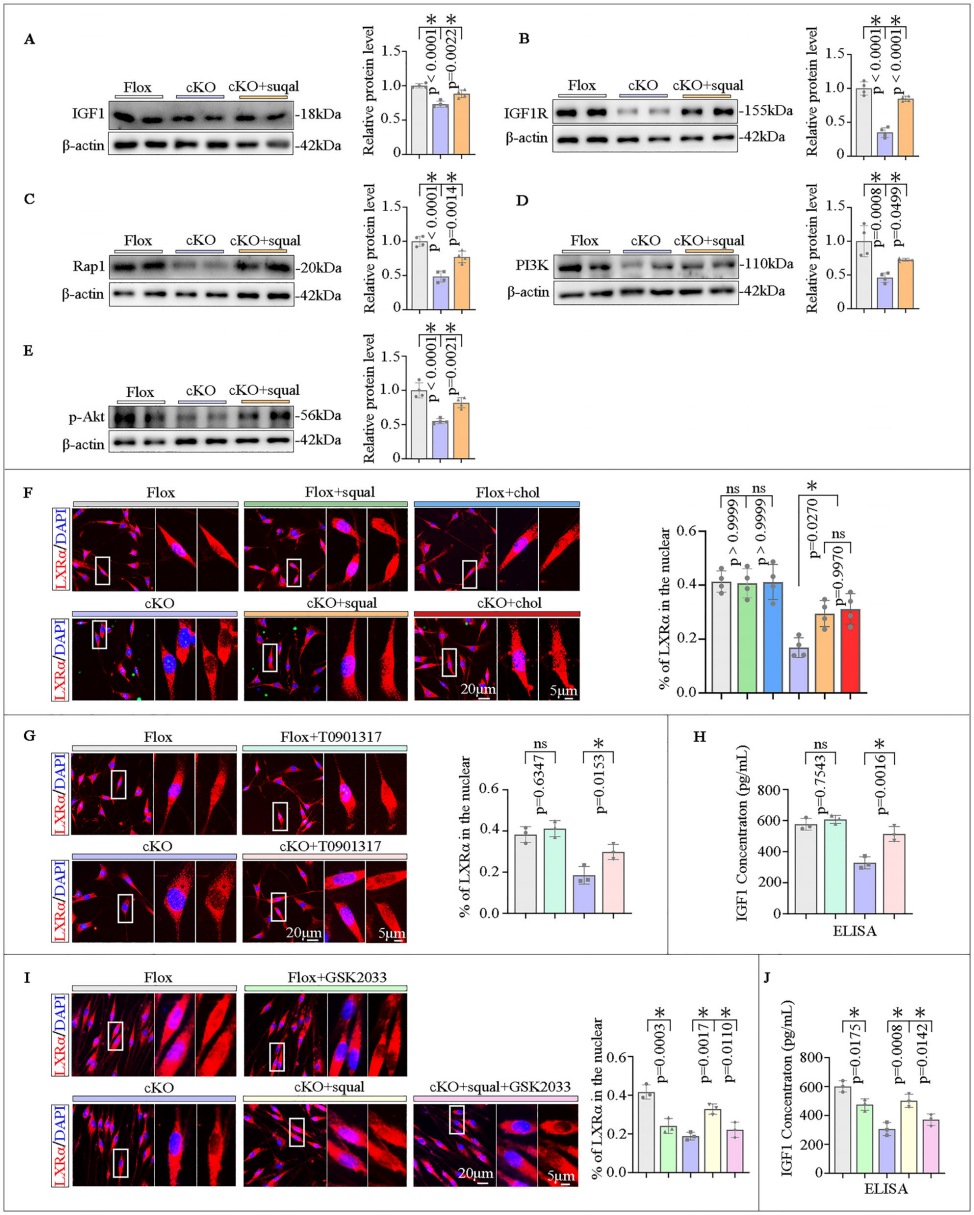
FDFT1 deletion in SCs impedes axon regeneration via squalene/cholesterol /LXRα/IGF1 signaling pathway. (A–E) Western blot analysis of the protein levels of IGF1, IGF1R, Rap1, PI3K and p‐Akt in the primary SCs of Flox, cKO and cKO+Squalene groups. (F) LXRα nuclear localization in primary cultured Flox and cKO SCs, and after squalene or cholesterol treatments, is illustrated by immunofluorescence. Scale bar = 20 µm, zoom in, 5 µm, (*n* = 4). (G) Effect of LXRα agonist T0901317 on LXRα nuclear localization in cKO SCs is assessed by immunofluorescence. Scale bar = 20 µm, zoom in, 5 µm, (*n* = 3). (H) IGF1 levels in the cultured supernatants of primary SCs after treating with T0901317 are measured by ELISA (*n* = 3). (I) Analyze the level of LXRα nuclear import after LXR antagonist GSK2033 treatment, and the reversed effect of squalene supplementation in treated cKO SCs. Scale bar = 20 µm, zoom in, 5 µm, (*n* = 3). (J) IGF1 levels in the cultured supernatants of primary SCs after treatment from (I) are measured by ELISA (*n* = 3). Data are presented as mean ± SD. Two‐way ANOVA, “ns” indicating no significance, *p < 0.05.

Consistent with the RNA‐seq findings, Western blotting analysis revealed that the expression levels of IGF1 and IGF1R (the receptor for IGF1) were lower in the cKO SCs compared to the Flox SCs (Figure S11A,B). Additionally, the expression levels of Rap1 and PI3K were also down‐regulated (Figure S11C,D), along with a marked decrease in the levels of p‐Akt and p‐mTOR in the cKO group (Figure S11E,F). Meanwhile, squalene treatment resulted in a recovery of these protein levels in the cKO SCs (Figure 9A–E).

As cholesterol's main sensor, nuclear receptor liver X receptor α (LXRα) is an important transcription factor regulating the expression of target genes [35, 36]. Immunostaining analysis demonstrated that the nuclear level of LXRα was markedly decreased in the cKO SCs compared to the Flox SCs. The treatments of squalene or cholesterol did not affect the nuclear import in the Flox SCs but could significantly increase the nuclear import in the cKO SCs (Figure 9F). Importantly, using a LXR activator (T0901317) to treat the cKO SCs could efficiently rescue the decreased nuclear level of LXRα and increase the IGF1 level in the culture medium (Figure 9G,H). Furthermore, the LXR antagonist GSK2033 treatment significantly reduced the nuclear level of LXRα in the Flox SCs and reversed the effect of squalene to promote LXRα nuclear import in cKO SCs (Figure 9I). Concomitantly, IGF1 secretion levels in the GSK2033 treated cells were decreased accordingly (Figure 9J).

### 2.8 SCs FDFT1 Plays a Role in Promoting Neurite Growth via Squalene/Cholesterol‐mediated IGF1 Paracrine

**FIGURE 10:**
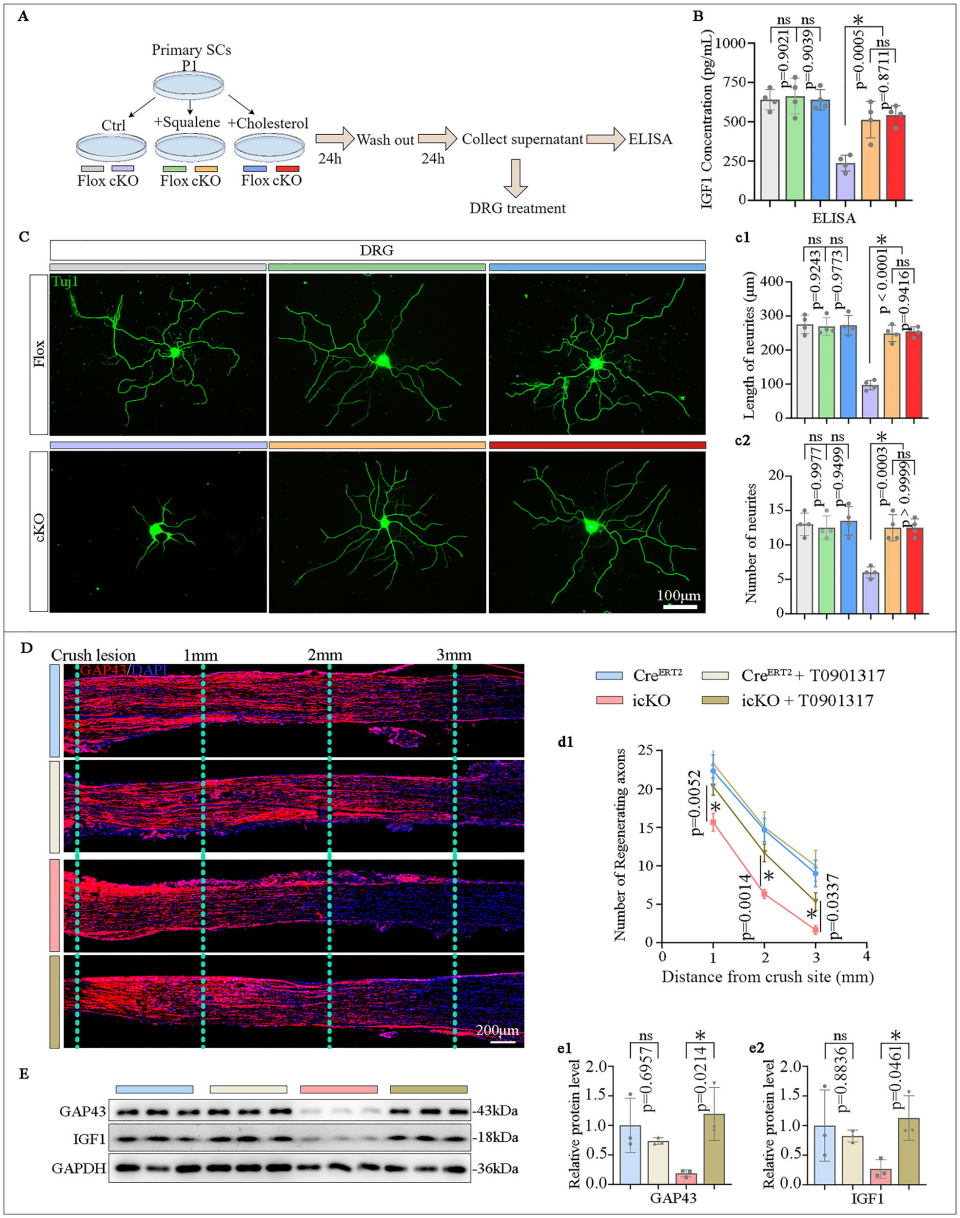
Squalene and cholesterol treatments promote neurite growth via IGF1 secretion from SCs. (A) Schematic of the experimental designs of collecting conditional medium (CM) of squalene or cholesterol‐treated SCs for ELISA assay and co‐culture with DRG neurons. (B) IGF1 secretion in the SCs CM measured by ELISA (*n* = 4). (C) The neurite growth of DRG neurons is assessed by immunofluorescent staining and quantification (e1, e2). Scale bar = 100 µm, (*n* = 4). (D) Immunostaining and quantification (d1) show the GAP43^+^ regenerating axons in longitudinal sections of the injured nerves at 3 dpi while T0901317 was supplemented in vivo. Scale bars = 200 µm, (*n* = 3). (E) Western blot analysis and quantification (e1, e2) of GAP43 protein levels in the distal nerve trunks at 3 dpi, while T0901317 was supplemented in vivo (*n* = 3). Data are presented as mean ± SD. Two‐way ANOVA, “ns” indicating no significance, *p < 0.05.

To further verify whether the SCs FDFT1 plays a role in axonal regeneration through the squalene/cholesterol‐mediated IGF1 paracrine mechanism, both Flox and cKO SCs were treated with either squalene or cholesterol, alongside untreated controls, following the schematic design (Figure 10A). ELISA analysis revealed that the concentrations of IGF1 in the SCs culture medium (CM) were similar among three groups of Flox SCs. While the IGF1 level of untreated cKO cells was much lower than that of Flox SCs; however, it was markedly increased by the treatment with squalene or cholesterol (Figure 10B). This meant the SCs secreted IGF1 is also regulated by FDFT1 through the squalene/cholesterol axis. Thereafter, the CM of each group was administered into the dorsal root ganglion (DRG) neurons cultures, and the neurons were fixed for immunostaining with Tuj1 antibody and the neurites of each neuron were quantified. Results indicated that both the number and length of neurites were similar among the three groups of Flox SCs. The neurons treated with the CM of cKO SCs had fewer and shorter neurites than those of Flox SCs, whereas the CMs of squalene or cholesterol‐treated cKO SCs markedly enhanced axon growth when compared to the untreated cKO SCs (Figure 10C). Moreover, we administered the LXR agonist T0901317 in vivo after sciatic nerve crush. Immunostaining and Western blotting analyses revealed that the T0901317 treatment significantly rescued the impaired regeneration of GAP43‐positive axons and the reduced protein levels of both GAP43 and IGF1 in the distal nerve of Fdft1 icKO mice (Figure 10D,E). These findings indicate that SCs FDFT1 plays a role in promoting axon growth via IGF1 paracrine.

## 3 Discussion

Overall, the principal findings of this study can be summarized as follows: 1) FDFT1 expression is downregulated in the early phase after sciatic nerve crush injury but is significantly upregulated during the regenerative phase; 2) Conditional knockout of FDFT1 in SCs impedes morphological and functional recovery after sciatic nerve crush injury in mice, underscoring the significance of FDFT1‐mediated cholesterol synthesis in peripheral nerve regeneration; 3) FDFT1 regulates the PI3K/AKT signaling pathway via a squalene/cholesterol/LXRα/IGF1/IGF1R/Rap1 axis, thereby playing a crucial role in the differentiation and remyelination of SCs; and 4) FDFT1 in SCs could promote axonal regeneration via an IGF1 paracrine mechanism.

During Wallerian degeneration in the early stage of nerve injury, the degrading myelin debris releases substantial free cholesterol into the microenvironment of the injured nerve [10]. Given that high extracellular cholesterol level is known to suppress the expression of endogenous cholesterol synthetic enzymes in the injured spinal cord [10, 37], we initially hypothesized that FDFT1 expression might be continuously downregulated in the injured nerve. However, a notable finding of this study is the biphasic expression pattern of FDFT1 following nerve injury. A personalized bioinformatic analysis of the published single‐cell RNA‐seq dataset GSE216665 indicated that *Fdft1* expression in rat sciatic nerve exhibited a biphasic change (down then up) after chronic constriction injury. Similarly, in the mouse crush injury model, we observed that FDFT1 protein was downregulated at 3–7 dpi and markedly upregulated by 14–28 dpi, a critical window for axonal regeneration and remyelination in the mice sciatic nerve crush injury model [38, 39]. The early downregulation of FDFT1 is reasonable to be interpreted as a feedback inhibition mechanism [12]. However, the subsequent upregulation of FDFT1 during regeneration is unexpected. Notably, our data show that FDFT1 knockout in Schwann cells leads to an upregulation of the c‐Jun and p75^NTR^ (the markers of immature Schwann cells), while it concurrently suppresses the expression of MAG and O1 (the markers of mature Schwann cells). These results indicate that FDFT1 plays a role in promoting the differentiation of Schwann cells and provide a functional context for the biphasic expression of FDFT1 observed in the crush‐injured nerve: its early downregulation may facilitate the Schwann cells transiting to dedifferentiate into a repair state, while its subsequent upregulation during the regenerative phase is critical for driving them to the state of re‐differentiation and remyelination. Notably, comparing to 14 dpi, the decline of FDFT1 at 28 dpi may correlate with the completion of the active remyelination phase in our model, as the demand for Schwann cells differentiation and new cholesterol synthesis diminishes.

Cholesterol is an essential structural component of biological membranes. Notably, myelin sheaths are among the cholesterol‐richest structures in the human body [1, 2]. During peripheral nerve development, cholesterol synthesized by SCs serves as the primary source for myelination, as evidenced by the hypomyelination observed in SC‐specific FDFT1‐deficient models [6]. It is therefore plausible that FDFT1‐mediated cholesterol biosynthesis in SCs also plays a critical role in nerve repair, particularly for remyelination. However, the situation of nerve regeneration following injury differs significantly from nerve development. In the absence of pre‐existing cholesterol reserves and with limited access to circulating cholesterol due to the blood‐nerve barrier [40], the myelination during nerve development is critically dependent on *de novo* cholesterol synthesis by SCs [6, 41]. In contrast, nerve injury leads to myelin disintegration, releasing a substantial pool of free cholesterol [10, 11]. Existing literatures indicate that a significant portion of this cholesterol can be reused for remyelination, as blocking the influx of blood‐derived cholesterol does not impair remyelination after injury [40, 42]. Naturally, the reutilization rate of cholesterol is impossible to reach 100%, indicating that the insufficient portion relies on the *de novo* synthesis. However, a perplexing paradox arises from the observation that cholesterol accumulation in the injured central nervous tissue is one of the main factors triggering oxidative stress and neuroinflammation [14, 43], thereby creating an unfavorable microenvironment for functional repair. This raises a critical question: Does cholesterol synthesized by SCs after nerve injury exacerbate cholesterol accumulation and neuroinflammation, or does it furnish the necessary components for nerve repair?

To precisely address this question, we developed a line of inducible conditional knockout (icKO) mice. The critical advantage of this model is that the knockout is triggered only upon tamoxifen administration [44], which was performed in adult mice just prior to preparing the nerve injury model. It can effectively circumvent the potential baseline difference between the icKO mice and their control littermates, thereby allowing us to unequivocally attribute the observed phenotypic outcomes specifically to the role of SCs FDFT1 deficiency in the nerve injury and repair. Our comprehensive analysis, which assessed the rate of axonal regeneration at 3 dpi, the extent of axonal regrowth and remyelination at 28 dpi, along with functional recovery measures, yielded a definitive answer: that the SCs FDFT1 deficiency significantly delayed both the structural and functional recovery of the injured nerve, which indicates that FDFT1‐mediated cholesterol biosynthesis in SCs plays an indispensable and beneficial role in the successful repair after peripheral nerve injury.

**FIGURE 11:**
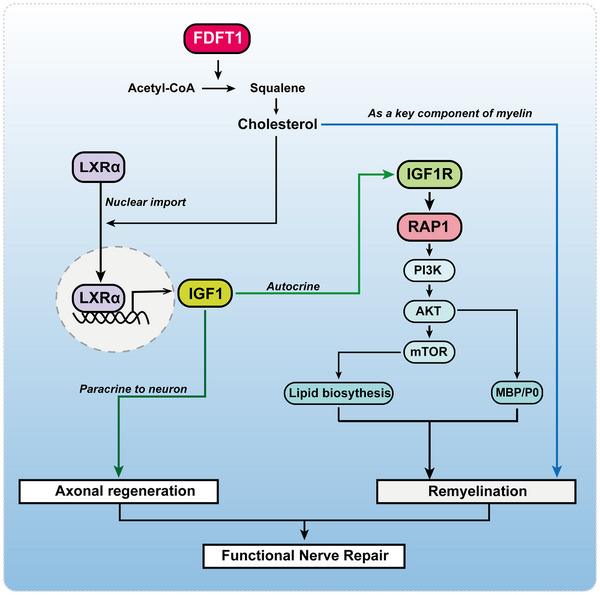
The schematic diagram illustrates the underlying mechanism of how SCs FDFT1 regulate functional peripheral nerve regeneration.

Beyond synthesizing cholesterol to work as a key structural component for myelin [41, 45], our data reveal a novel signaling mechanism (Figure 11). Herein, RNA‐seq was used to nominate plausible mechanistic pathways for FDFT1 function. Combined with subsequent focused validations, present study reveals that cholesterol synthesis modulates the LXRα/IGF1/IGF1R/Rap1/PI3K/Akt axis to influence Schwann cell differentiation and myelination. It is well known that AKT can upregulate myelin proteins such as P0 and MBP [30, 46] to enhance remyelination. Recently, FDFT1 has been documented to influence the biofunctions of certain tumor cells by regulating the PI3K/AKT pathway [26, 29]. However, none of the existing studies explained how FDFT1 regulates the PI3K/AKT pathway. Herein, based on transcriptomic sequencing, bioinformatics analysis and a series of confirming experiments, the collected findings lead us to believe that FDFT1 regulates PI3K/AKT dependent on the squalene/cholesterol/LXRα/IGF1/IGF1R/Rap1 axis. Thereafter, PI3K/AKT/mTOR regulates the SCs remyelination by promoting lipid synthesis [47, 48] as well as differentiation master factors (such as Krox20) and myelin‐related proteins (such as MBP and P0) [30, 49, 50]. These findings and hypotheses not only help us gain a deeper and more comprehensive understanding of the mechanism of SCs cholesterol biosynthesis in myelination, but also provide a theoretical basis and reasonable explanations for the role of FDFT1 in the PI3K/AKT signaling pathway [26, 27, 28, 29].

Previously, it is well known that SCs can secrete extracellular matrix and growth factors (such as NGF, NT‐3 and IGF1) to support axonal growth and regeneration [20, 51, 52, 53, 54, 55]. However, the role of FDFT1 and cholesterol in the SCs production of growth factors is still unknown. Transcriptomic and protein analyses confirmed that FDFT1‐deleted SCs secrete significantly less IGF1. Conditioned medium from the SC culture was less effective in supporting neurite outgrowth in the co‐cultured neurons. Crucially, this deficit was rescued by supplementing the SC culture with squalene or cholesterol, which increased IGF1 secretion and, consequently, the pro‐regenerative capacity of the conditioned medium. These evidences strongly suggest that the IGF1 downregulation in FDFT1 knockout SCs is related to the reduced synthesis of squalene and cholesterol. Moreover, existing evidence indicates that IGF1 can activate the PI3K/AKT/mTOR pathway to promote cholesterol synthesis and myelin protein production. Therefore, we propose that a bidirectional positive feedback loop exists between cholesterol synthesis and IGF1 signaling in SCs. This central axis likely serves to amplify and sustain the myelination program initiated by FDFT1‐mediated cholesterol synthesis.

### 3.1 Limitations of This Study

While this study establishes SC‐expressed FDFT1 as a critical orchestrator of peripheral nerve regeneration, certain limitations remain. First, the rescue via systemic squalene gavage may affect non‐SC compartments (e.g., macrophages, fibroblasts). Future SC‐specific genetic rescue models would be invaluable to conclusively confirm the cell‐autonomous nature of this pathway. Second, although our data show that key pathway components (FDFT1, LXRα, IGF1) are expressed in a human Schwann cell line (Figure S12), the direct involvement and therapeutic relevance of this axis in human nerve repair require further validation. Finally, to our knowledge, no clinical trials directly targeting FDFT1 exist. Future work should therefore focus on pharmacologically modulating the FDFT1‐cholesterol‐IGF1 axis, followed by rigorous preclinical and clinical evaluation to assess its therapeutic potential for peripheral nerve injury.

## 4 Conclusions

Present findings demonstrate that FDFT1‐mediated cholesterol biosynthesis in SCs plays a crucial role in peripheral nerve regeneration, which is dependent on its dual roles, from being a structural component to acting as metabolite signaling for IGF1‐dependent axonal regeneration and remyelination.

## 5 Experimental Section

### 5.1 Establishment of Transgenic Mice

The *Fdft1* ^flox/flox^ mice, *Plp* ^CreERT2^ mice and *Dhh* ^Cre^ mice were obtained from Cyagen Biosciences Inc. (Suzhou, China). SCs Tamoxifen‐induced FDFT1 knockout mice (*Fdft1* ^flox/flox^; *Plp* ^CreERT2^, *Fdft1* icKO) were generated by crossing *Fdft1* ^flox/flox^ mice with *Plp* ^CreERT2^ mice, which were specifically designed for peripheral nerve injury models. *Fdft1* ^flox/flox^; *Dhh* ^Cre^ (*Fdft1* cKO) mice were generated by crossing *Fdft1* ^flox/flox^ mice with *Dhh* ^Cre^ mice, primarily utilized for the primary culture of SCs. All mice were maintained on a C57BL/6J background and kept under specific pathogen‐free conditions, adhering to a regular 12 h:12 h light: dark cycle. All animal procedures were conducted in accordance with the research ethics guidelines of the Southern Medical University Animal Care and Use Committee (approval No. SMUL2021181).

### 5.2 Primary Culture of SCs

The primary culturing of SCs was performed as previously described [56, 57]. Briefly, SCs were isolated from the spinal nerves and sciatic nerves of neonatal mice (5–6 days old) by digesting the tissues with 0.25% Trypsin‐EDTA at 37°C for 30 min to obtain a single‐cell suspension. The cells were then cultured in DMEM/F12 supplemented with 10% fetal bovine serum (FBS) in a poly‐L‐lysine‐coated petri dish. After 24 h of incubation, the medium was supplemented with 10 µM of cytosine arabinoside to eliminate fibroblasts for 48 h. Subsequently, the medium was replaced with DMEM/F12 containing 10% FBS, 3 µM forskolin, 10 ng mL^−1^ heregulin‐β1, 4 mM L‐glutamine, 100 U mL^−1^ penicillin, and 100 mg mL^−1^ streptomycin to promote cell expansion.

### 5.3 SCs Differentiation

Differentiation was induced as previously described [58]. Briefly, SCs were cultured in a non‐proliferative medium (DMEM/F12 containing 3% FBS, 100 U mL^−1^ penicillin, and 100 mg mL^−1^ streptomycin) for 24 h. Subsequently, SCs were treated with DMEM/F12 containing 3% FBS, 1 mM dibutyryl adenosine 3',5'‐cyclic monophosphate (dbcAMP) and 20 ng mL^−1^ heregulin‐β (HRG1β) for 72 h to induce a differentiated phenotype, followed by fixation with 4% paraformaldehyde (PFA) for immunofluorescence staining.

### 5.4 Cell Cytotoxicity Assay

Schwann cells were plated in a 96‐well plate and allowed to adhere overnight. The medium was then replaced with fresh medium containing serial concentrations of squalene or cholesterol. After 24 h CCK‐8 solution was added to the wells for 2 h and the absorbance was measured at 450 nm.

### 5.5 Culture of DRG Neurons and Treatment

Dorsal root ganglion (DRG) neurons were cultured based on previous report [59, 60]. Briefly, DRGs were isolated from wild‐type C57BL/6J neonates (postnatal days 1–2). The epineurium of each ganglion was carefully stripped in cold Hank's balanced salt solution under a stereomicroscope. Subsequently, DRGs were dissociated using 0.125% Trypsin‐EDTA digestion at 37°C for 30 min. Following digestion and dissociation, the DRG neurons were centrifuged for 10 min at 1000 rpm and resuspended in DMEM/F12 containing 1% FBS and 100 mg mL^−1^ penicillin‐streptomycin.

### 5.6 Culture of Human Schwann Cell Line sNF96.2

sNF96.2 was purchased from IMMOCELL (Xiamen, Fujian, China). In brief, the cells were cultured in DMEM containing 10% FBS, 100 U mL^−1^ penicillin, and 100 mg mL^−1^ streptomycin at 37°C with 5% CO_2_ [61]. For IF staining, cells were placed on coverslip and fixed in cold 4% PFA.

### 5.7 Genotyping

Genomic DNA was utilized for genotyping as follows: Thermocycler conditions for all genotypes consisted of an initial denaturation step at 94°C for 3 min, followed by 33 cycles of denaturation at 94°C for 30 s, annealing at 57°C for 30 s, and extension at 72°C for 30 s, concluding with a final extension at 72°C for 5 min. PCR reaction mixtures were prepared using Taq‐Mix, and the primers were detailed in Table S1.

### 5.8 Tamoxifen Administration

Tamoxifen was dissolved in corn oil at a concentration of 20 mg mL^−1^ and stored at 4°C. Adult *Plp* ^CreERT2^ mice and *Fdft1* icKO mice (aged 8 weeks) received daily intraperitoneal injections of tamoxifen at a dosage of 100 mg kg^−1^ for 5 consecutive days as described previously [44]. Ten days after the last injection, the mice were subjected to prepare the sciatic nerve injury model as following.

### 5.9 Sciatic Nerve Crush Injury

Mice were anesthetized using isoflurane. The sciatic nerve crush injury was established as previously described [51]. Bilateral sciatic nerves were bluntly exposed, and a crush injury was performed 0.5 cm distal to the sciatic notch using a fine, smooth, and straight hemostat for 2 min. In the sham operation, the sciatic nerves were exposed without inflicting a crush injury. Axonal regeneration was assessed at 3 days post‐injury (3 dpi), while remyelination and functional recovery (including behavioral assessments and electrophysiological tests) were evaluated at 28 dpi. For measuring Wallerian degeneration and the function of macrophages, bilateral sciatic nerves were transected and samples were gained at 5 dpi.

### 5.10 Administration of Squalene

The in vivo supplementation dosage of squalene was determined based on Berghoff's [24]. An equivalent dose conversion was performed according to a previous article [62], resulting in a calculated daily squalene intake of 0.89 mg g^−1^. Briefly, squalene was dissolved in corn oil. The gavage solution was prepared based on an average mouse body weight of 25 g and a daily gavage volume of 0.1 mL per mouse. Administration via intragastric gavage [24] began on the day of sciatic nerve injury and continued daily until tissue collection for analysis at 3dpi or 28 dpi. For the in vitro study, Squalene (200 µM) was supplemented into the culture medium to assess its ability to successfully reverse alterations in SC's differentiation.

### 5.11 Administration of LXR Agonist and Inhibitor

To assess its effects on regenerating axonal growth, LXR agonist T0901317 was administered in vivo as previously described [63, 64]. The compound was prepared at a concentration of 5 mg mL^−1^ in a vehicle of 5% DMSO in normal saline. Following sciatic nerve crush injury, mice received daily intraperitoneal (*i.p*.) injections of T0901317. Tissue samples were harvested at 3 dpi for subsequent analysis. For in vitro experiment, 10 µM T0901317 was selected based on its effective use in prior studies investigating LXR activation in glial cells [36]. To further validate the linearity of the proposed pathway, the LXR antagonist GSK2033 was added after treating squalene in icKO primary Schwann cells. A concentration of 2 µM GSK2033 was selected for the in vitro experiment, based on a previous study [65].

### 5.12 Western Blotting

Protein extracts from cultured cells or tissues were lysed in RIPA lysis buffer containing a protease inhibitor cocktail. The extracts were then separated on SDS‐PAGE gels and transferred to PVDF membranes. Following this, the membranes were blocked with 5% non‐fat milk (0.5% Tween‐20 in PBS) for 1 h at room temperature (RT). After incubation with primary antibodies overnight at 4°C, the blots were incubated with HRP‐conjugated secondary antibodies at RT for 2 h, and were subsequently visualized using enhanced chemiluminescence. The antibodies used were provided in Table S1.

### 5.13 Immunofluorescence and Hematoxylin&Eosin (H&E) Staining

For immunofluorescent staining, samples were permeabilized and blocked in 0.5% Triton X‐100 for 30 min, followed by blocking in 5% fish gelatin containing 0.3% Triton X‐100 for 1 h at RT. Primary antibodies were then applied and incubated overnight at 4°C. After washing with PBS, sections were incubated with secondary antibodies conjugated to Alexa Fluor 488 or 568 for 2 h at RT, washed three times in PBS, and subsequently mounted in Antifade Mounting Medium (with DAPI).

For FluoroMyelin staining, samples were washed three times in PBS and FluoroMyelin was applied. After incubation for 30 min at RT in the dark, samples were washed three times in PBS and mounted in Antifade Mounting Medium (with DAPI).

For H&E staining, the gastrocnemius muscles of mice were fixed in 4% PFA overnight at 4°C and embedded in paraffin prior to sectioning. Sections were stained with H&E and photographed under bright‐field microscopy.

IF and HE staining were performed on 10‐µm‐thick sections, while α‐BTX staining analysis of the gastrocnemius muscle was conducted on 100‐µm‐thick sections. Once NF‐positive nerve fibers touch/colocalize with BTX, we determined the NMJ had been reinnervated [66, 67]. The information regarding the antibodies used was presented in Table S1.

### 5.14 Cholesterol Replenishment and Measurement

For the replenishment of cellular cholesterol, complexes of cholesterol and methyl‐β‐cyclodextrin (MβCD) were prepared following a modified version of a previously described protocol [68]. Briefly, an 8 mL aqueous solution of 50 mM MβCD and 1 mL of anhydrous ethanol containing 0.018 g cholesterol were first mixed at room temperature. The mixture was then incubated in a 37°C water bath for 7 h and finally passed through a 0.22 µm filter. The MβCD‐cholesterol complexes were stored at −20°C and added to cell culture medium to achieve a final concentration of 1 µg mL^−1^.

Filipin III staining was utilized to index the level of free cholesterol as previously reported. [69, 70] After rinsing in PBS, the samples were incubated in the dark with 1 mg mL^−1^ Filipin III in PBS for 30 min at RT. The actin cytoskeleton (F‐actin) was visualized using fluorescent‐conjugated phalloidin to delineate the cell outline.

### 5.15 Transmission Electron Microscopy (TEM)

The sciatic nerve and optic nerve were isolated, fixed, dehydrated, and embedded in anhydrous acetone/embedding solution as described previously [51, 71]. The samples were then sectioned into 70 nm slices and dyed with uranium and plumbum. Images were randomly captured using a transmission electron microscope. Ten random fields from each section were analyzed to determine the area of myelinated axons, the thickness of the myelin sheath, and the G‐ratio (the ratio of the inner to the outer diameter of the myelin sheath of a myelinated axon) utilizing Image J software. For the number of myelinated axons, a minimum of three images (25 × 25 µm^2^) from each mouse were randomly selected for blinded quantification.

### 5.16 RNA‐Sequencing and Bioinformatic Analysis

RNA‐Sequencing (RNA‐seq) was performed to analyze the differential expression of genes between Flox SCs and cKO SCs. Total RNA was extracted using Trizol. The mRNA library was prepared using the Digital Gene Expression Tag Profile kit, and sequencing was performed with Oebiotech's sequencing‐by‐synthesis method. The criteria for identifying differentially expressed genes (DEGs) included a false discovery rate (FDR) of < 0.01, fold changes of < 0.5 or > 2.0, and *p*‐value of < 0.05. Differentiation‐related genes were retrieved from the GeneCards database. The intersection of differentially expressed genes (with fold changes of < 0.5 or > 2.0 and *p* < 0.05) and differentiation‐related genes was analyzed for KEGG pathway enrichment.

### 5.17 Enzyme‐Linked Immunosorbent Assays

An enzyme‐linked immunosorbent assays (ELISA) assay was conducted to quantify the levels of IGF1 secreted by SCs. The cells were incubated in culture medium at 37°C in a 5% CO_2_ atmosphere. After 48 h, the supernatant was harvested, and the concentration of IGF1 was measured using a mouse‐specific IGF1 ELISA kit.

### 5.18 Rota‐Rod Test

Following two training sessions at 26 dpi and 27 dpi, the mice were placed on the apparatus, which accelerated from 5 to 40 rpm over a period of 120 s. Mice were subjected to three trials with 30 min intervals, and the latency to fall off the rod was recorded as an index of motor capacity. Data were acquired at 28 dpi, and the motor capacity index was calculated based on the average of the three trials.

### 5.19 Electrophysiological Test

The electrophysiological evaluation was conducted as previously described [72]. After anesthetizing the mice with tribromoethanol, the sciatic nerves were exposed. The compound muscle action potential (CMAP) was recorded using an electrophysiological digitizer (Axon Digidata 1550 Digitizer, Molecular Devices). Recording electrodes were inserted into the intrinsic foot muscle, and stimulation was applied 3 mm proximal to the injury site. Amplitude and latency were measured and analyzed after conducting 10 trials.

### 5.20 Footprint Analysis

Gait characteristics were measured using footprint analysis. In short, the hind feet of mice were painted with washable, nontoxic ink before walking through an 80 cm in length and 10 cm in width chamber. The footprints were collected by white paper fitted to the floor and measured with ImageJ. At least 3 representative and legible steps were analyzed for each animal. Stride was measured from one footprint to the next step on the same side, sway was measured by the horizontal width between left and right foot, and stance was measured by the diagonal distance between left and right feet [73]. The Sciatic functional index (SFI) was calculated by following equation below [74]. E: Experimental side, N: Normal side, PL (Print length): the distance between third toe to the heel, TS (Toe spreading): the distance between first toe to fifth toe, IT (Intermediary toe spreading): the distance between the second toe to the fourth toe.

(1) $$\begin{array}{ccc} SFI & = & -38.3\left(\frac{EPL-NPL}{NPL}\right)+109.5\left(\frac{ETS-NTS}{NTS}\right)\\ & & +\;13.3\left(\frac{EIT-NIT}{NIT}\right)-8.8 \end{array}$$

### 5.21 Statistical Analysis

All data were presented as the mean ± SD. All statistical analyses based on GraphPad Prism 10.0 software (GraphPad Software, San Diego, CA, USA). Statistical analysis was performed using one‐way ANOVA or two‐way ANOVA for multiple comparisons. The differences between the two groups were tested using Two‐tailed Student's *t*‐test, “ns” indicating no significance, *p < 0.05 was considered statistically significant. All quantitative assessments, including data collection and statistical analysis, were conducted by investigators who were unaware of the group assignment.

## Conflicts of Interest

The authors declare no conflicts of interest.

## Supporting information




**Supporting File 1**: advs73654‐sup‐0001‐SuppMat.docx.

**Supporting File 2**: advs73654‐sup‐0002‐Data.zip.

## Data Availability

The data that support the findings of this study are available from the corresponding author upon reasonable request.
