# Supplementary material for: Schwann Cell Synthesized Cholesterol Orchestrates Peripheral Nerve Regeneration via Structural and IGF1‐Dependent Signaling Mechanisms
Source: Adv Sci (Weinh). 2026 Jan 4;13(16):e20323. doi: 10.1002/advs.202520323 (PMC13042600; doi:10.1002/advs.202520323)
Supplement: Supplementary file 1 — Supporting File 1: advs73654‐sup‐0001‐SuppMat.docx. [file ADVS-13-e20323-s002.docx]

Supporting Information

**Schwann Cell Synthesized Cholesterol** **Orchestrates Peripheral Nerve Regeneration via Structural and IGF1-dependent Signaling Mechanisms**

*Shuyi Xu^1,2, #^, Ye He^1,2,#^, Ying Zou^1,3, #^, Mengyao Zhao^1,2^, Jiaqi Zhang^1^, Yizhou Xu^1^, Jiale Cai^1,2^, Xiongbo Luo^1,2^, Xinrui Ma^1,2^, Saini Wu^1^, Yuling Huang^1^, Xianghai Wang^1,2^, Jiasong Guo^1,2,^**

**Supplemental Figures**


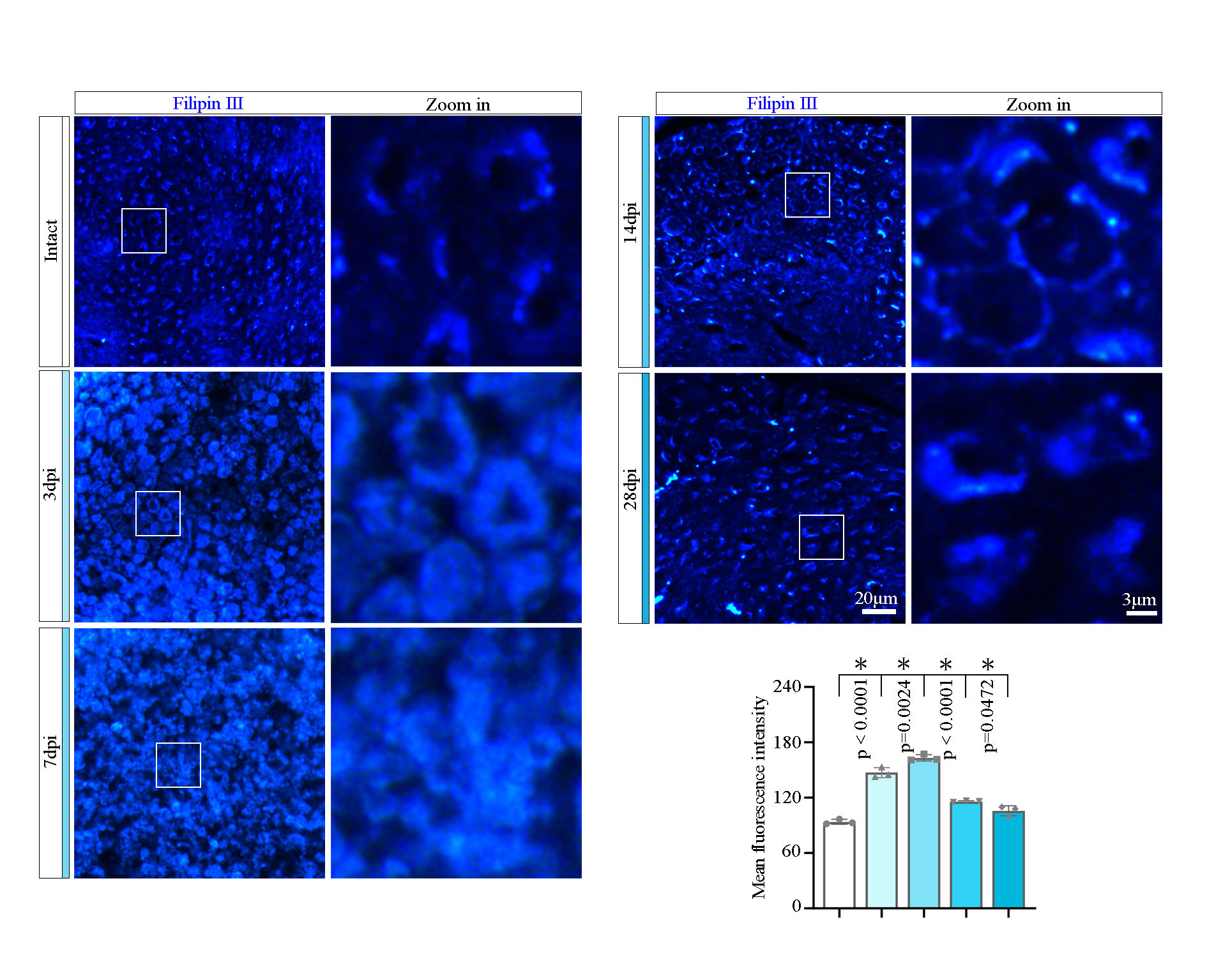


**Figure S1.** Filipin III staining and quantification on the transverse sections of the injured nerves show the free cholesterol levels exhibiting a biphasic response following sciatic nerve crush injury at 3, 7, 14 and 28 dpi. Scale bar = 20 μm, zoom in, 3 μm (n = 3). Data are presented as mean ± SD, One-way ANOVA, “ns” indicating no significance, *p < 0.05.

**
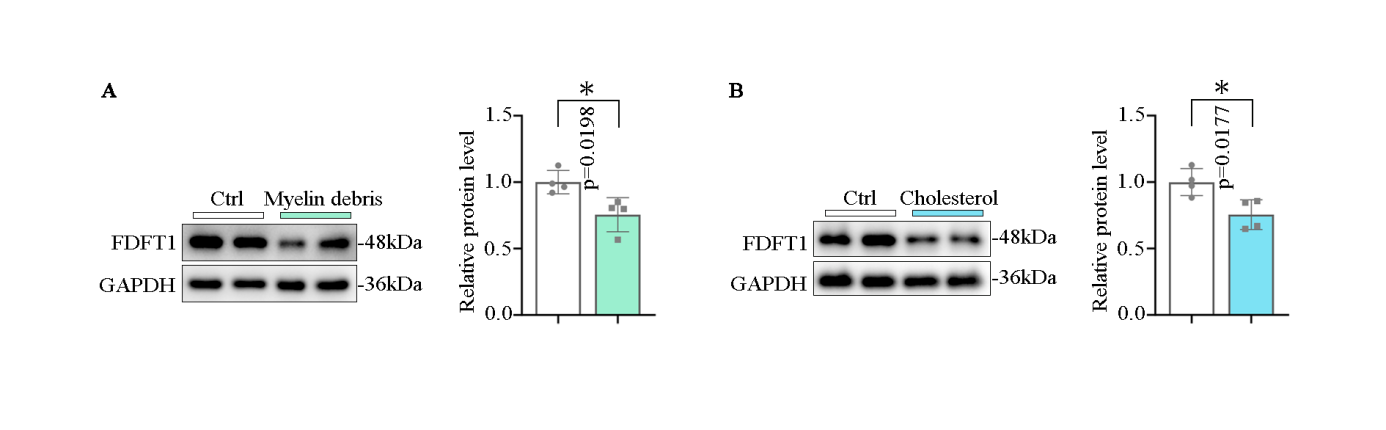
**

**Figure S2.** Western blot analysis of FDFT1 expression after myelin debris and cholesterol administration (n = 4). Data are presented as mean ± SD, Two-tailed Student's *t*-test, “ns” indicating no significance, *p < 0.05.

**
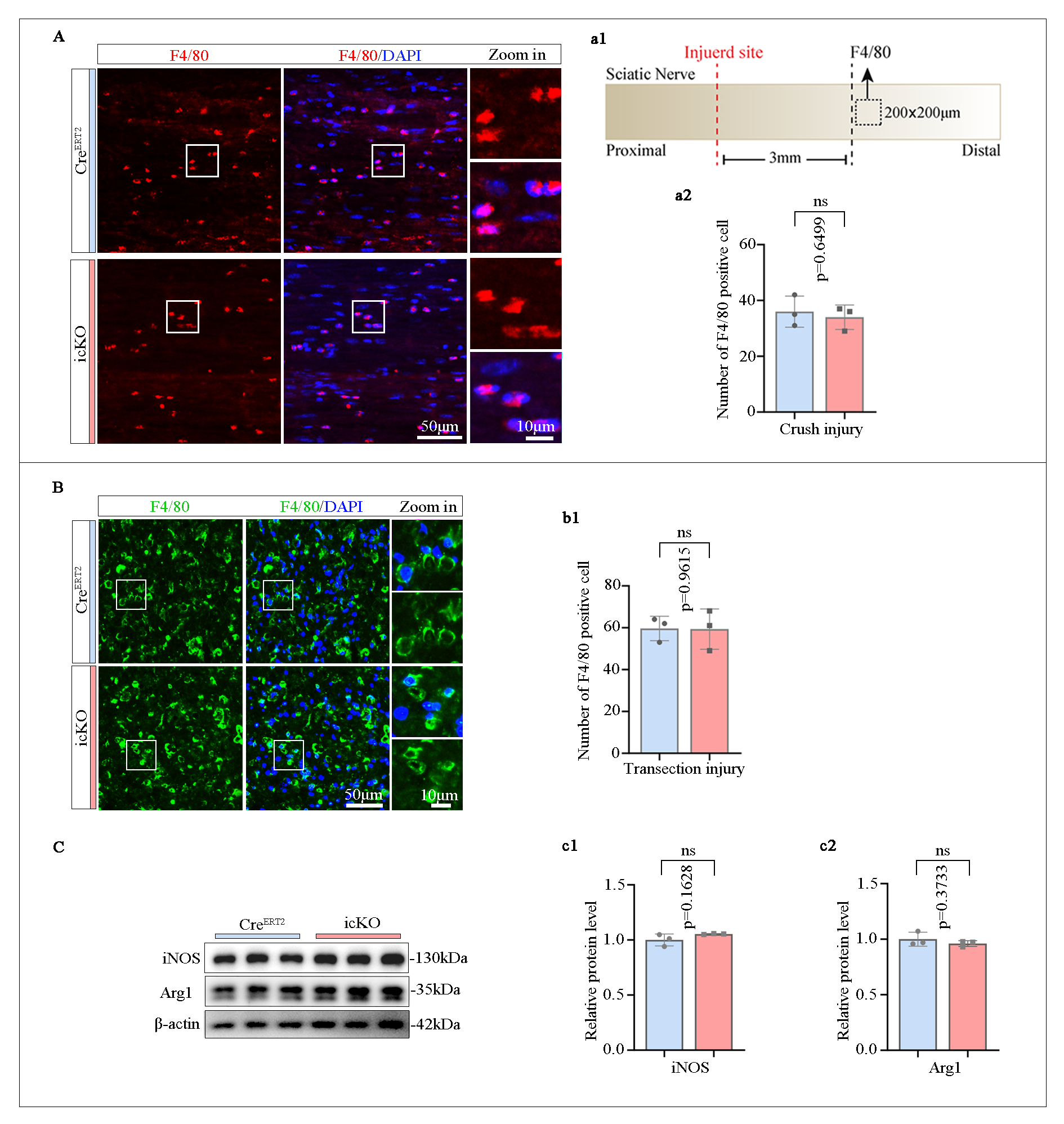
**

**Figure S3.** FDFT1 deficiency in SCs does not affect macrophages’ infiltration and polarization in the injured sciatic nerve. A) F4/80 immunostaining shows that the number of macrophages (a2) in the crushed nerves (a1) at 3 dpi is similar between the Cre^ERT2^ group and icKO groups. Scale bar = 50 μm, zoom in, 10 μm, (n = 3). B) Immunostaining shows the cross-section of sciatic nerve transection injury at 5 dpi. Quantification of F4/80^+^ cells shows in (b1), (n = 3). C) Western blotting and quantification (c1, c2) of iNOS and Arg1 expression at sciatic nerve transection injury at 5 dpi. Scale bar = 50 μm, zoom in, 10 μm, (n = 3). Data are presented as mean ± SD, Two-tailed Student's *t*-test, “ns” indicating no significance, *p < 0.05.

**
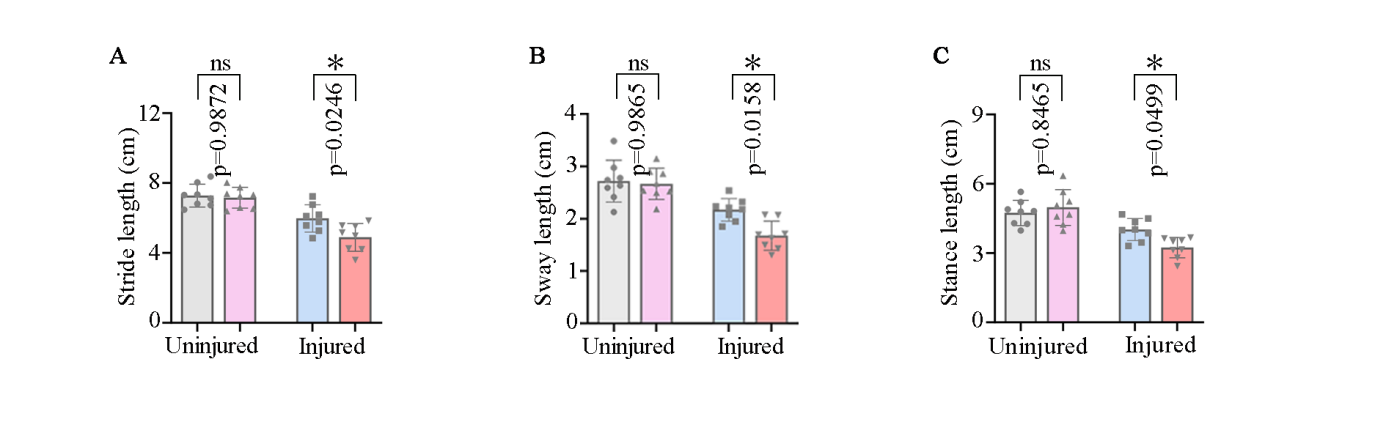
**

**Figure S4.** Quantification of hind limb stride length analysis, including stride length, sway length, and stance length (n = 8). Data are presented as mean ± SD, Two-way ANOVA, “ns” indicating no significance, *p < 0.05


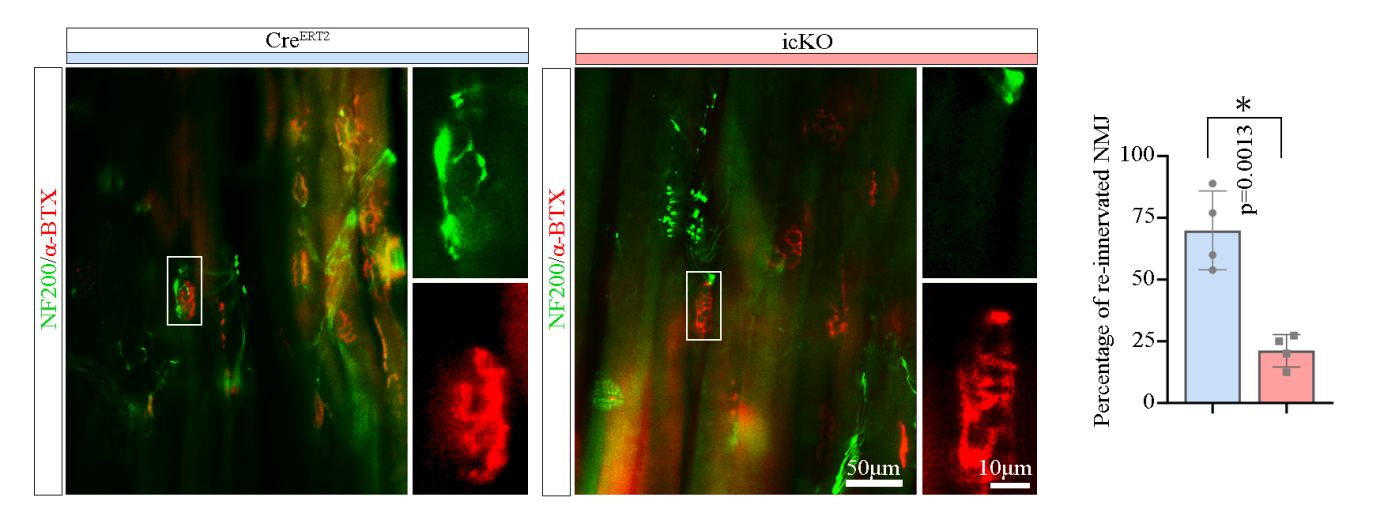


**Figure S5.** α-BTX and NF200 immunostaining of the gastrocnemius muscle, and quantified the percentage of re-innervated NMJ. Scale bar = 50 μm, zoom in, 10 μm, (n = 4). Two-tailed Student's *t*-test, “ns” indicating no significance, *p < 0.05.


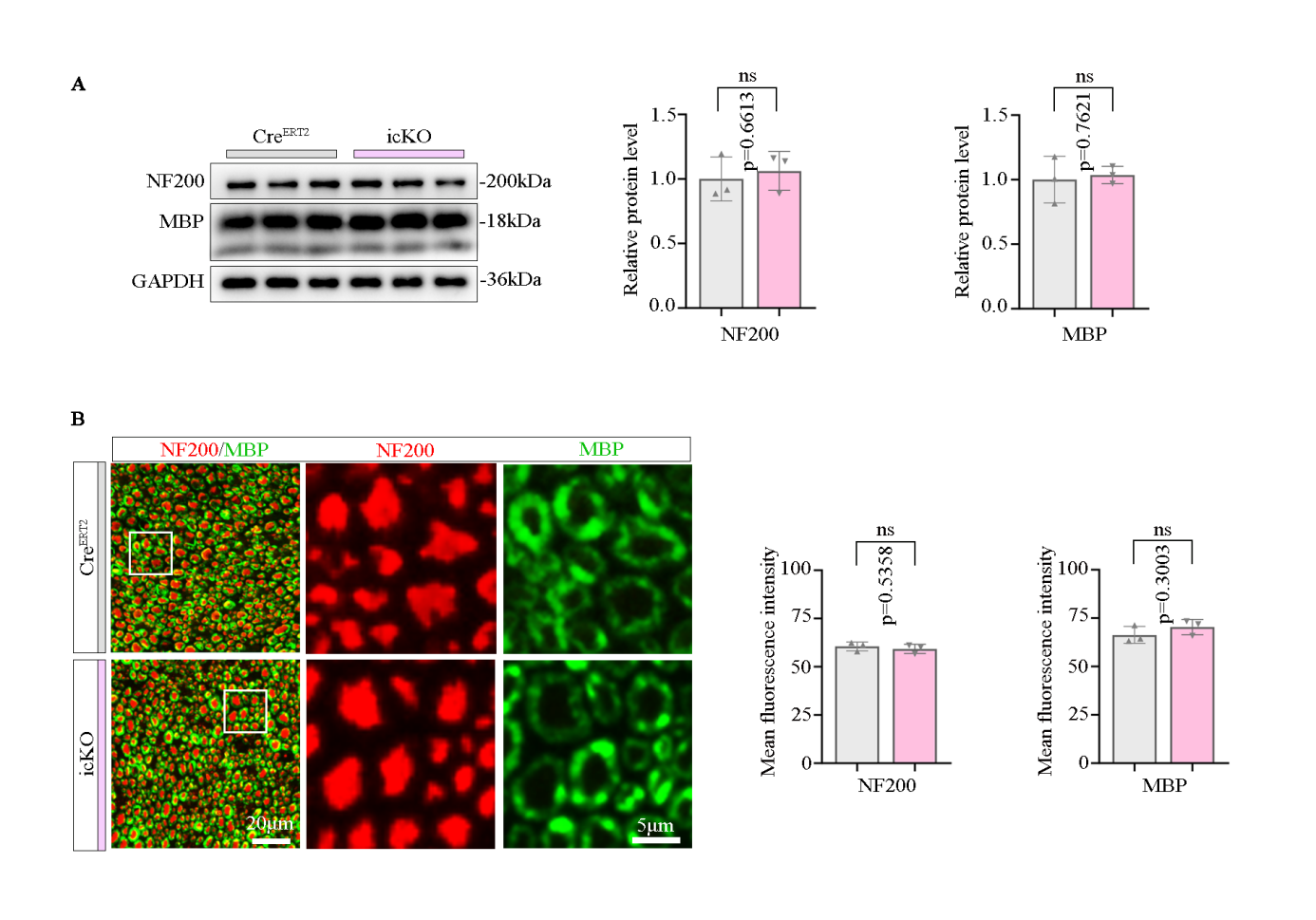


**Figure S6.** No significant differences were observed in the intact sciatic nerve’s axon or myelin between Cre^ERT2^ mice and icKO mice. A) Western blot analysis of NF200 and MBP expression (n = 3). B) Immunostaining and quantification of MBP and NF200. Scale bar = 50 μm, zoom in, 10 μm, (n = 3). Two-tailed Student's *t*-test, “ns” indicating no significance, *p < 0.05.

**
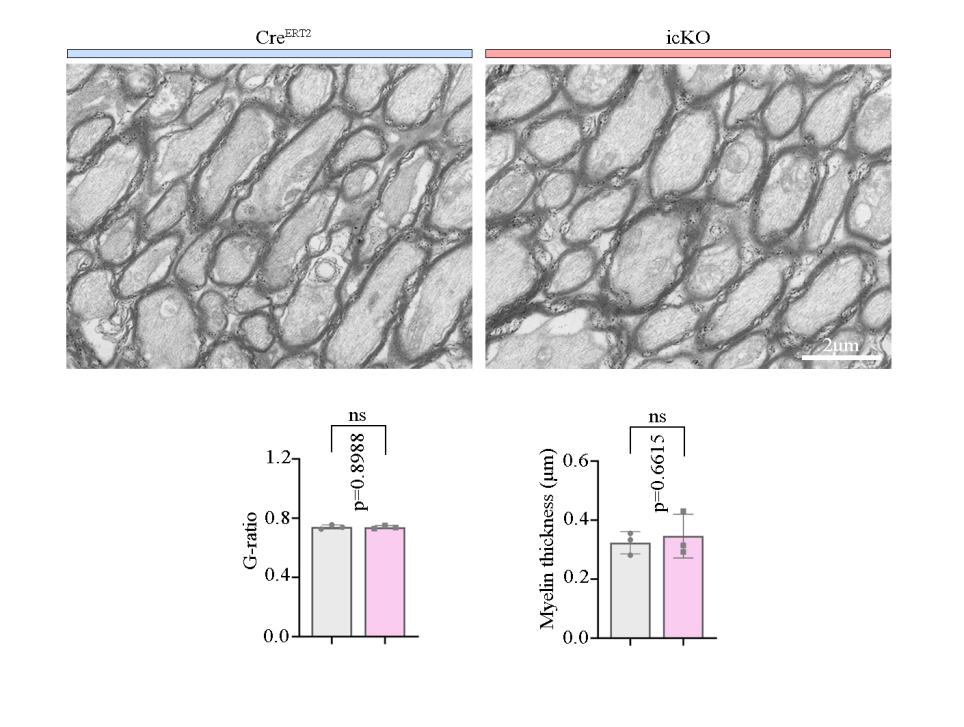
**

**Figure S7.** TEM analysis shows the G-ratio and myelin thickness of intact optic nerve have no change between Cre^ERT2^ mice and icKO mice. Scale bar = 2 μm, (n = 3). Two-tailed Student's *t*-test, “ns” indicating no significance, *p < 0.05.


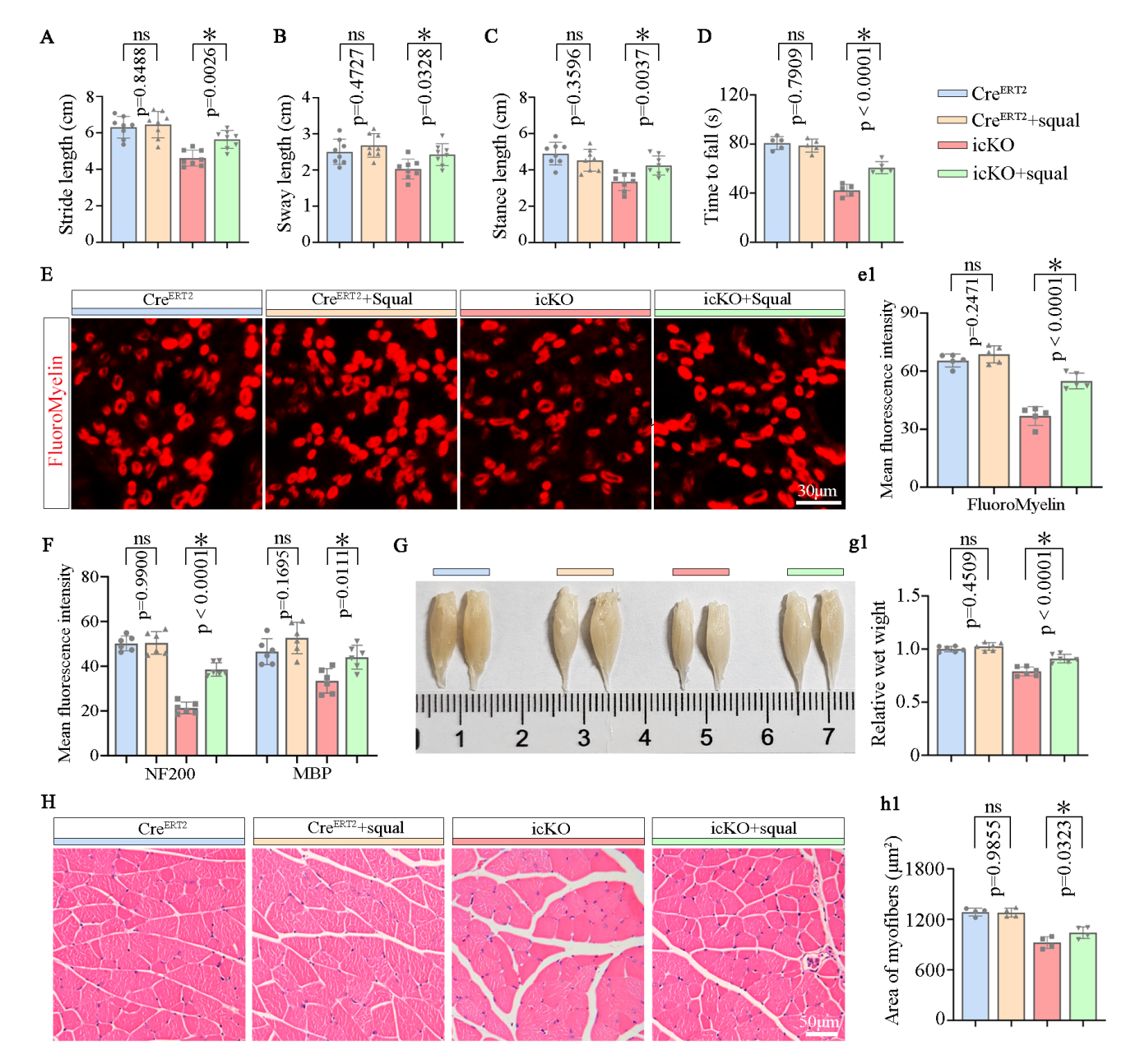


**Figure S8.** The administration of squalene *in vivo* promotes morphological and functional recovery after sciatic nerve injury at 28 dpi. A-C) Quantification of the hind limb stride length. Scale bar = 1 cm. D) Rota-rod test at accelerating speed (5-40 rpm over 120 s), (n = 8). E) Fluoromyelin staining and quantification (e1) assessed myelin content. Scale bar = 30 μm, (n = 5). F) Quantification of NF200 (red) and MBP (green) mean fluorescence intensity on the transverse sections of the nerve 3 mm distal to the lesion site at 28 dpi (n = 6). G) Gross morphology and wet weight ratio (g1) of the gastrocnemius muscle (n = 6). H) HE staining and fiber cross-area quantification (h1) of the gastrocnemius muscle. Scale bars, 50 μm, (n = 4). Data are presented as mean ± SD, Two-way ANOVA, “ns” indicating no significance, *p < 0.05.


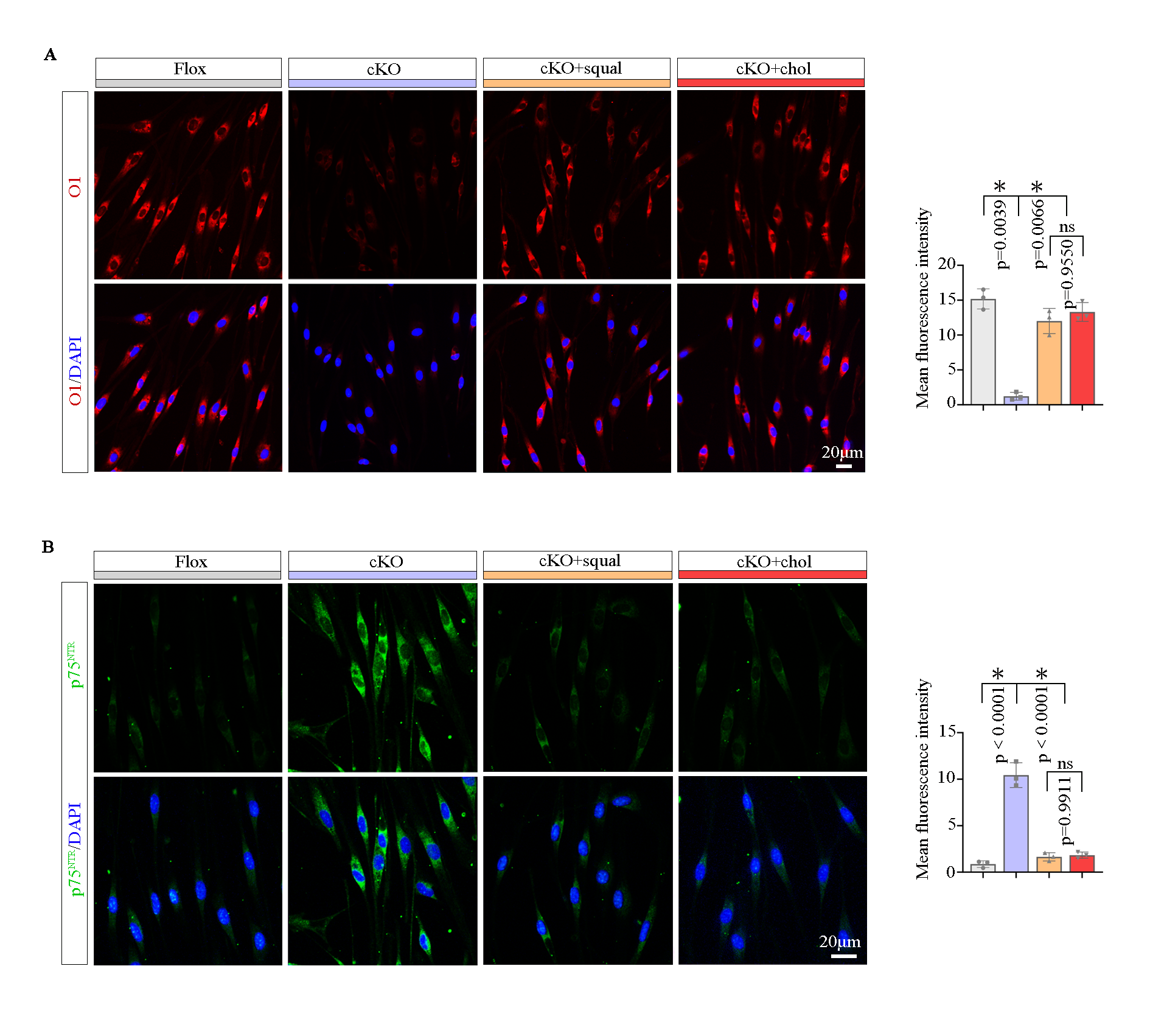


**Figure S9.** The expression of O1 and p75^NTR^ patterns can be markedly rescued by the supplementation of squalene or cholesterol in differentiated cKO SCs. A) Immunostaining and quantification of O1 expression. Scale bar = 20 μm, (n = 3). B) Immunostaining and quantification of p75^NTR^ expression. Scale bar = 20 μm, (n = 3). Data are presented as mean ± SD, Two-way ANOVA, “ns” indicating no significance, *p < 0.05.


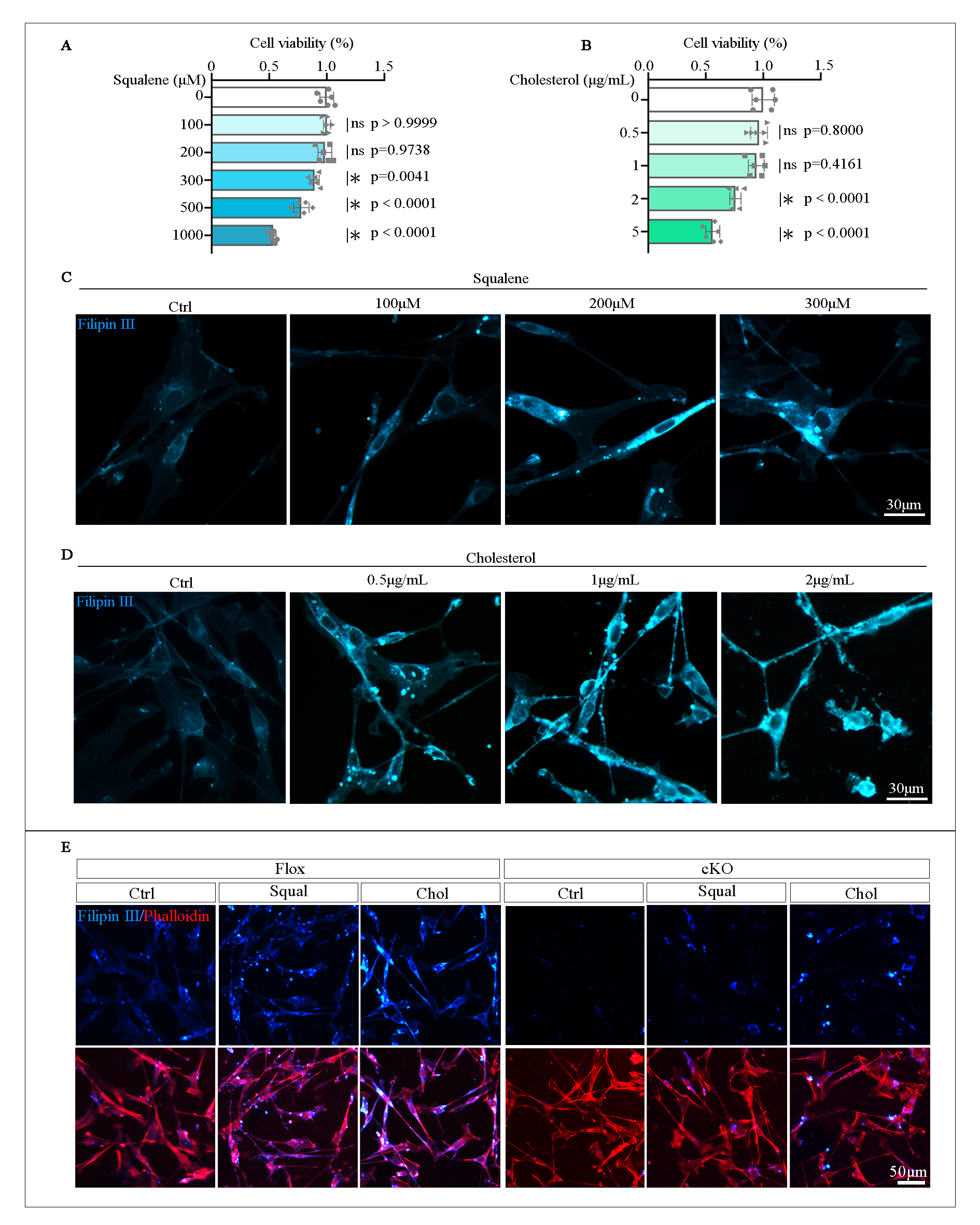


**Figure S10.** Determination of squalene and cholesterol concentrations for *in vitro* administration. A, B) CCK-8 assay determines the optimal concentrations of squalene and cholesterol. C, D) Filipin III staining verifies the concentrations of squalene and cholesterol. Scale bar = 30 μm. E) Filipin III staining shows the cholesterol levels within Flox and cKO primary SCs with or without squalene and cholesterol treatments. Data are presented as mean ± SD, Two-way ANOVA, “ns” indicating no significance, *p < 0.05.


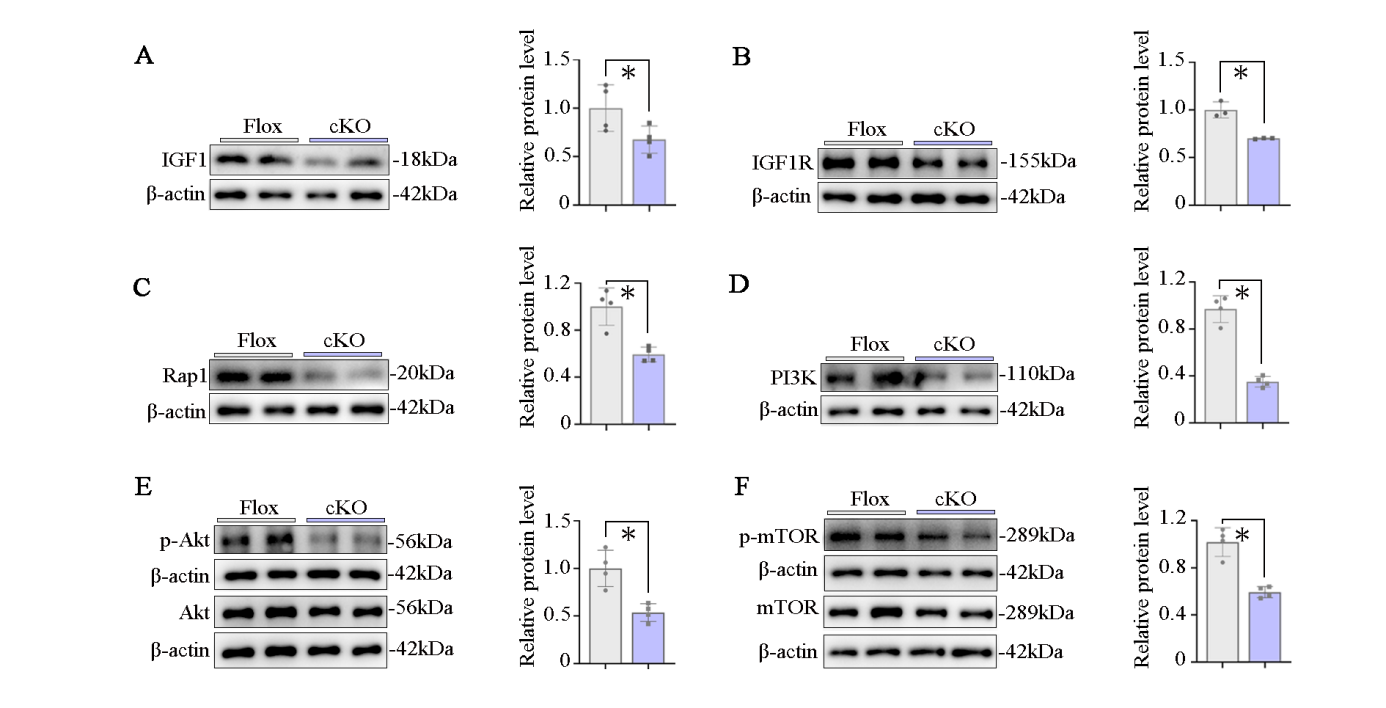


**Figure S11.** Western blot analysis reveals that FDFT1 cKO inhibits the protein levels of IGF1, Rap1, PI3K, p-Akt, and p-mTOR in primary cultured SCs (n = 4). Two-tailed Student's *t*-test, “ns” indicating no significance, *p < 0.05.


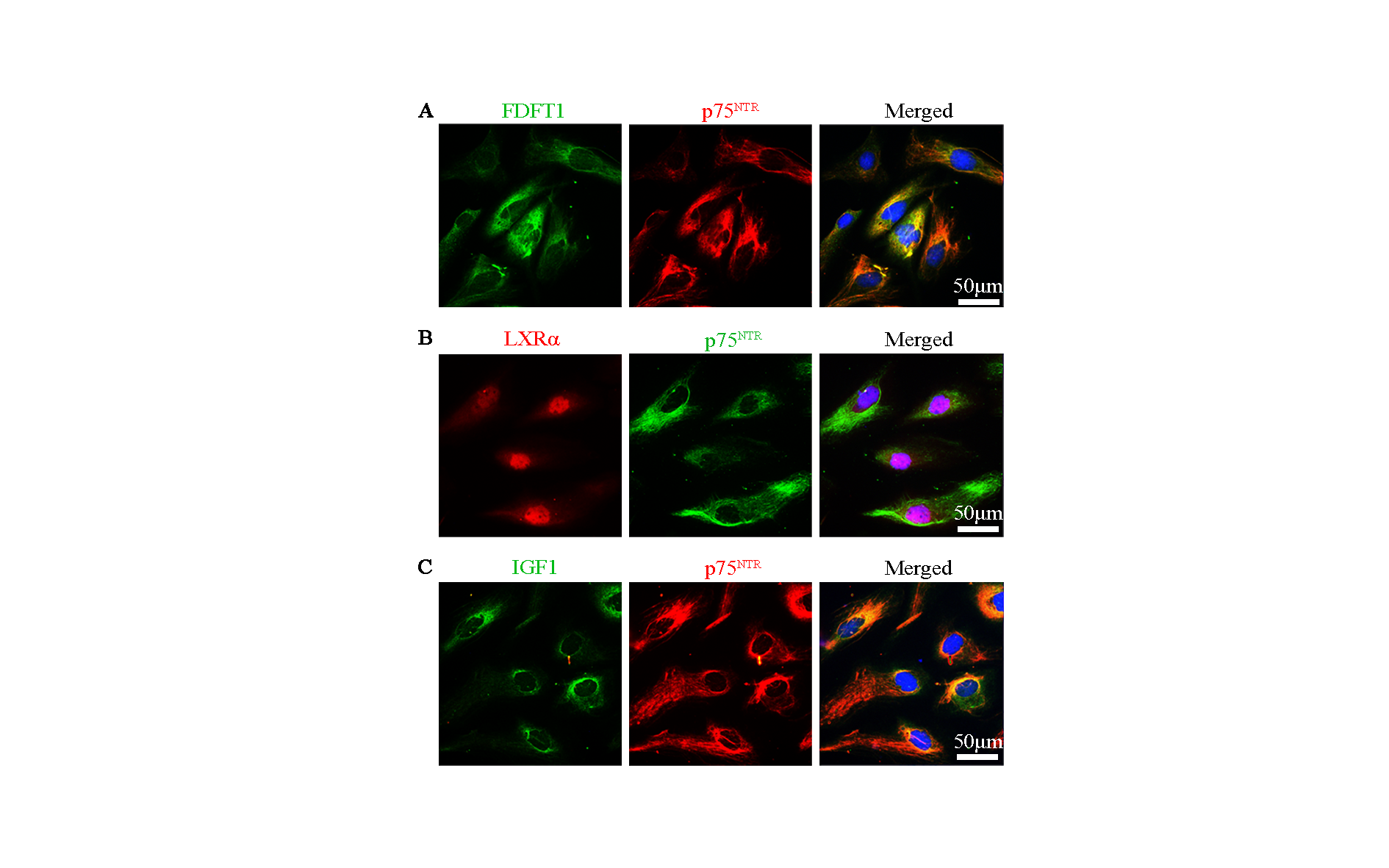
**Figure S12.** Immunostaining indicate the FDFT1, LXRα, and IGF1 are expressed in the human Schwann cell line sNF96.2 (marked with p75^NTR^ ). Scale bar = 50 μm.

**Supplemental Tables**

Table S1. Details of material and antibodies used in the present study.

| **EAGENT or RESOURCE** | **SOURCE** | **IDENTIFIER** |
| --- | --- | --- |
| **Antibodies** | | |
| **Rabbit anti-GAPDH** | Abcam | Cat#ab8245  WB(1:2000) |
| **Mouse anti-β-Actin** | Abclonal | Cat#AC006  WB(1:2000) |
| **Rabbit anti-FDFT1** | Proteintech | Cat#13128-1-AP  WB(1:800), IF(1:200) |
| **Mouse anti-S100** | Proteintech | Cat#66616-1-Ig  IF(1:200) |
| **Rabbit anti-GAP43** | Abcam | Cat# ab16053  WB(1:800), IF(1:200) |
| **Rabbit anti-NF200** | Sigma-Aldrich | Cat# N0142  WB(1:800), IF(1:200) |
| **Rabbit anti-P0** | Millipore | Cat#ABN363  IF(1:200) |
| **Mouse anti-MBP** | Calbiochem | Cat#NE1018  WB(1:800), IF(1:200) |
| **Rat anti-F4/80** | Proteintech | Cat#18705-1-AP  IF(1:200) |
| **Rabbit anti-MAG** | Abcam | Cat#AB89780  WB(1:500), IF(1:200) |
| **Rabbit anti-O1** | Invitrogen | Cat#50-6506-82  IF(1:100) |
| **Mouse anti-c-Jun** | BD Bioscience | Cat#610326  IF(1:100) |
| **Rabbit anti-p75^NTR^** | Cell signaling technology | Cat#8238T  IF(1:100) |
| **Mouse anti-p75^NTR^** | Abcam | Cat#ab245134  IF(1:100) |
| **Rabbit anti-IGF1** | Abclonal | Cat#A24744  WB(1:800) |
| **Rabbit anti-IGF1R** | MedChemExpress | Cat#HY-P80825  WB(1:800) |
| **Rabbit anti-Rap1** | Abclonal | Cat#A9725  WB(1:800) |
| **Rabbit anti-PI3K** | Abclonal | Cat#A0265  WB(1:800) |
| **Rabbit anti-AKT** | Abclonal | Cat#AP0637  WB(1:800) |
| **Rabbit anti-p-AKT** | Abclonal | Cat#A18675  WB(1:800) |
| **Rabbit anti-LXRα** | Abclonal | Cat#A3974  IF(1:100) |
| **Mouse anti-Tuj1** | Abcam | Cat#ab78078  IF(1:400) |
| **Rabbit anti-mTOR** | Abclonal | Cat#A25581  WB(1:800) |
| **Rabbit anti-p-mTOR** | Abclonal | Cat#AP1413  WB(1:800) |
| **Rabbit anti-iNOS** | Abclonal | Cat#A3774  WB(1:800) |
| **Rabbit anti-Arg1** | Proteintech | Cat#16001-1-AP  WB(1:800) |
| **Goat anti-mouse Alexa fluor 488** | Invitrogen | Cat#A-11001  IF(1:400) |
| **Goat anti-mouse Alexa fluor 568** | Invitrogen | Cat#A-11031  IF(1:400) |
| **Goat anti-rabbit Alexa Fluor 488** | Invitrogen | Cat#A-11008  IF(1:400) |
| **Goat anti-rabbit Alexa Fluor 568** | Invitrogen | Cat#A-11011  IF(1:400) |
| **Goat anti-rat Alexa Fluor 568** | Invitrogen | Cat#A-11077  IF(1:400) |
| **Goat anti-rat Alexa Fluor 488** | Invitrogen | Cat#A-11006  IF(1:400) |
| **Goat anti-rabbit IgG** | Invitrogen | Cat#31460  WB(1:3000) |
| **Goat anti-mouse IgG** | Invitrogen | Cat#32430  WB(1:3000) |
| **FluoroMyelin^TM^** | Thermo Fisher  Scientific | Cat#F34652  IF(1:100) |
| **Filipin III** | Sigma-Aldrich | Cat#SAE0087  IF(1:100) |
| **α-BTX** | Sigma-Aldrich | Cat#T0195  IF(1:1000) |
| **Chemicals, peptides, and recombinant proteins** | | |
| **Tamoxifen** | Sigma | Cat#T5648 |
| **Corn oil** | MedChemExpress | Cat#HY-Y1888 |
| **Squalene** | Sigma-Aldrich | Cat#S3626 |
| **Cholesterol** | Sigma-Aldrich | Cat#3045 |
| **MβCD** | Sigma-Aldrich | Cat#C4555 |
| **Forskolin** | Sigma-Aldrich | Cat# F6886 |
| **Heregulin-β1** | Peprotech | Cat# 100–03 |
| **Cytosine arabinoside** | Sigma-Aldrich | Cat#C1768 |
| **dbcAMP** | Sigma-Aldrich | Cat# D0627 |
| **T0901317** | MedChemExpress | Cat#HY-10626 |
| **GSK2033** | AbMole | Cat#1221277-90-2 |
| **Mouse IGF-1 ELISA Kit** | Jianglaishengwu | Cat#JL12620 |
| **Cell Counting Kit-8** | Byotime | Car#C0039 |
| **Oligonucleotides** | | |
| **FDFT1-F:TTACCTTCTTTTAATCCTGGCACCT** | Tsingke | N/A |
| **FDFT1-R:GCAAATGTTTAGAGGGCACTAGG** | Tsingke | N/A |
| **PLP-CreERT2-F:AGGTGGACCTGATCATGGAG** | Tsingke | N/A |
| **PLP-CreERT2-R:ATACCGGAGATCATGCAAGC** | Tsingke | N/A |
| **Dhh-Cre-F:GTCTATCAGTAGTAGGTTCCAGGTTCC** | Tsingke | N/A |
| **Dhh-Cre-R:GAAGCATTTTCCAGGTATGCTCAG** | Tsingke | N/A |
