# Supplementary material for: Schwann Cell Synthesized Cholesterol Orchestrates Peripheral Nerve Regeneration via Structural and IGF1‐Dependent Signaling Mechanisms
Source: Adv Sci (Weinh). 2026 Jan 4;13(16):e20323. doi: 10.1002/advs.202520323 (PMC13042600; doi:10.1002/advs.202520323)

Figure1-E

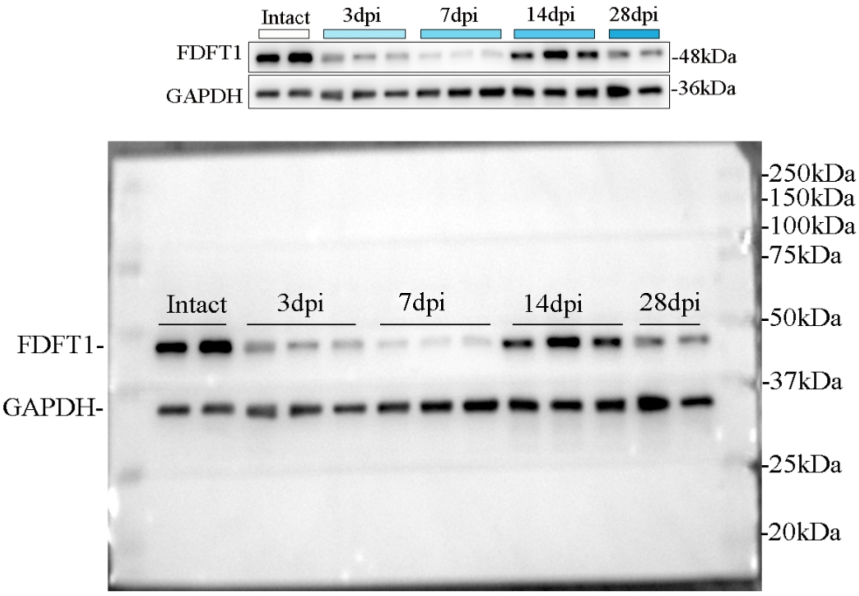

Figure2-F

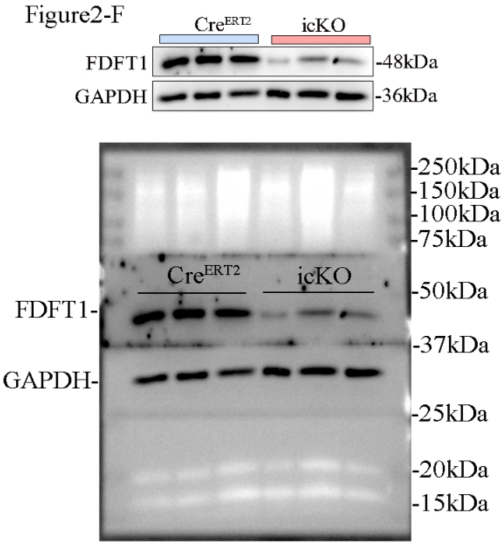

Figure2-H

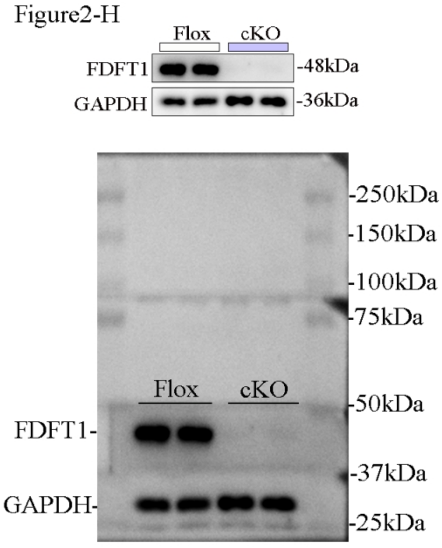

Figure3-C

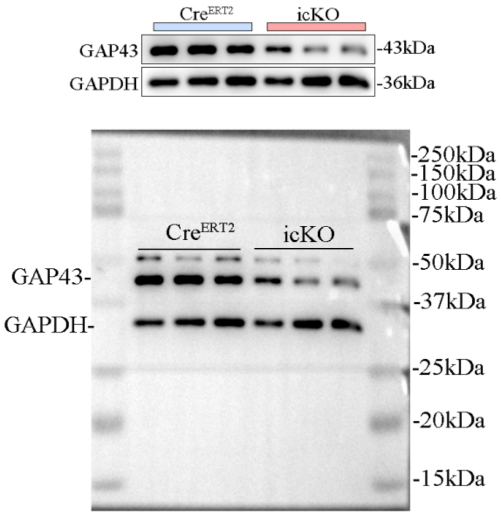

Figure3-E

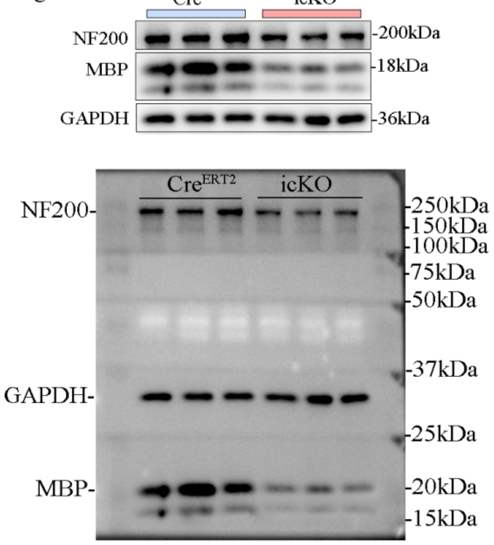

Figure5-B

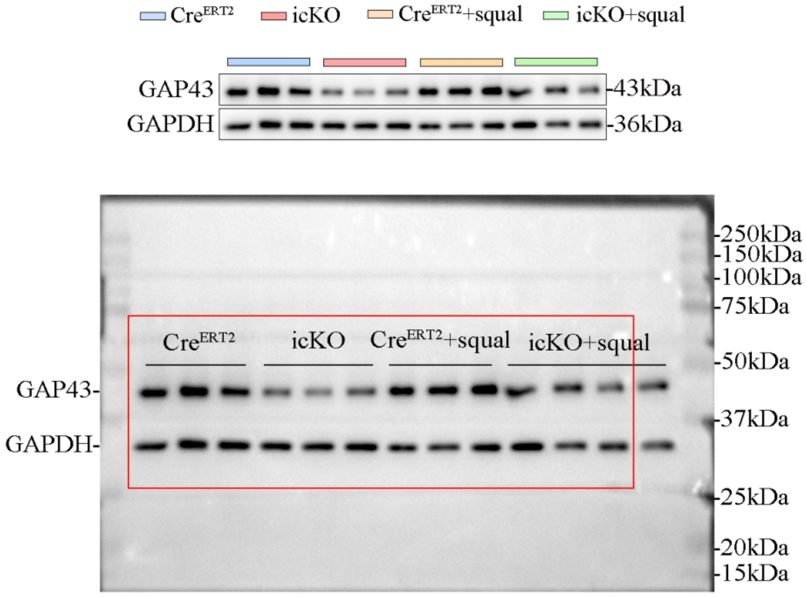

Figure6-E

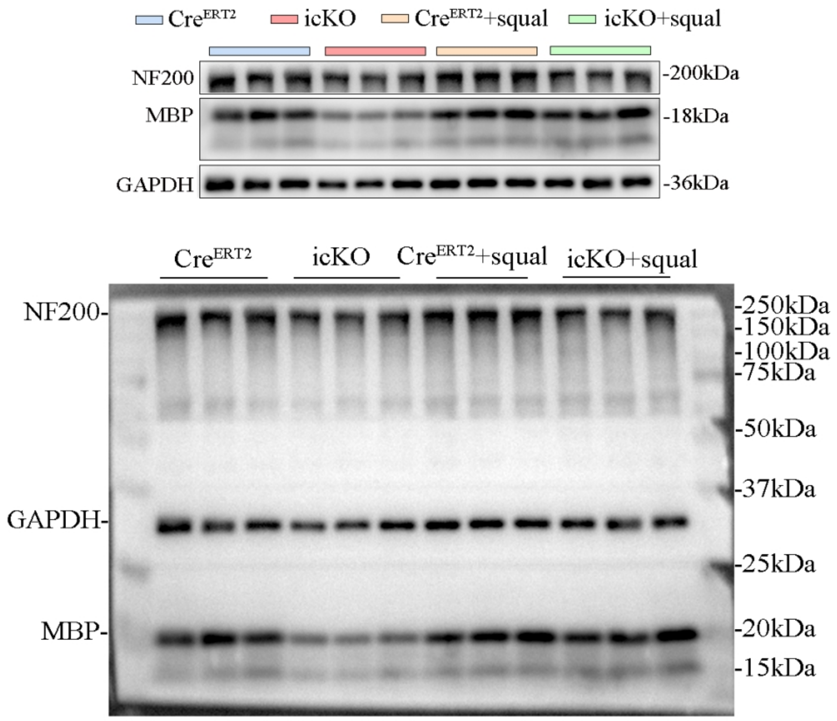

Figure7-B

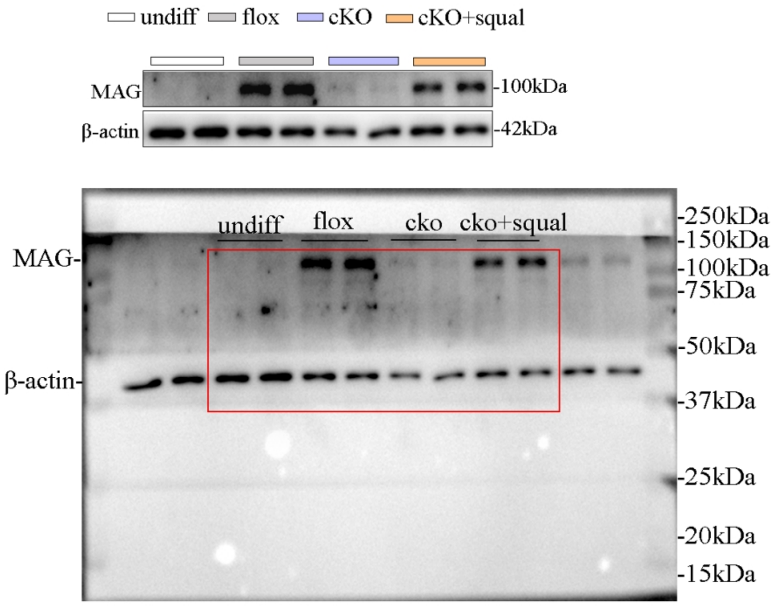

Figure 9-A

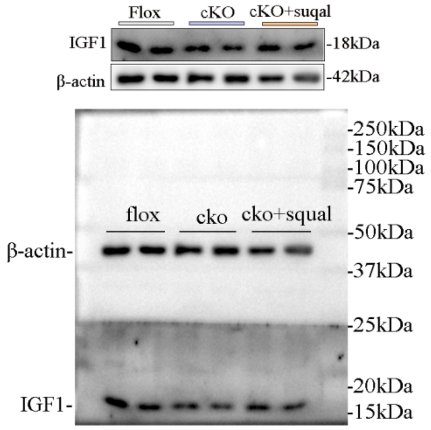

Figure 9-B

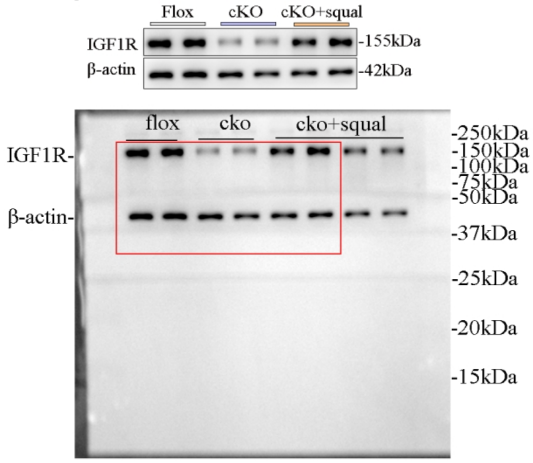

Figure 9-C

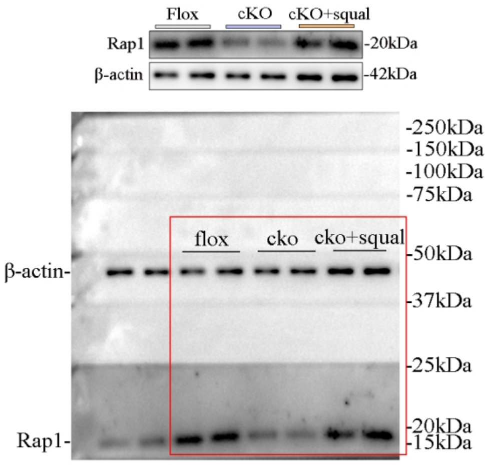

Figure 9-D

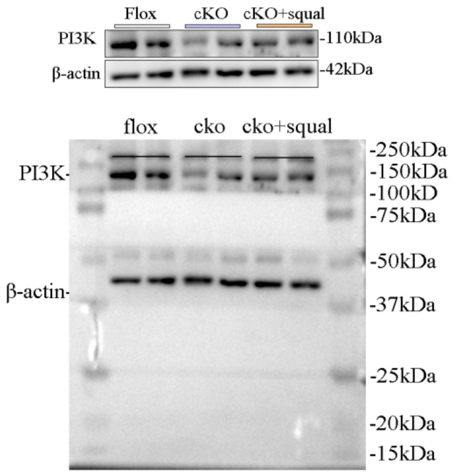

Figure 9-E

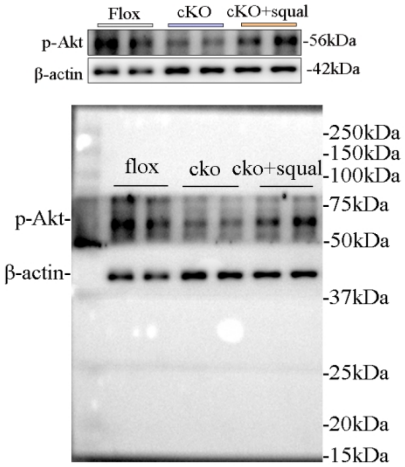

Figure10-E

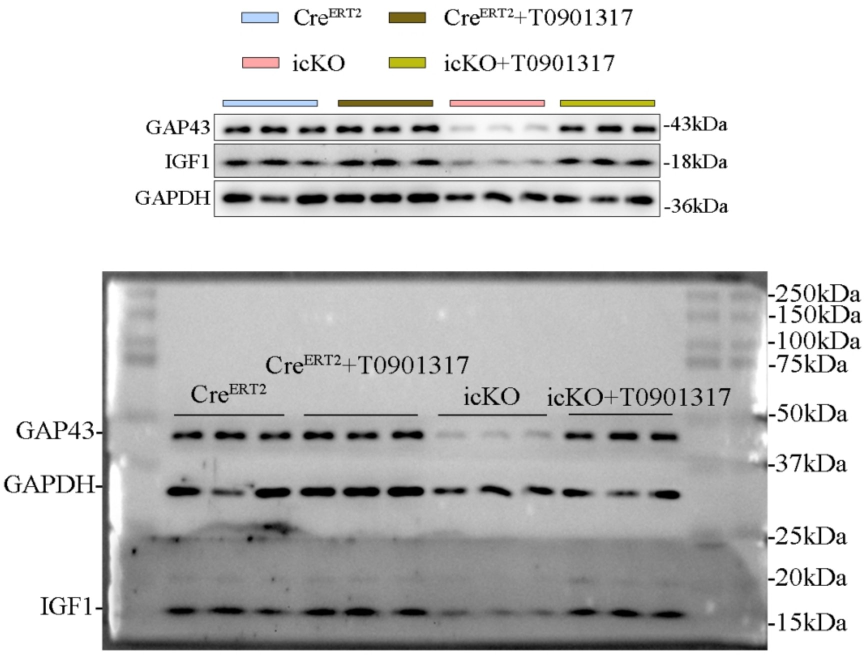

Supplement figure 2-A

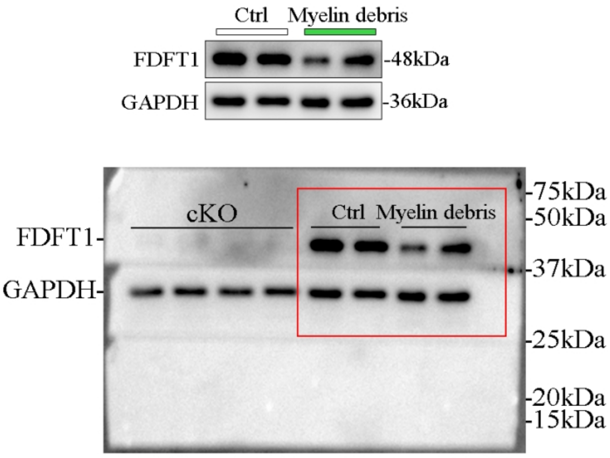

Supplement figure 2-B

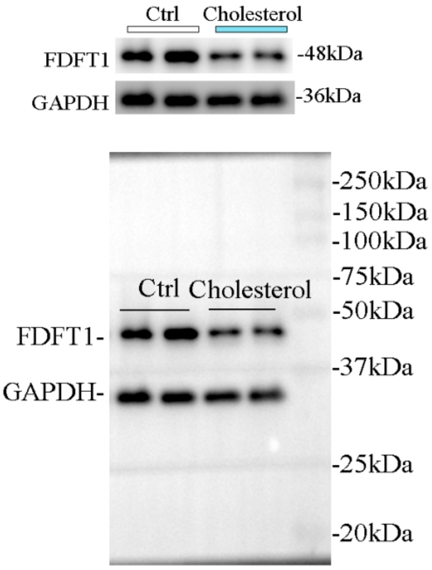

Supplement Figure 3-C

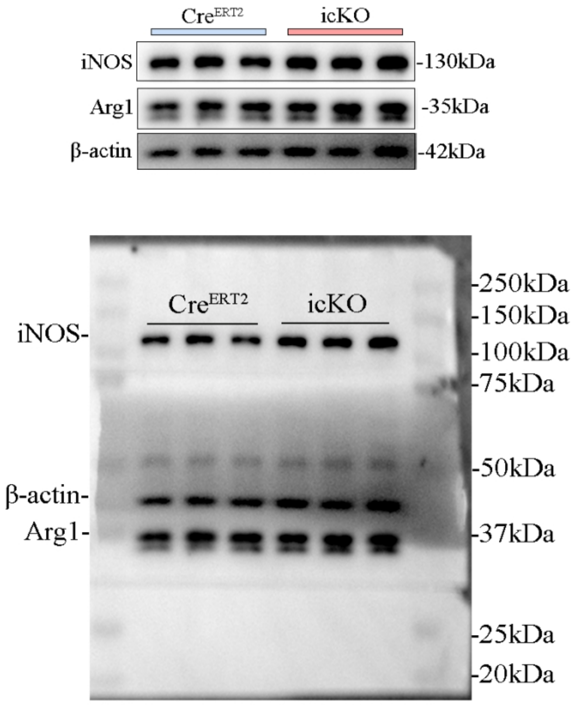

Supplement figure6-A

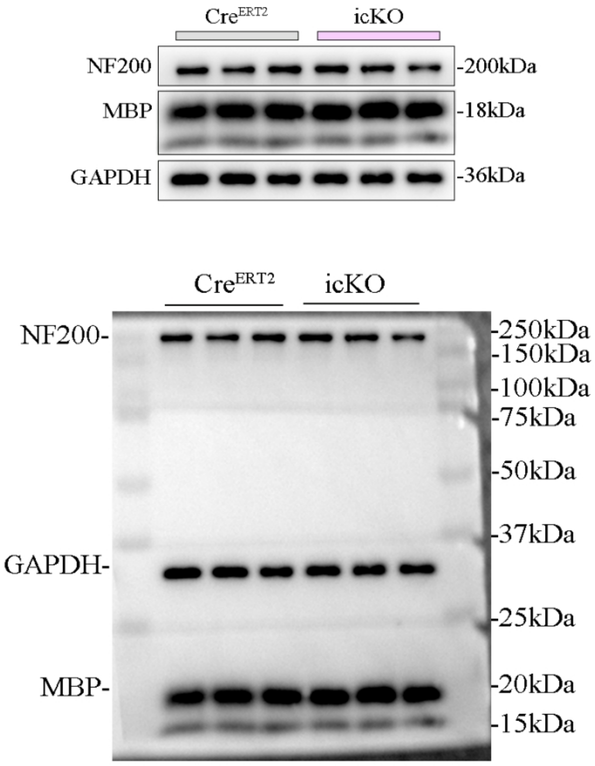

Supplement Figure 11-A

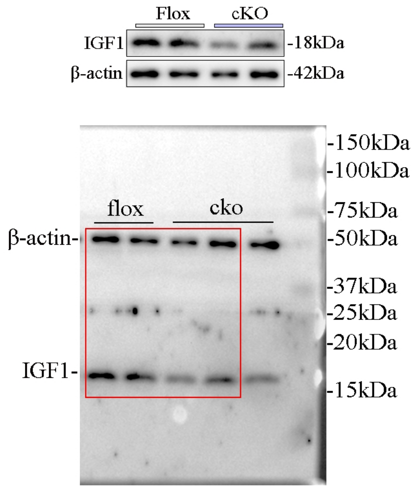

Supplement Figure 11-B

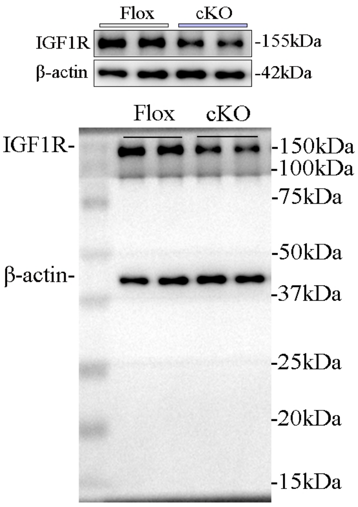

Supplement Figure 11-C

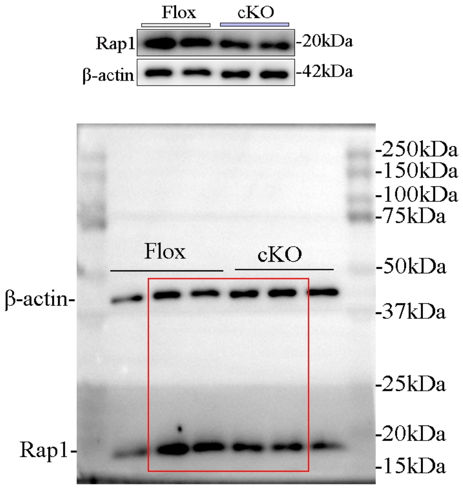

Supplement Figure 11-D

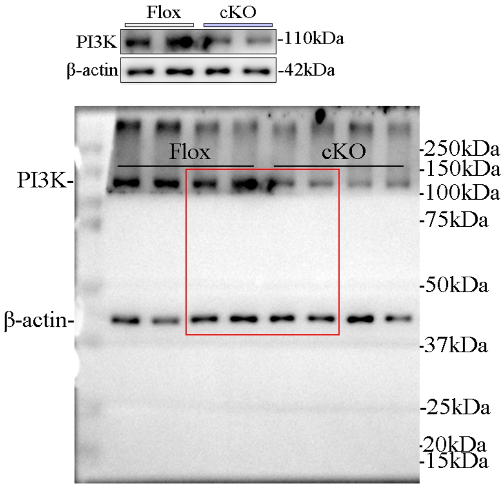

Supplement Figure 11-E

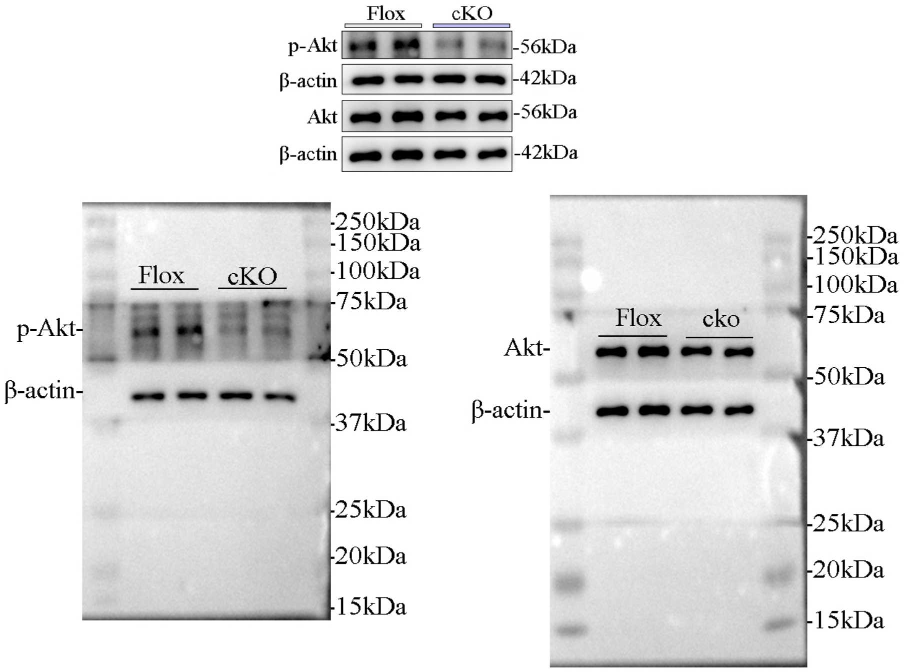

Supplement Figure 11-F

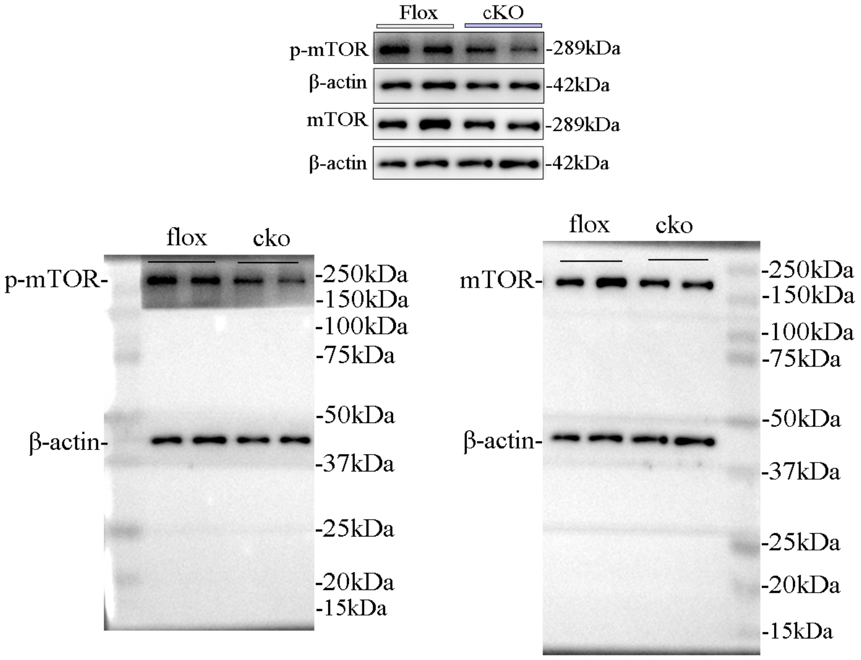

Supplement: Supplementary file 2 — Supporting File 2: advs73654‐sup‐0002‐Data.zip. [file ADVS-13-e20323-s001.zip › RAW DATA of blots.pdf]
