# Supplementary material for: Schwann Cell Synthesized Cholesterol Orchestrates Peripheral Nerve Regeneration via Structural and IGF1‐Dependent Signaling Mechanisms
Source: Adv Sci (Weinh). 2026 Jan 4;13(16):e20323. doi: 10.1002/advs.202520323 (PMC13042600; doi:10.1002/advs.202520323)

Figure 1F

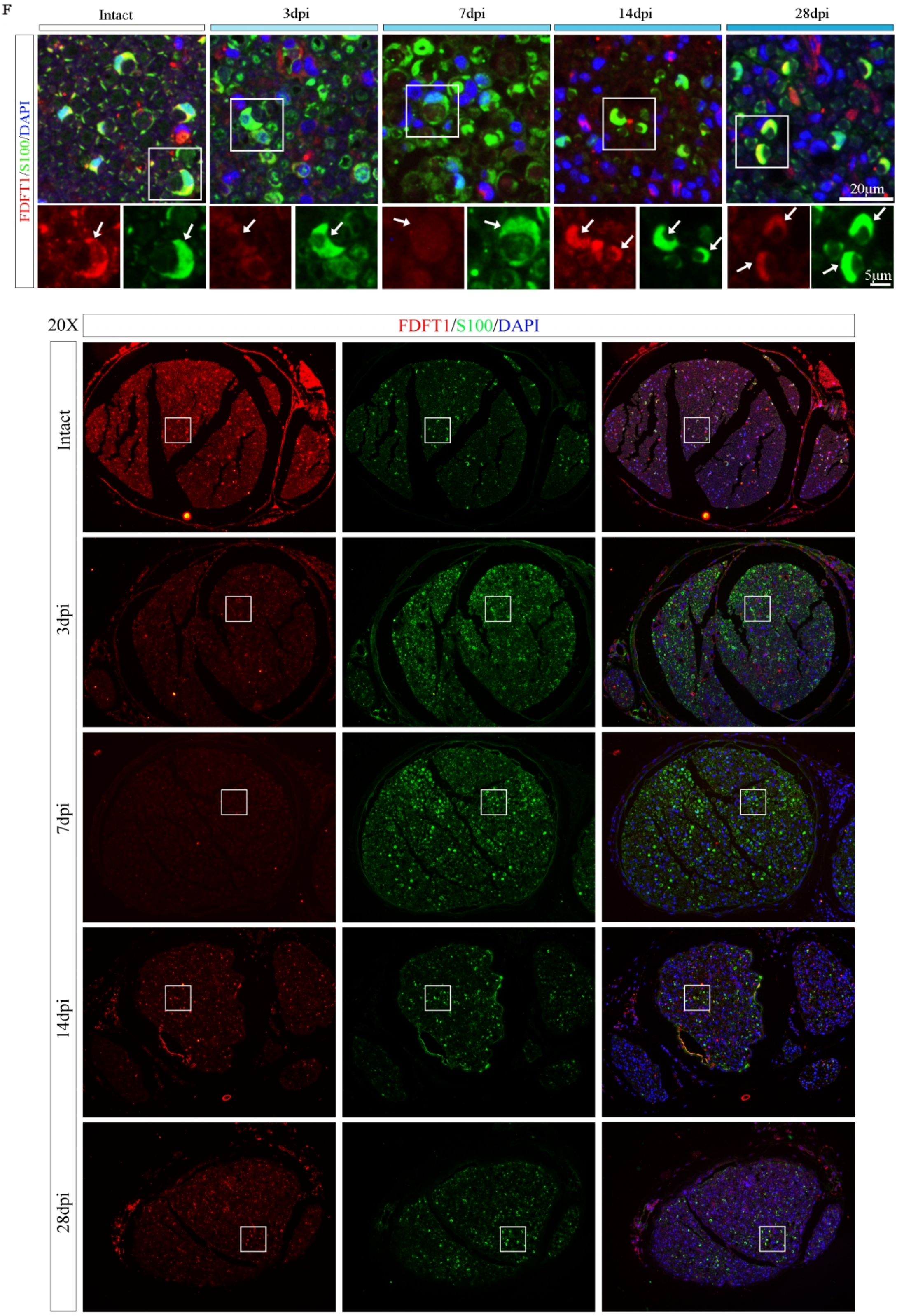

F
Intact
3dpi
7dpi
14dpi
28dpi
FDFT1/S100/DAPI
20μm
5μm
20X
FDFT1/S100/DAPI
Intact
3dpi
7dpi
14dpi
28dpi

Figure 2E

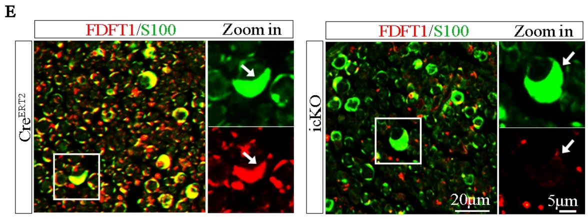

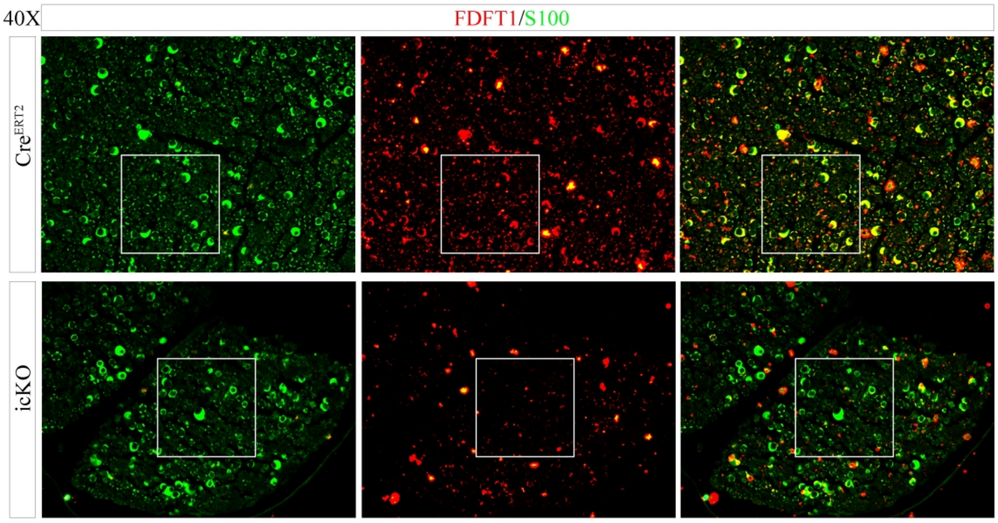

Figure 2G

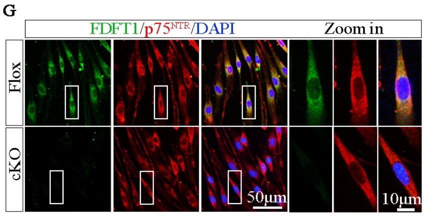

Figure 2G

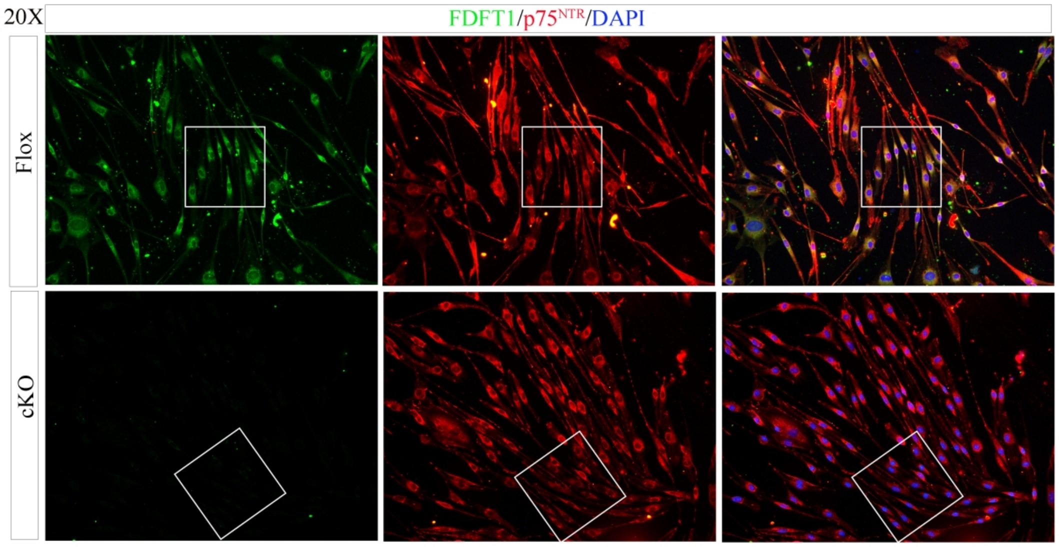

Figure 2I

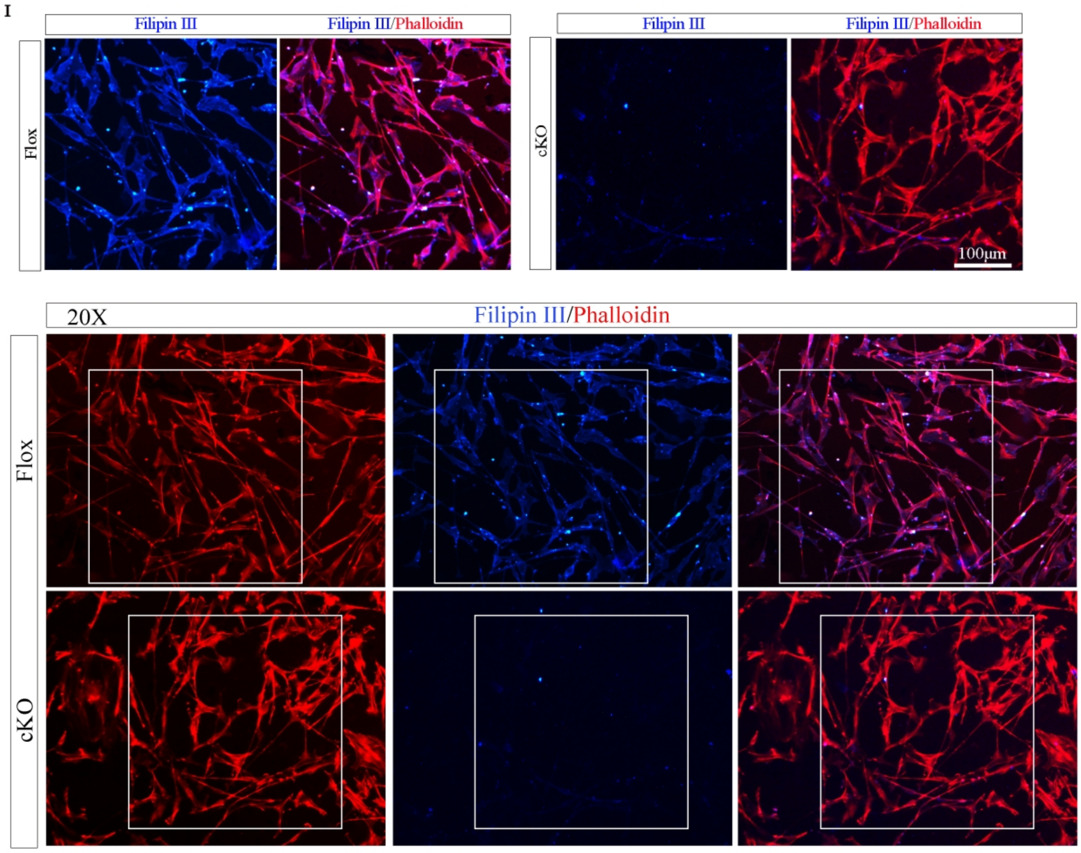

Figure 3A

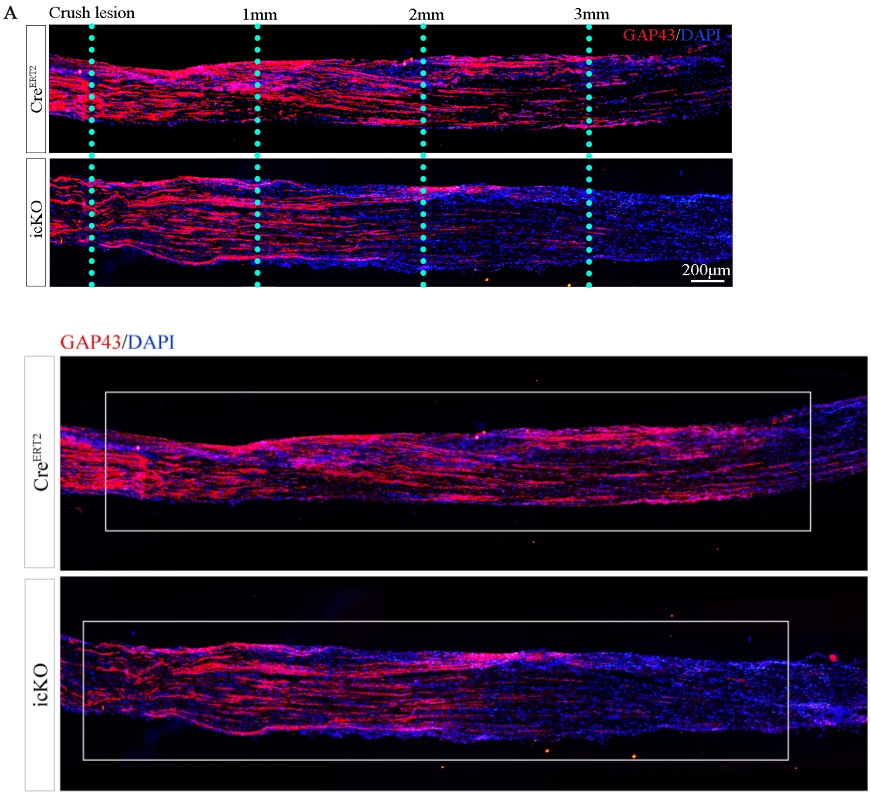

Figure 3B

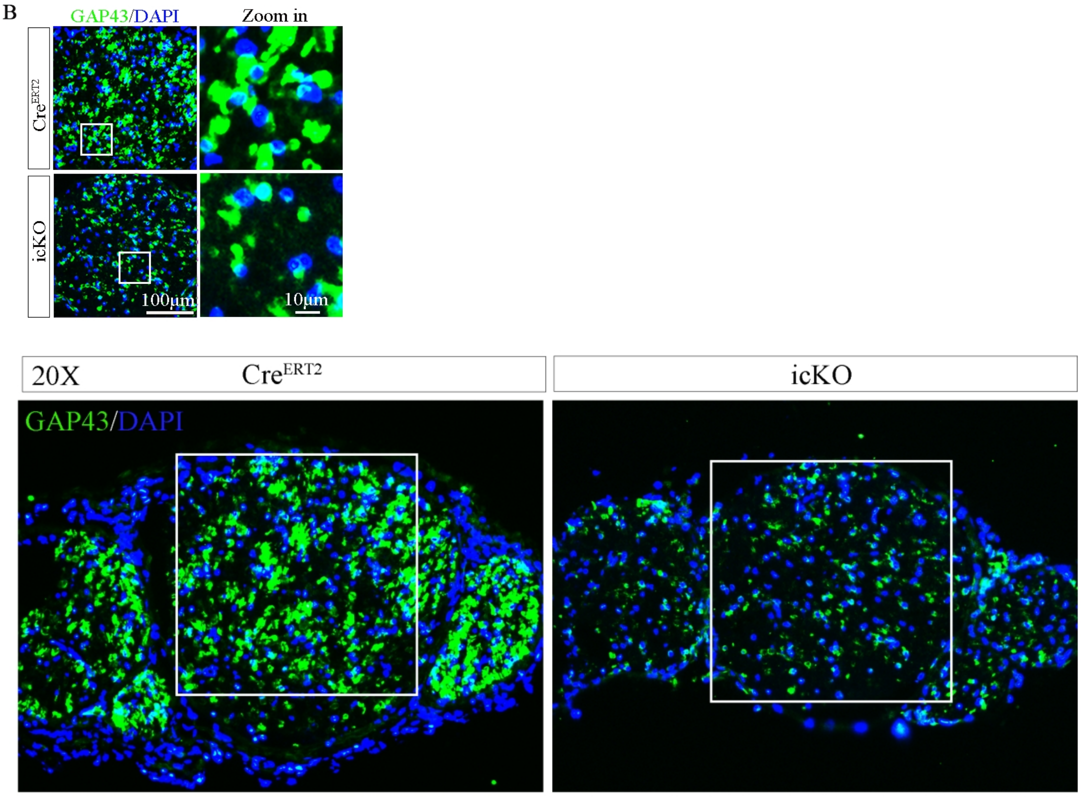

Figure 3D

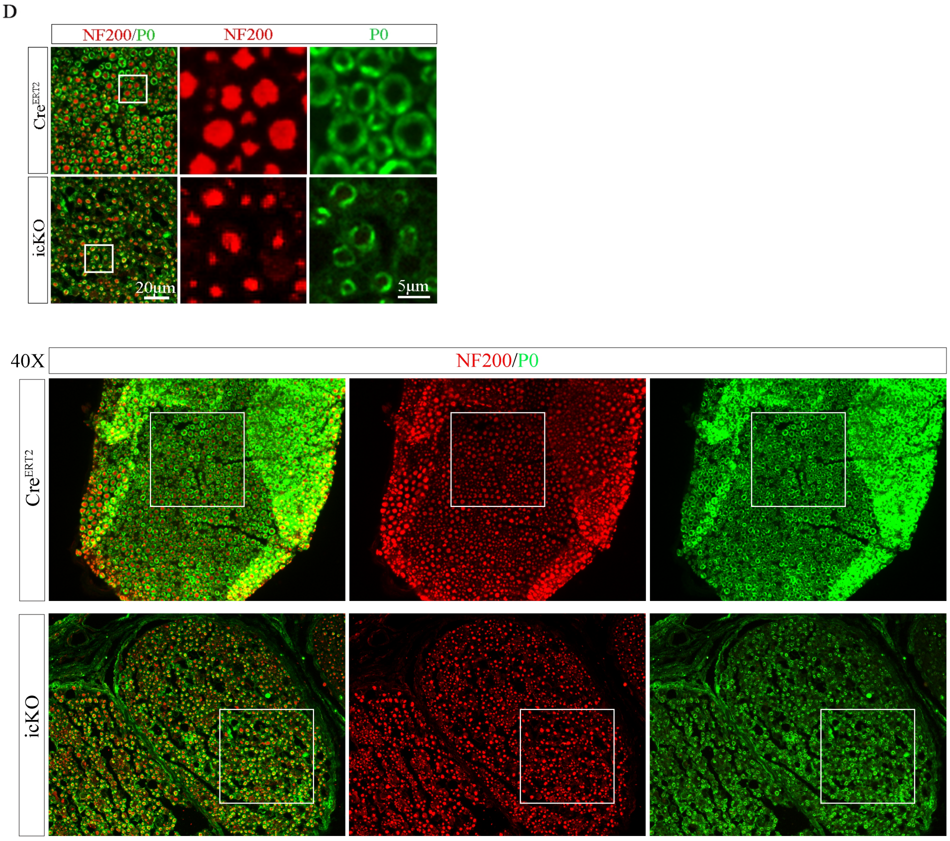

Figure 3F

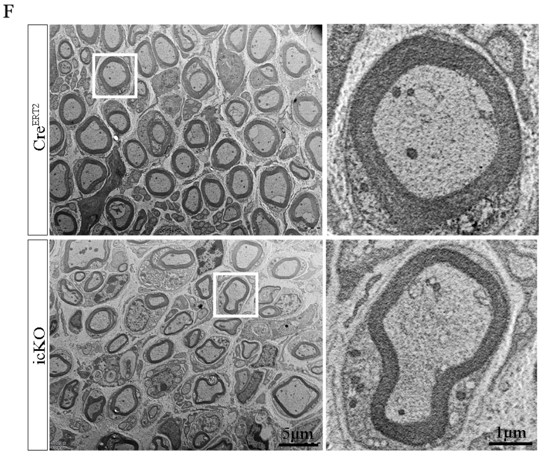

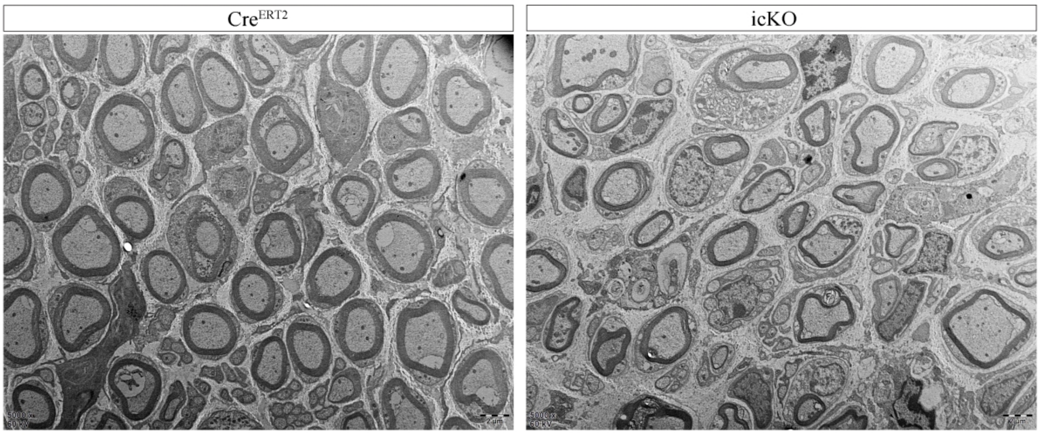

Figure 4G

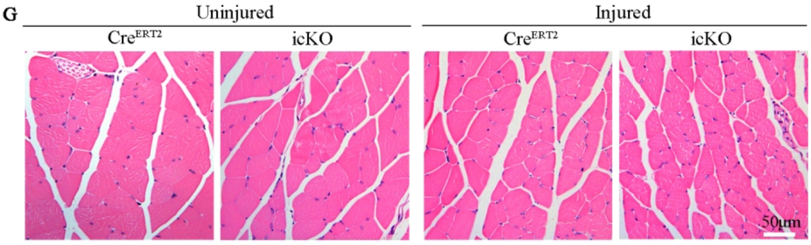

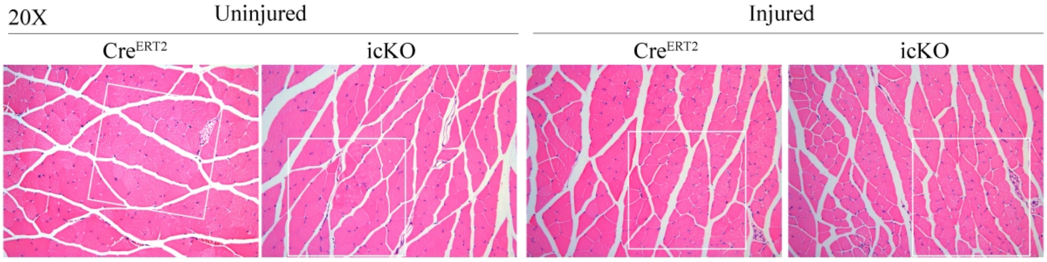

Figure 5A

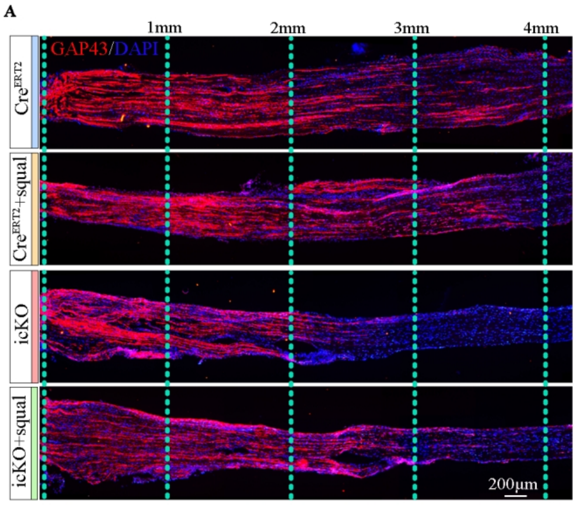

A
1mm
2mm
3mm
4mm
GAP43/DAPI
Cre^ERT2
Cre^ERT2+squal
icKO
icKO+squal
200μm

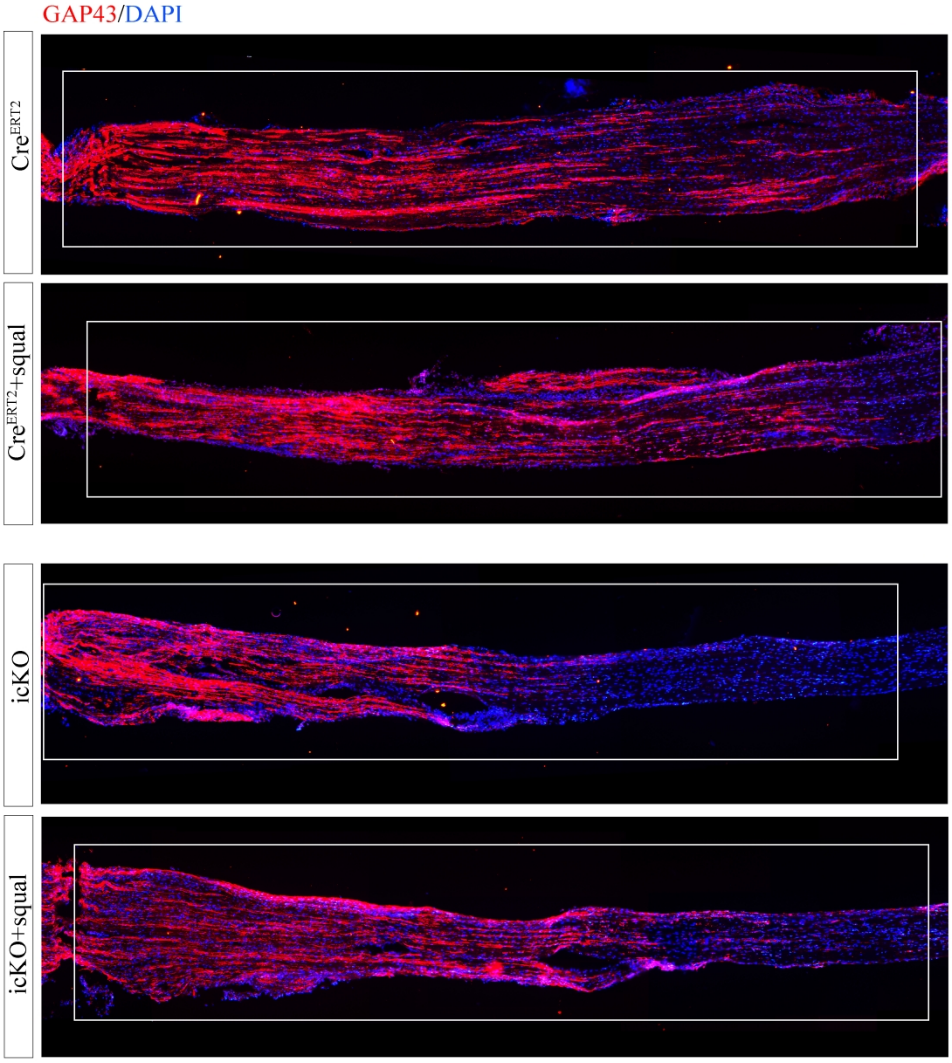

GAP43/DAPI
Cre^ERT2
Cre^ERT2+squal
icKO
icKO+squal

Figure 6D

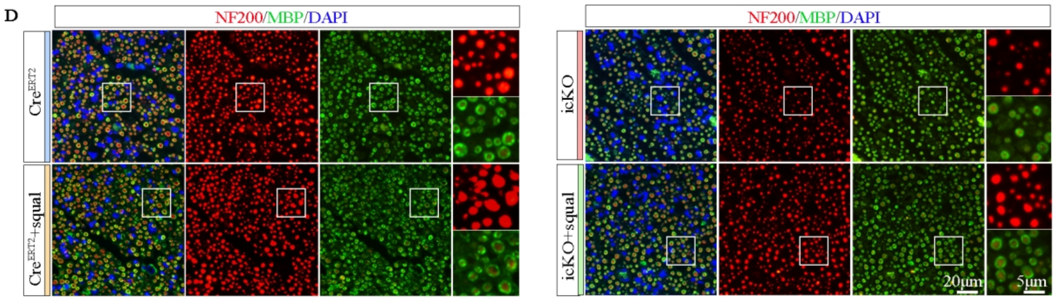
D
NF200/MBP/DAPI
NF200/MBP/DAPI
CreERT2
CreERT2+squal
icKO
icKO+squal
20μm
5μm

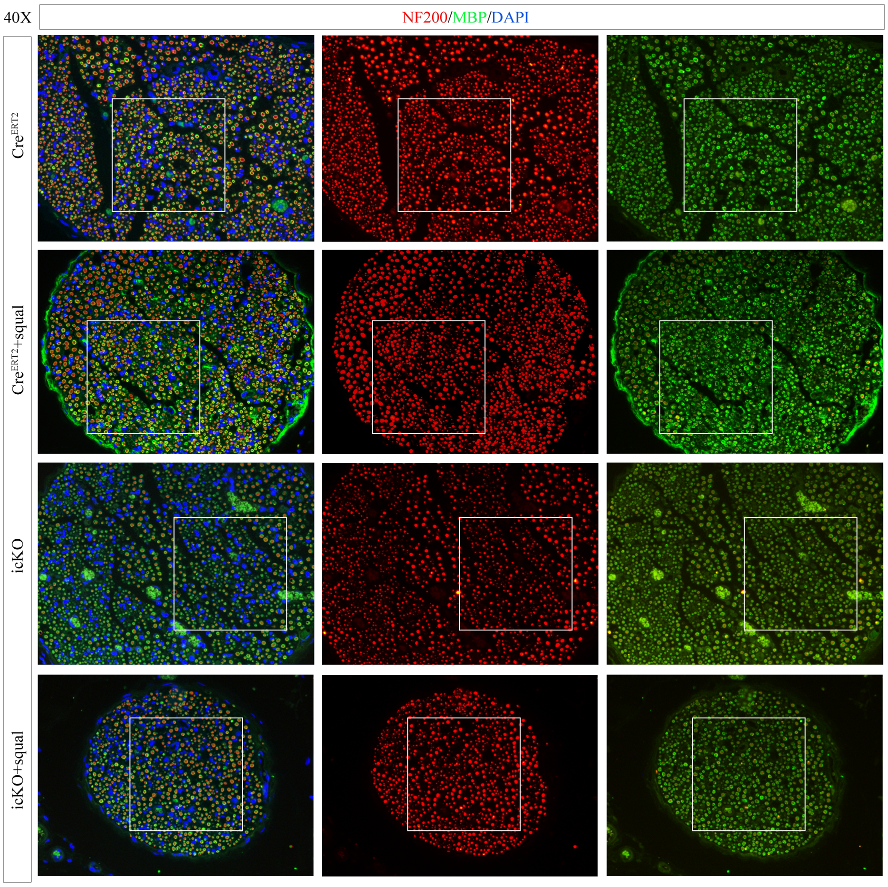
40X
NF200/MBP/DAPI
CreERT2
CreERT2+squal
icKO
icKO+squal

Figure 6F

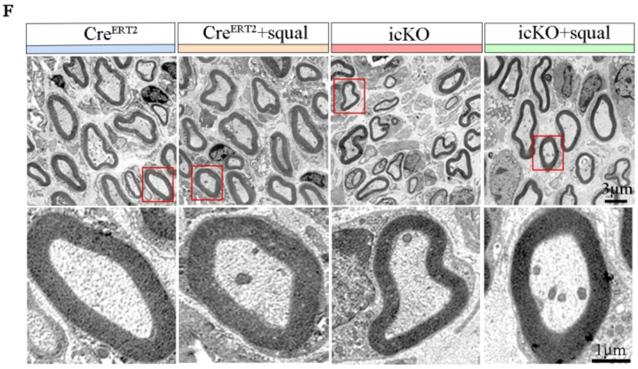
F
Cre^{ERT2}
Cre^{ERT2}+squal
icKO
icKO+squal
3μm
1μm

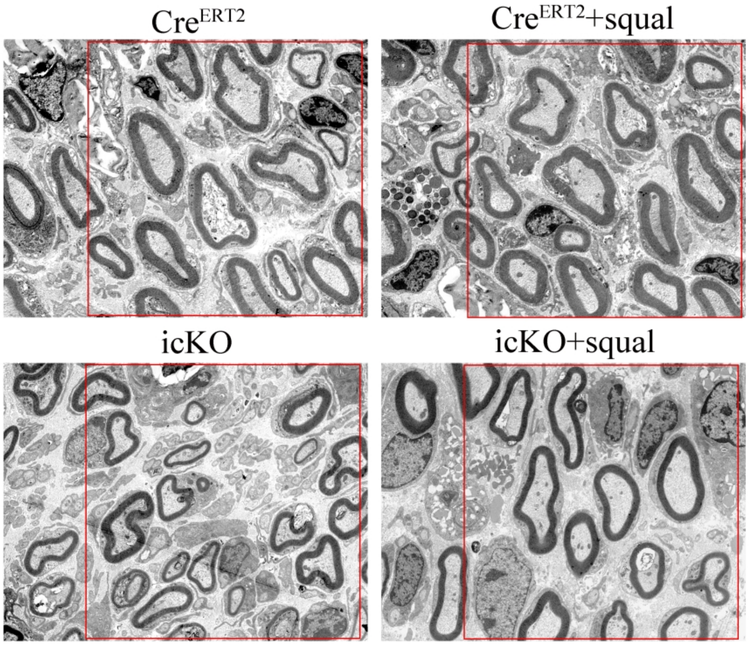
Cre^{ERT2}
Cre^{ERT2}+squal
icKO
icKO+squal

Figure 7C

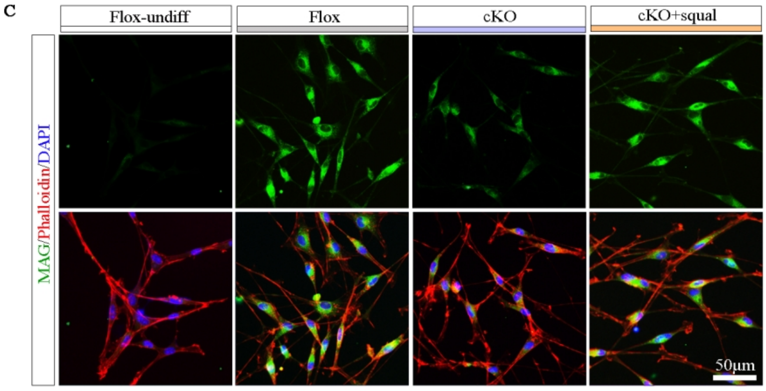

c
Flox-undiff
Flox
cKO
cKO+squal
MAG/Phalloidin/DAPI
50μm

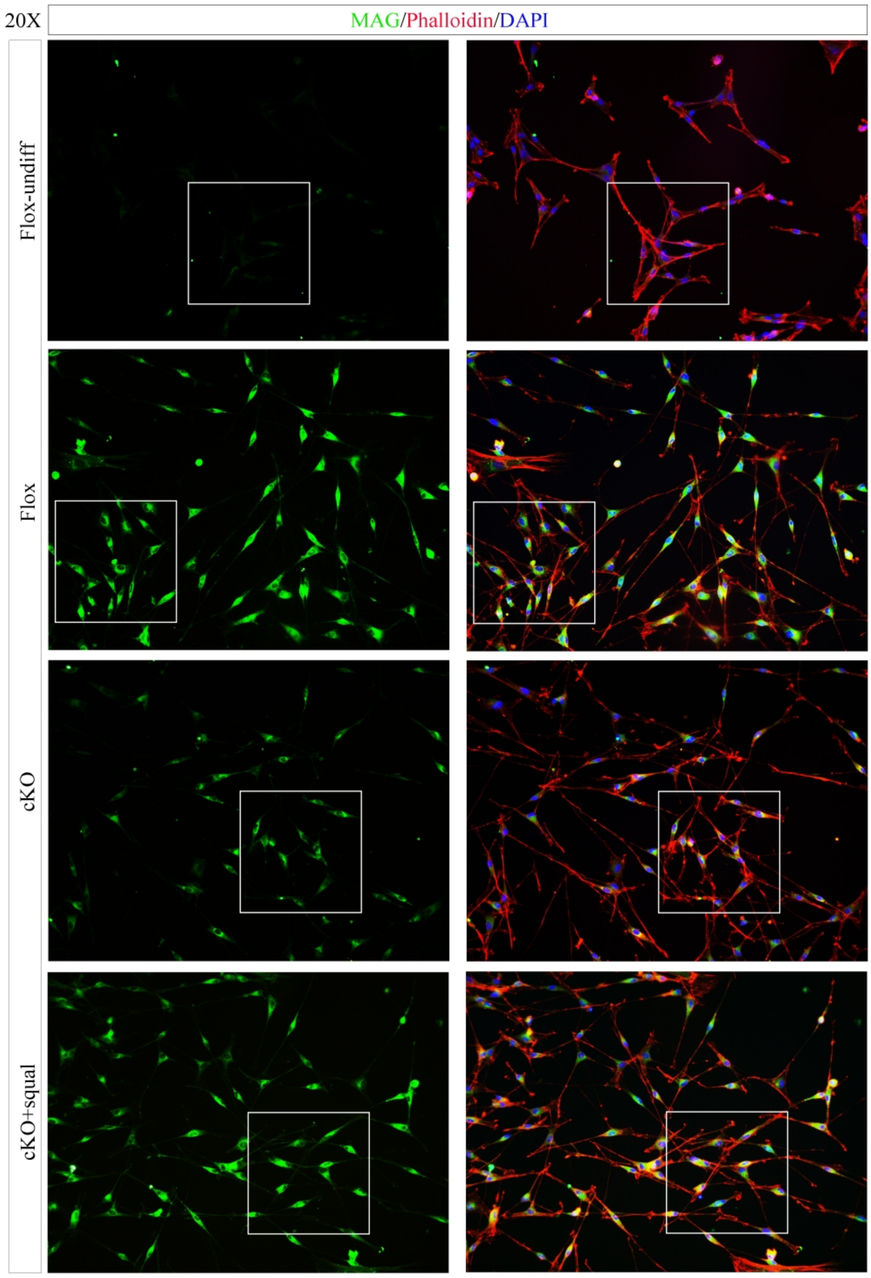

20X
MAG/Phalloidin/DAPI
Flox-undiff
Flox
cKO
cKO+squal

Figure 7D

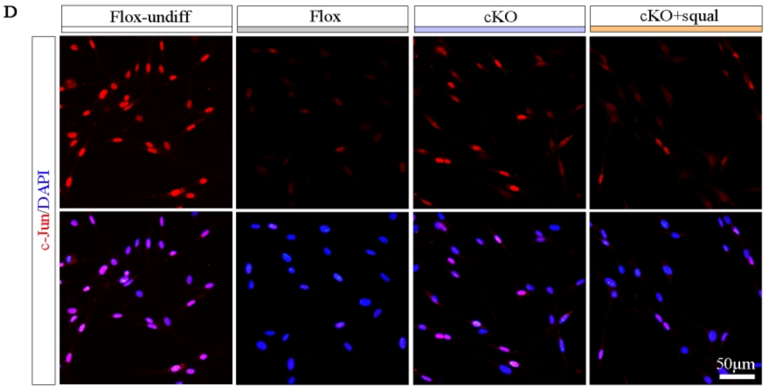
D
Flox-undiff
Flox
cKO
cKO+squal
c-Jun/DAPI
50μm

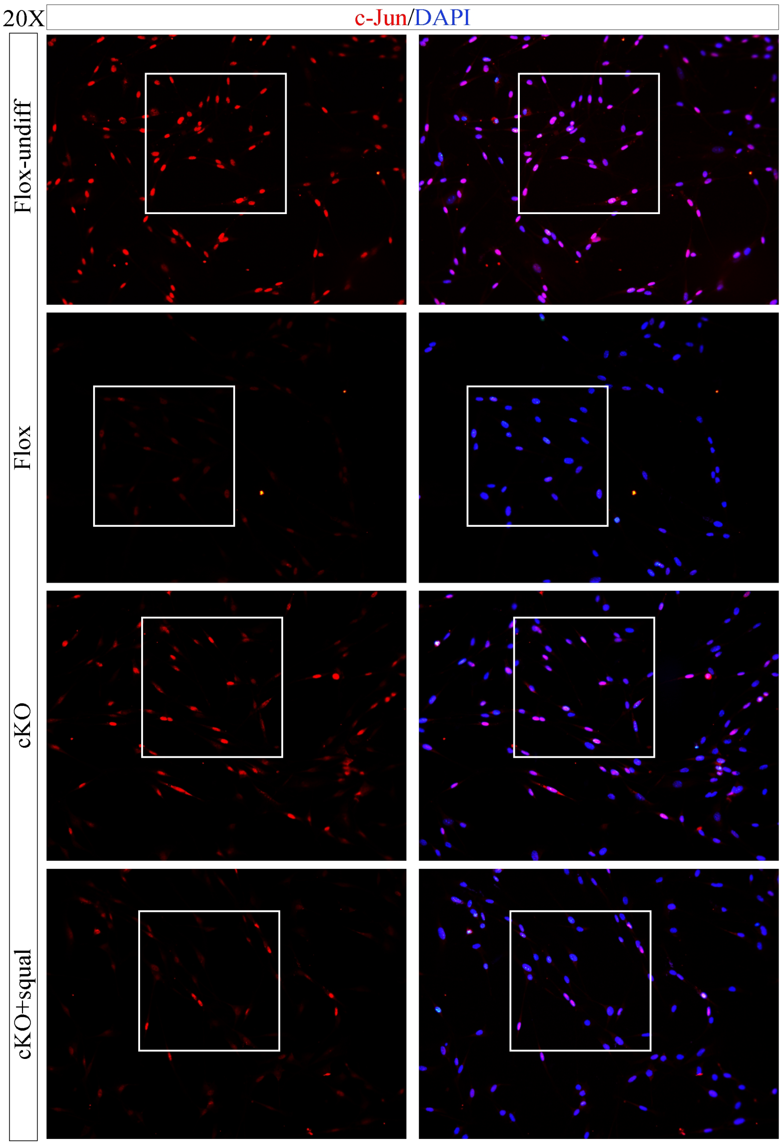
20X
c-Jun/DAPI
Flox-undiff
Flox
cKO
cKO+squal

Figure 9F

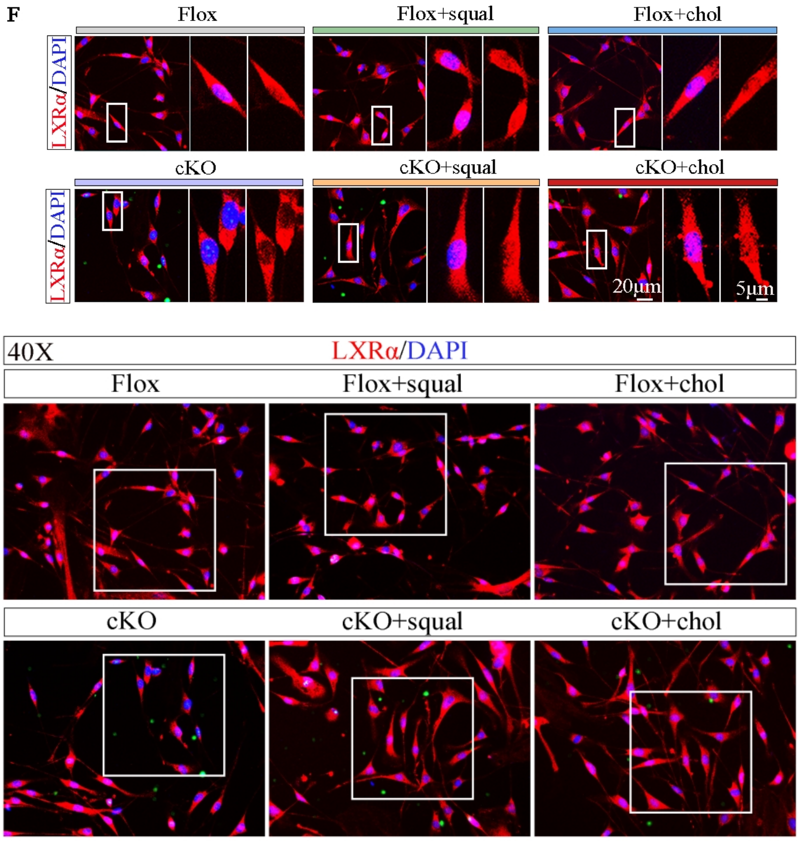

F
Flox
Flox+squal
Flox+chol
LXRα/DAPI
cKO
cKO+squal
cKO+chol
LXRα/DAPI
20μm
5μm
40X
LXRα/DAPI
Flox
Flox+squal
Flox+chol
cKO
cKO+squal
cKO+chol

Figure 9G

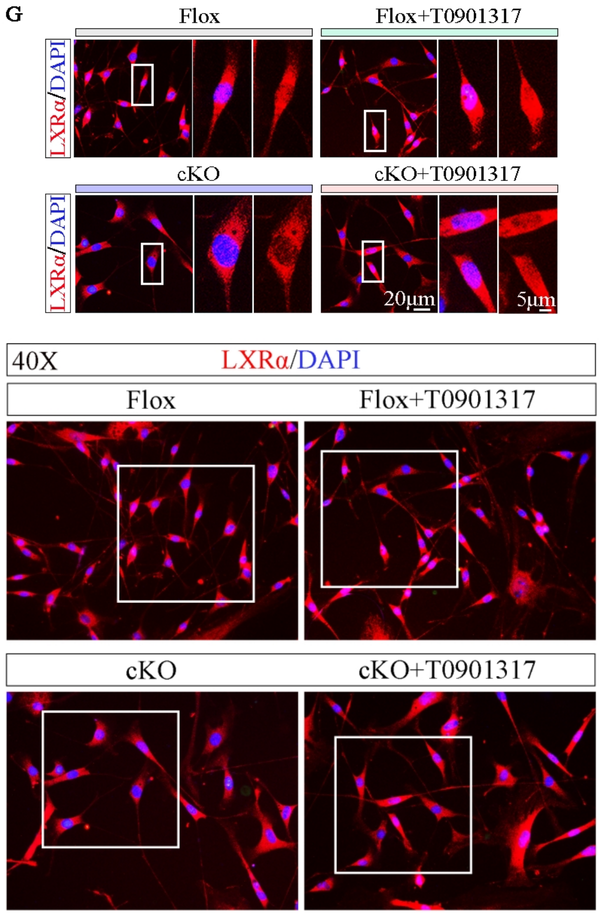
G
Flox
Flox+T0901317
LXRα/DAPI
cKO
cKO+T0901317
LXRα/DAPI
20μm
5μm
40X
LXRα/DAPI
Flox
Flox+T0901317
cKO
cKO+T0901317

Figure 9I

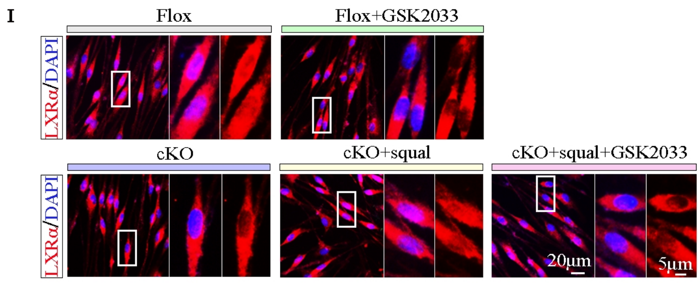
I
Flox
Flox+GSK2033
LXRα/DAPI
cKO
cKO+squal
cKO+squal+GSK2033
LXRα/DAPI
20μm
5μm

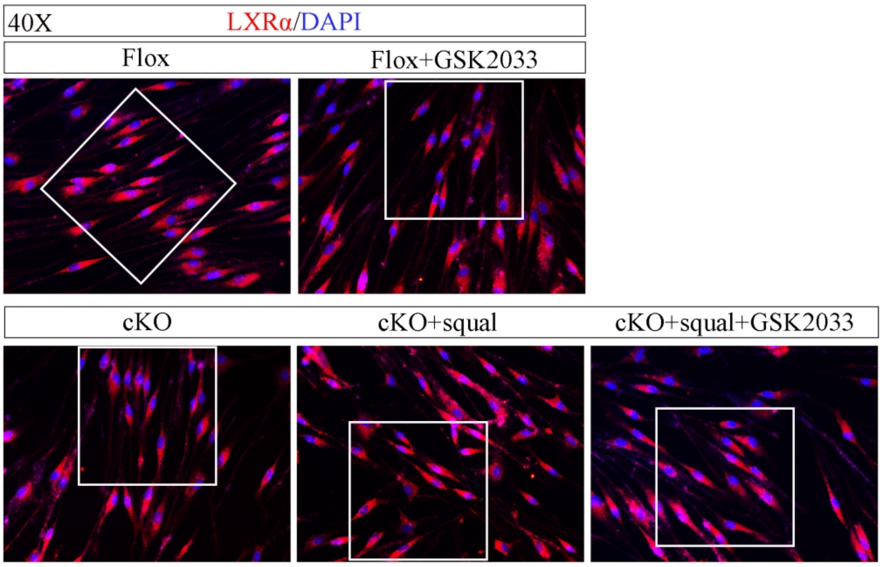
40X
LXRα/DAPI
Flox
Flox+GSK2033
cKO
cKO+squal
cKO+squal+GSK2033

Figure 10C

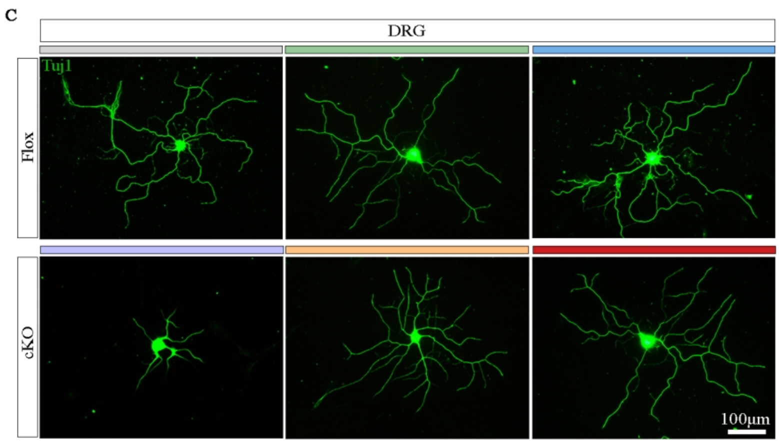
c
DRG
Tuj1
Flox
cKO
100μm

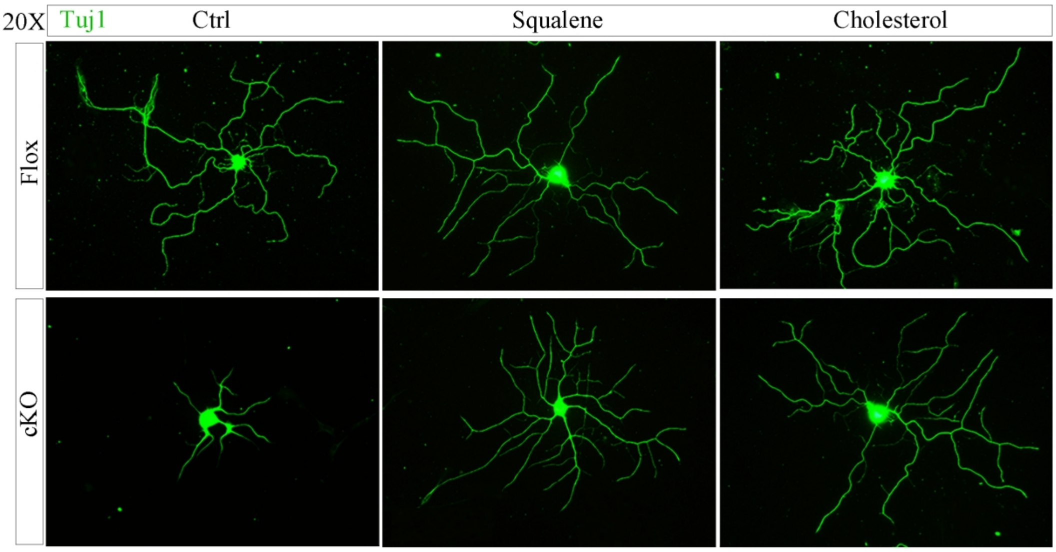
20X
Tuj1
Ctrl
Squalene
Cholesterol
Flox
cKO

Figure 10D

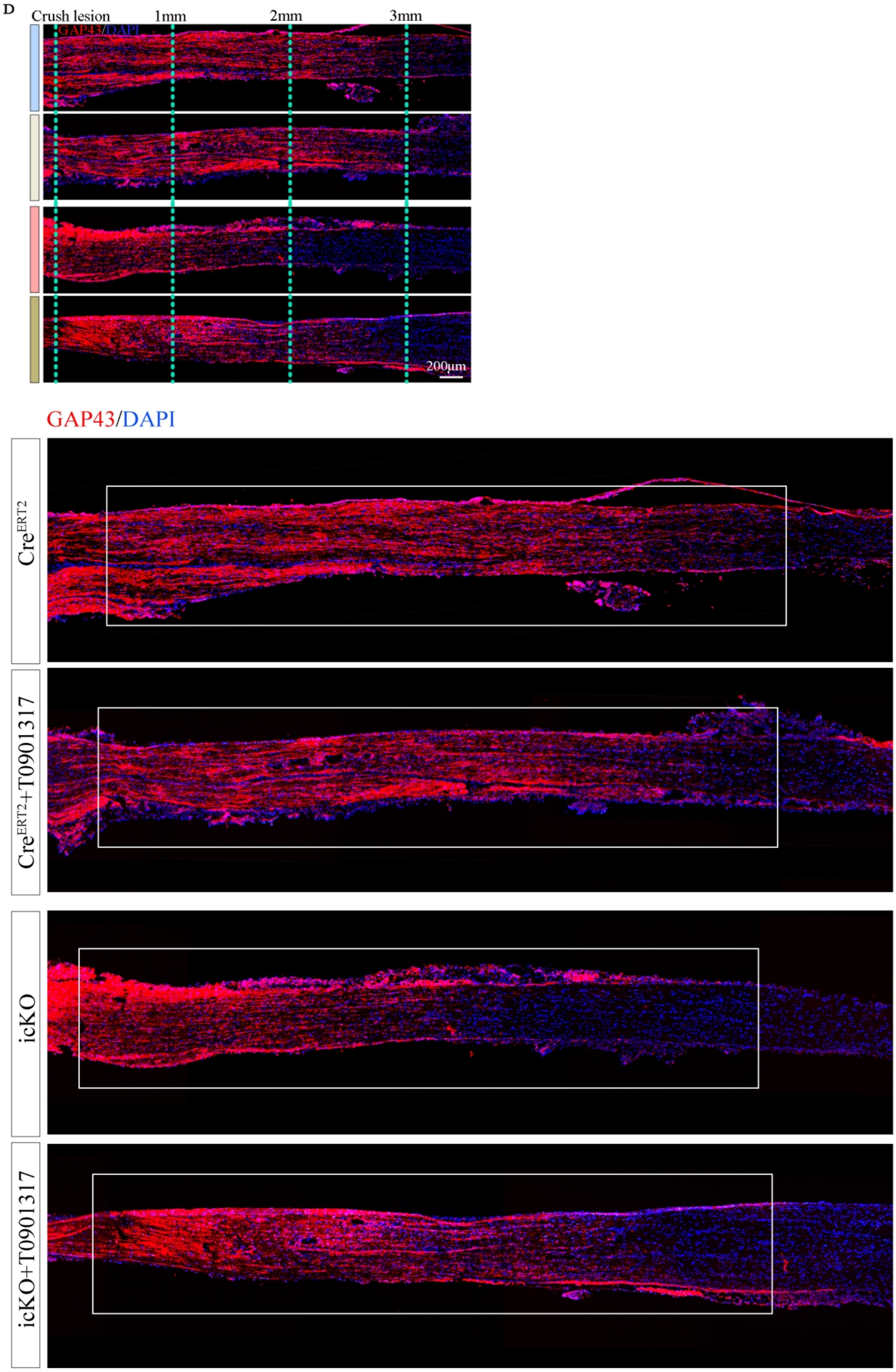
D
Crush lesion
1mm
2mm
3mm
GAP43/DAPI
200μm
GAP43/DAPI
Cre^ERT2
Cre^ERT2+T0901317
icKO
icKO+T0901317

Supplement Figure 1

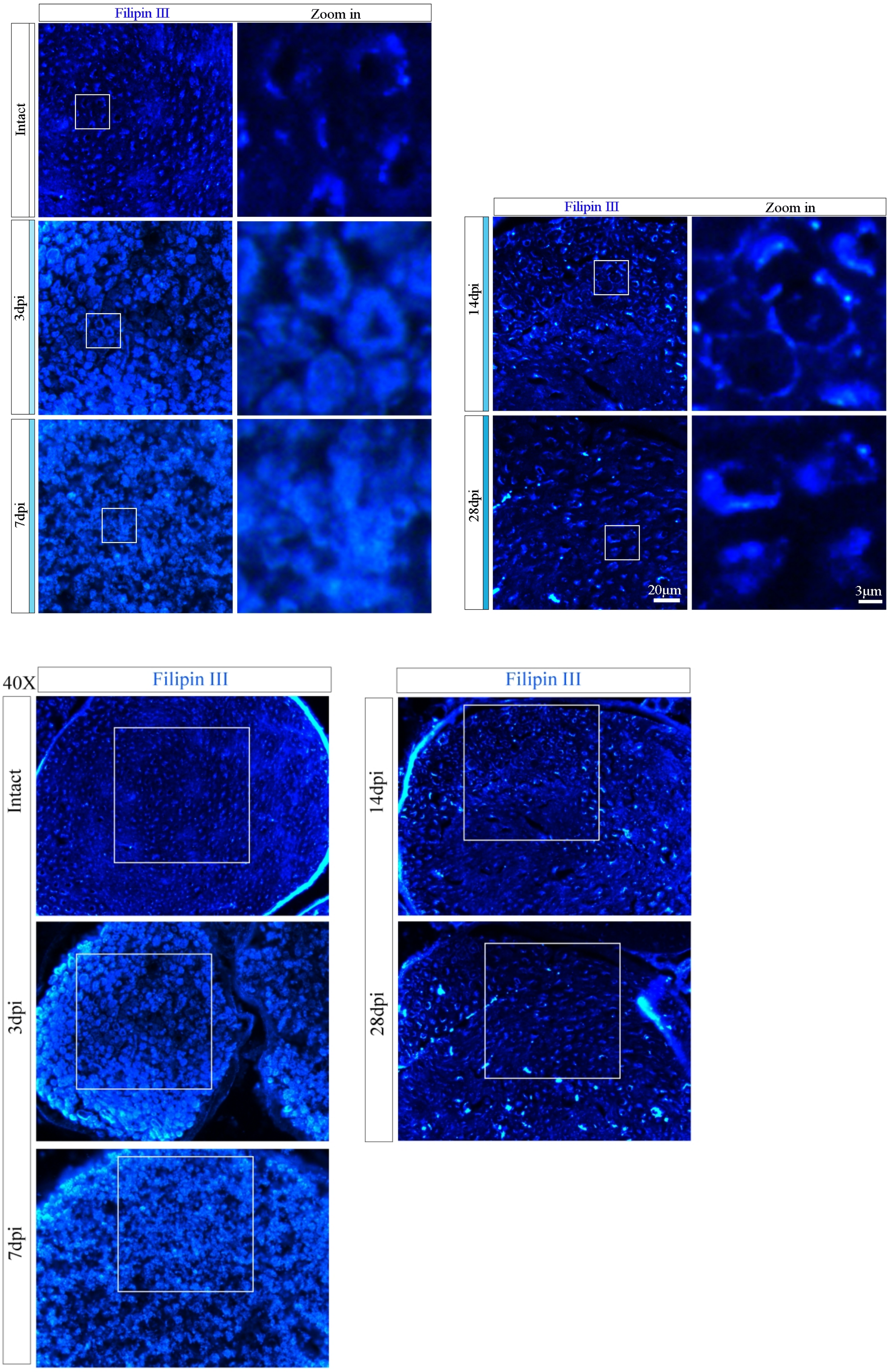

Supplement Figure 3A

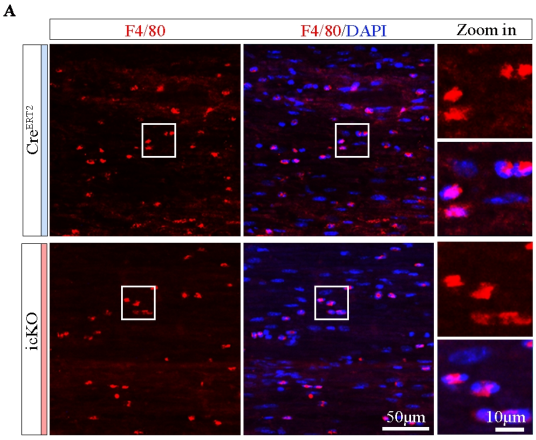

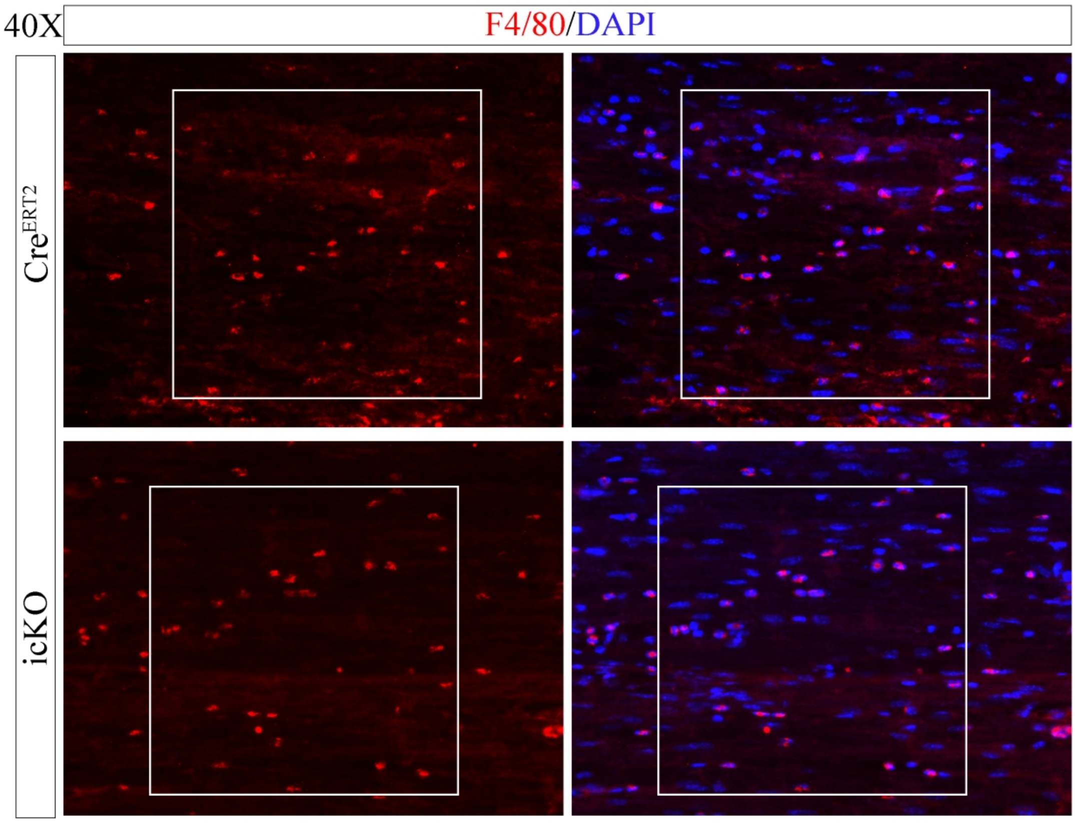

Supplement Figure 3B

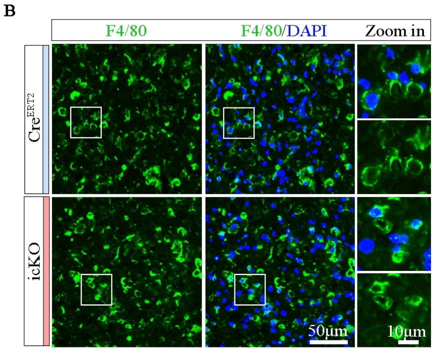

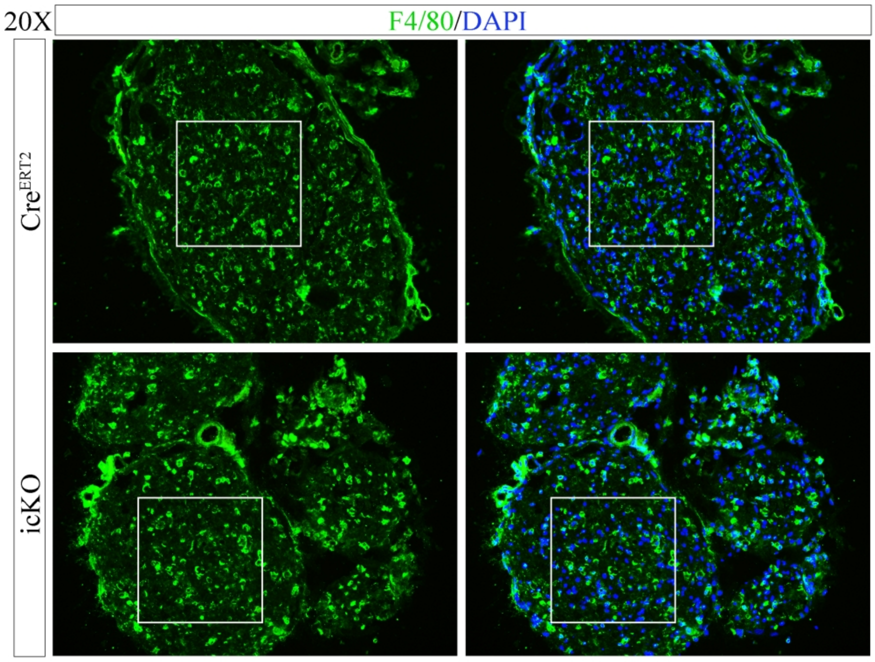

Supplement Figure 5

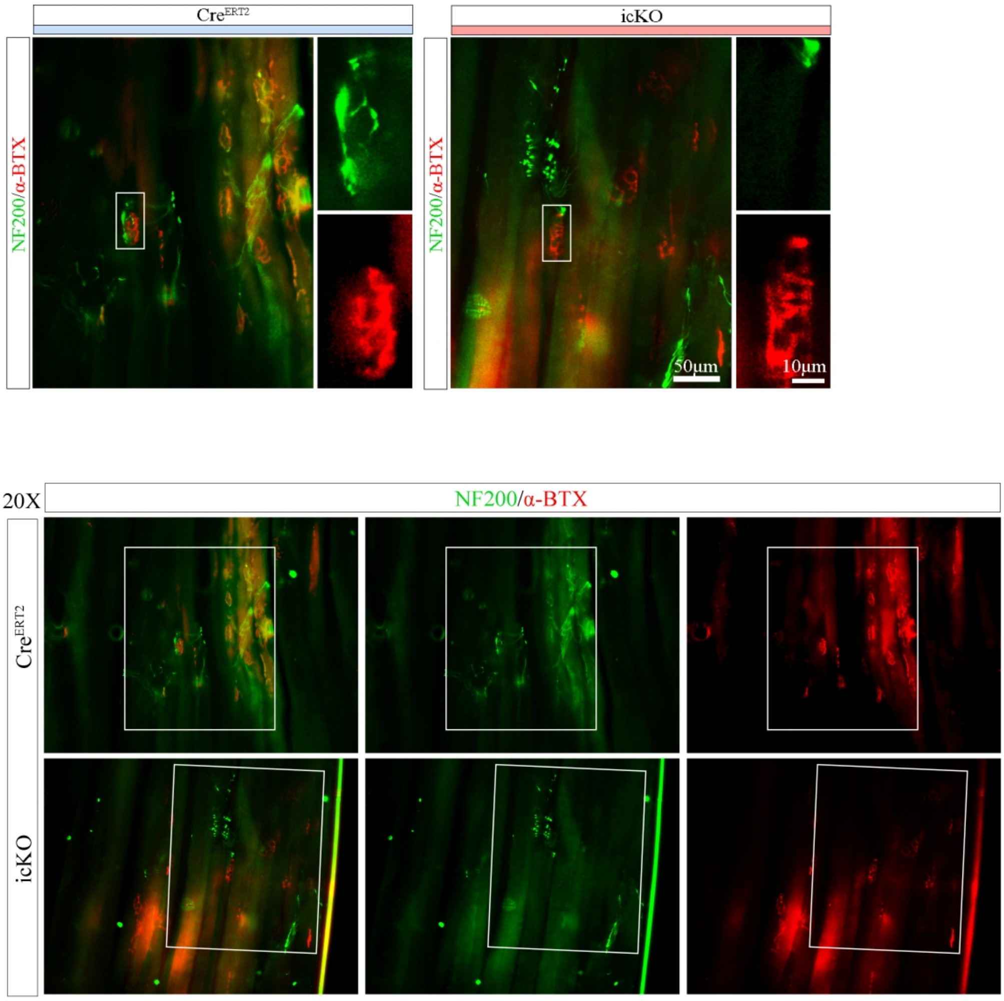

Supplement Figure 6B

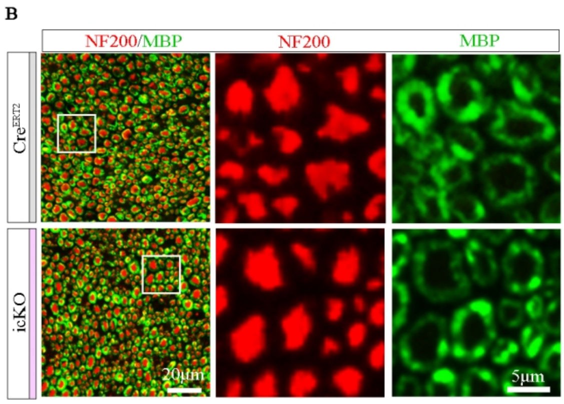

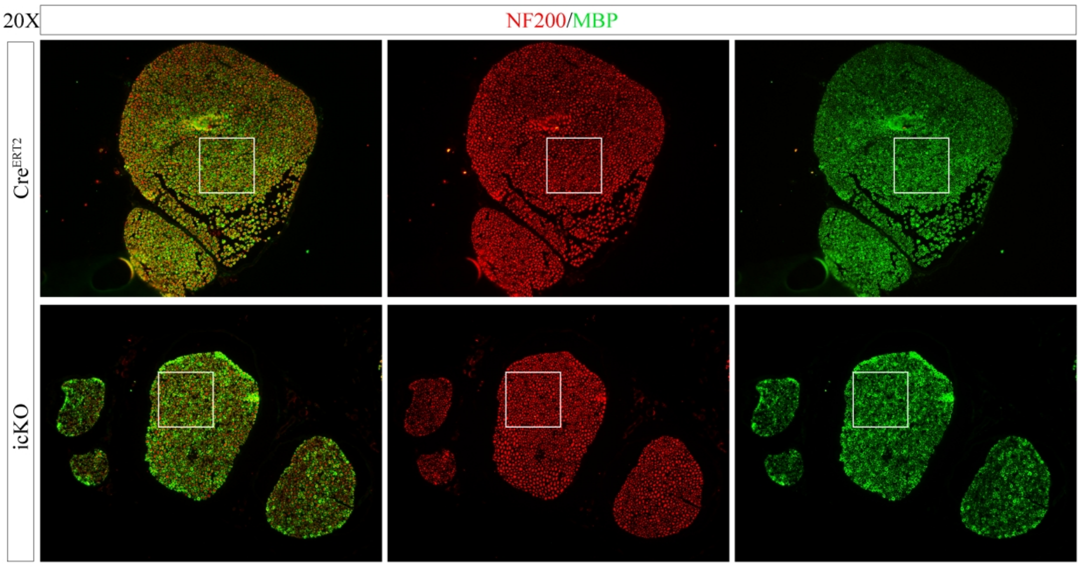

Supplement Figure 7

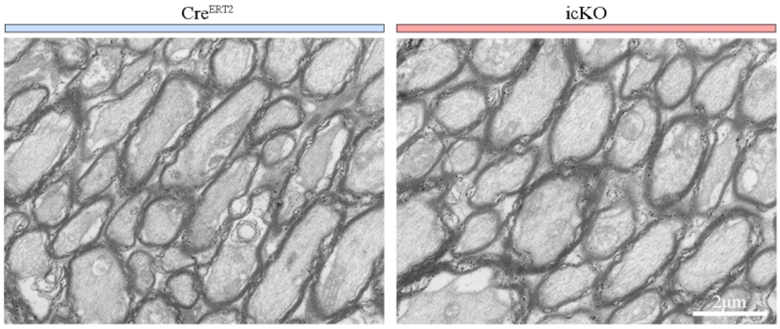

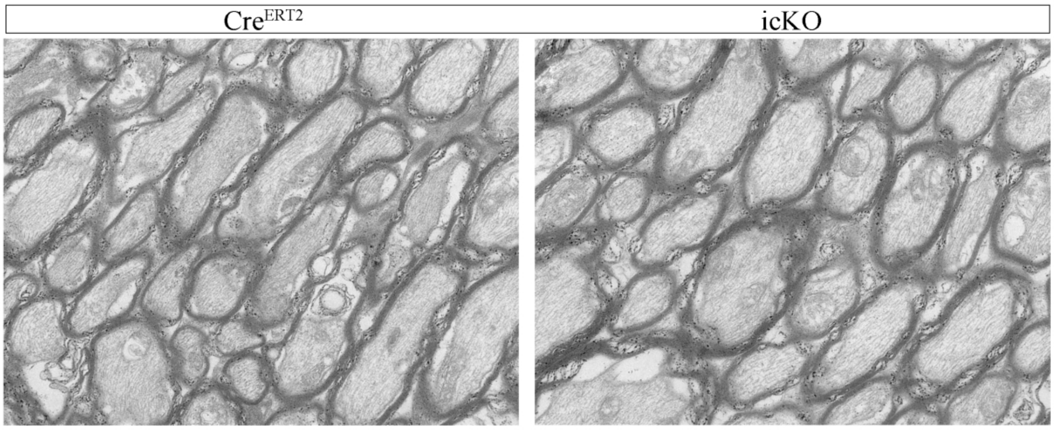

Supplement Figure 8E

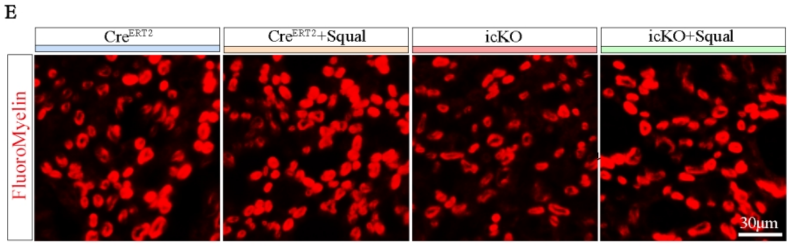

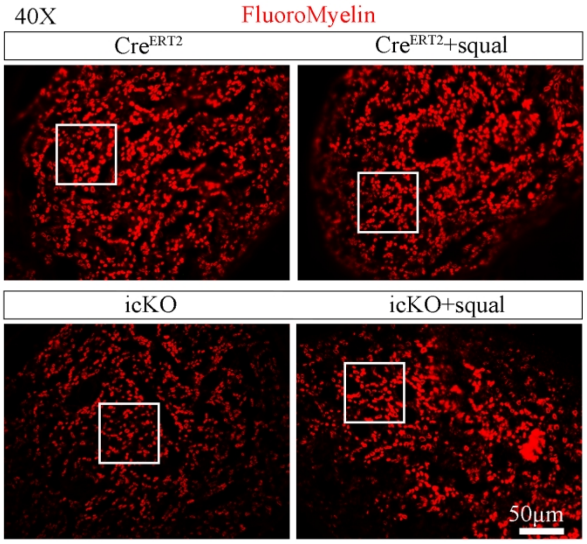

Supplement Figure 8H

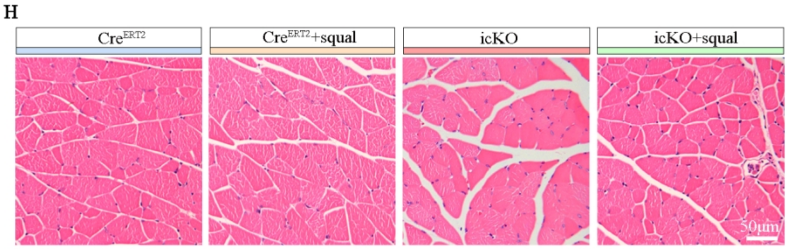

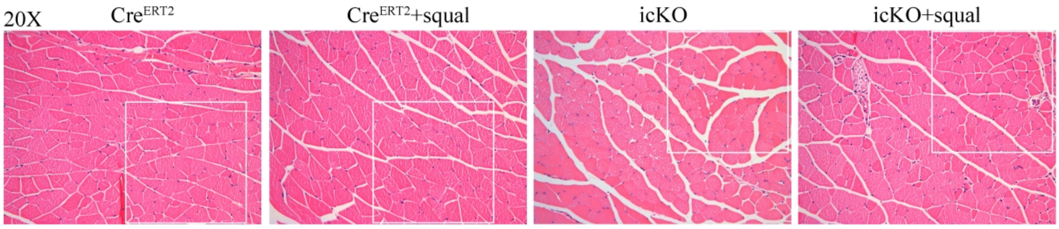

Supplement Figure 9A

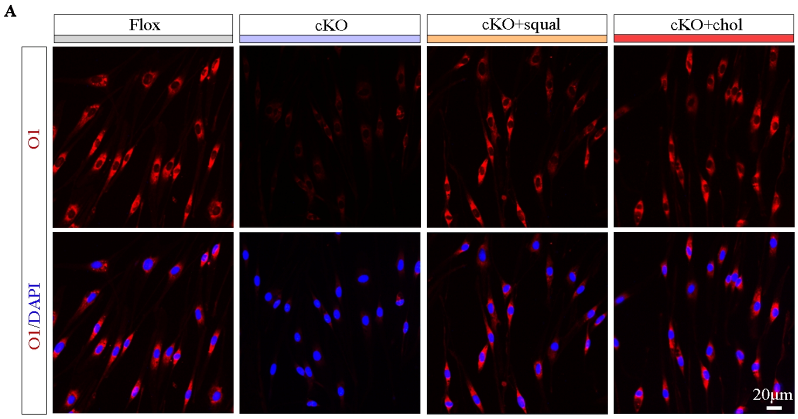

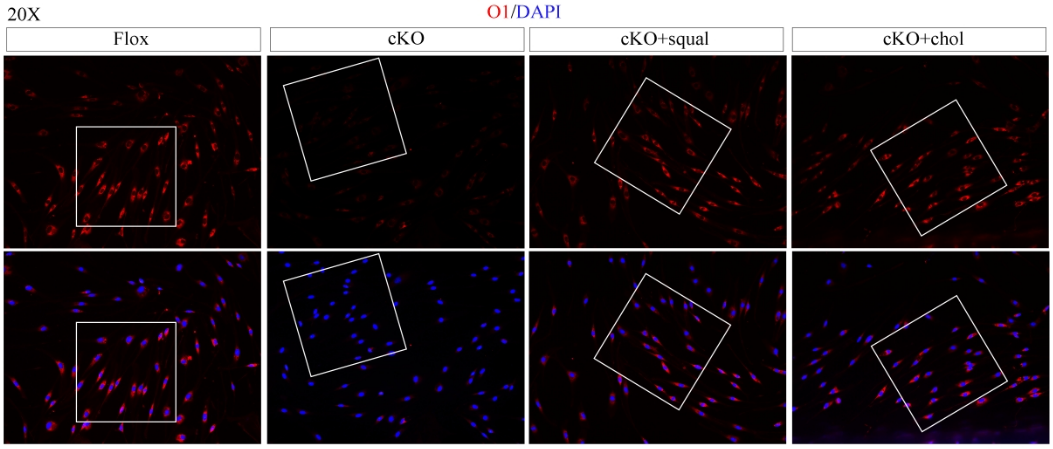

Supplement Figure 9B

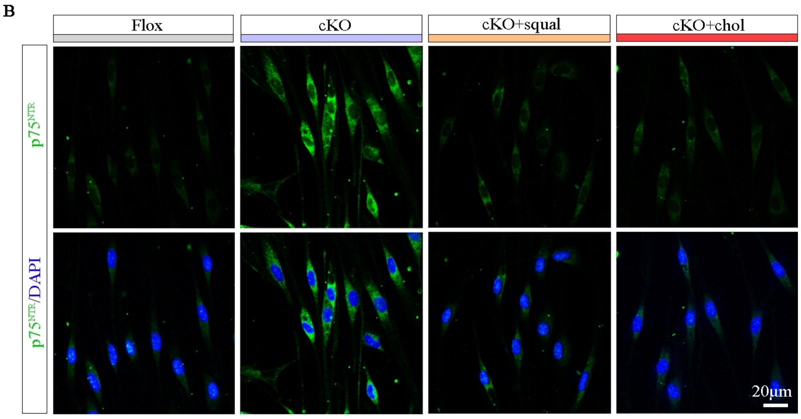

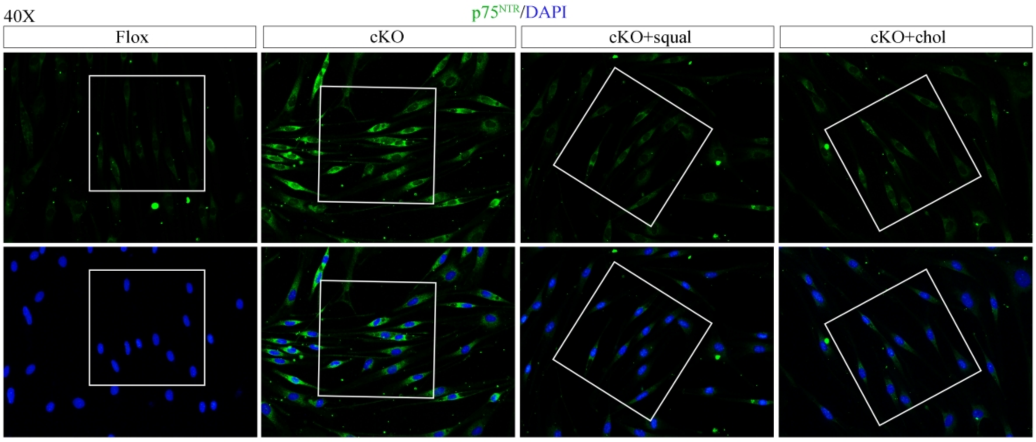

Supplement Figure 10C

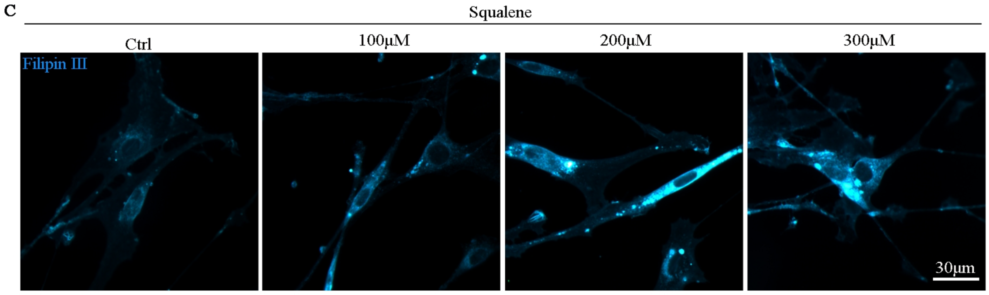

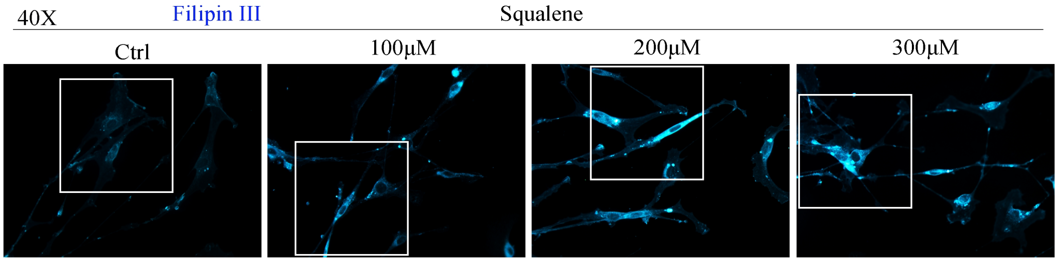

Supplement Figure 10D

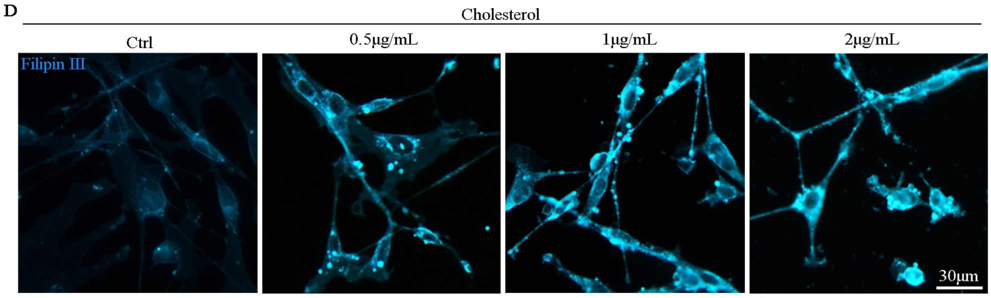

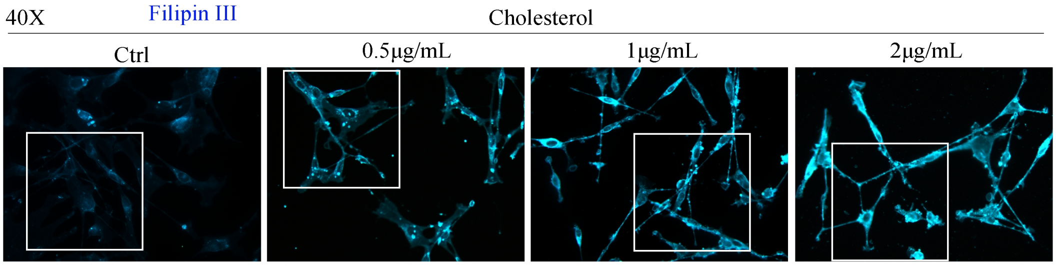

Supplement Figure 10E

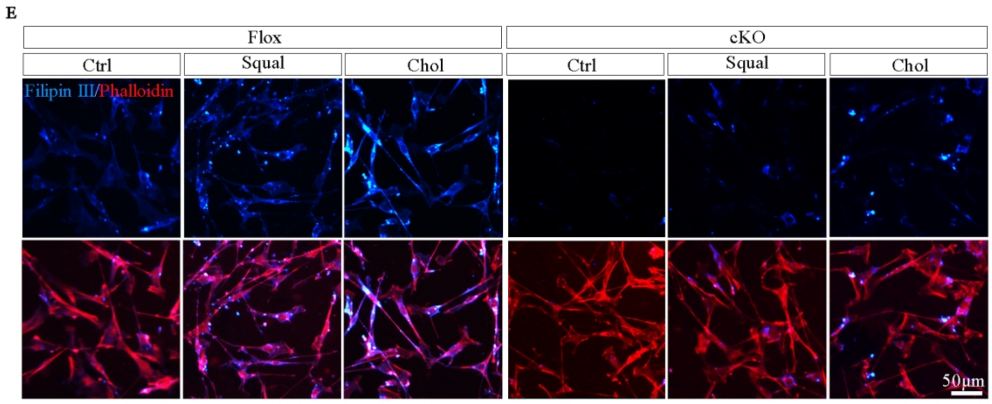

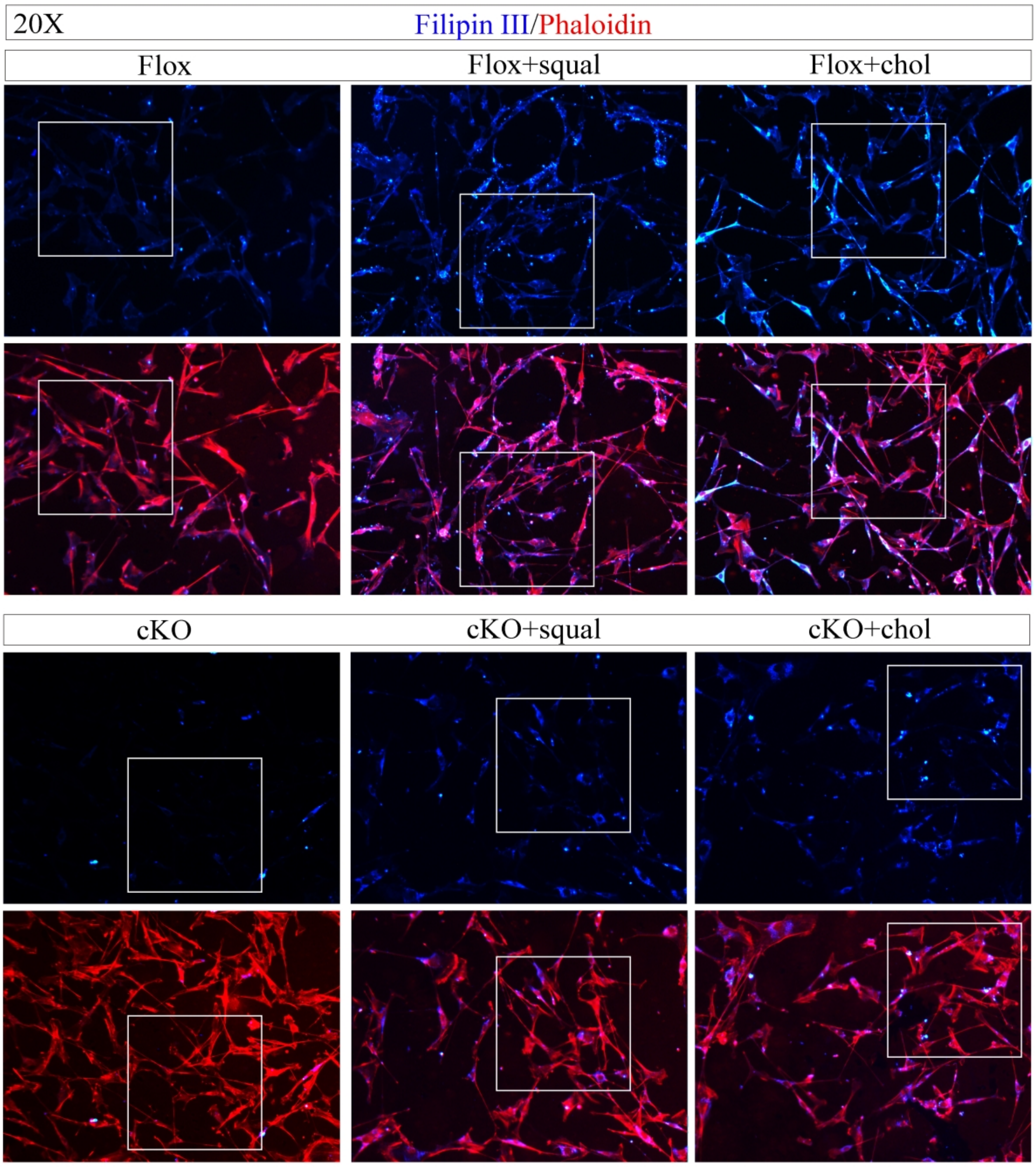

Supplement Figure 11A-C

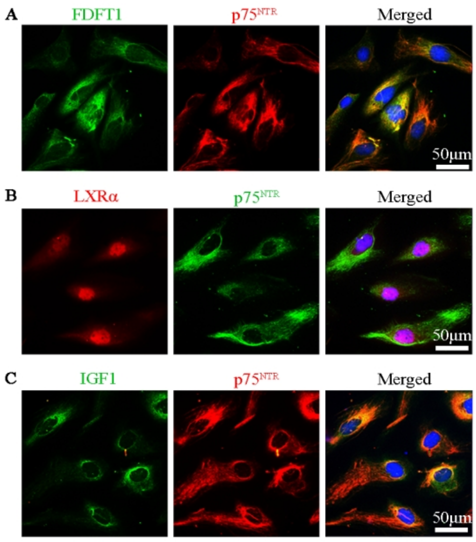

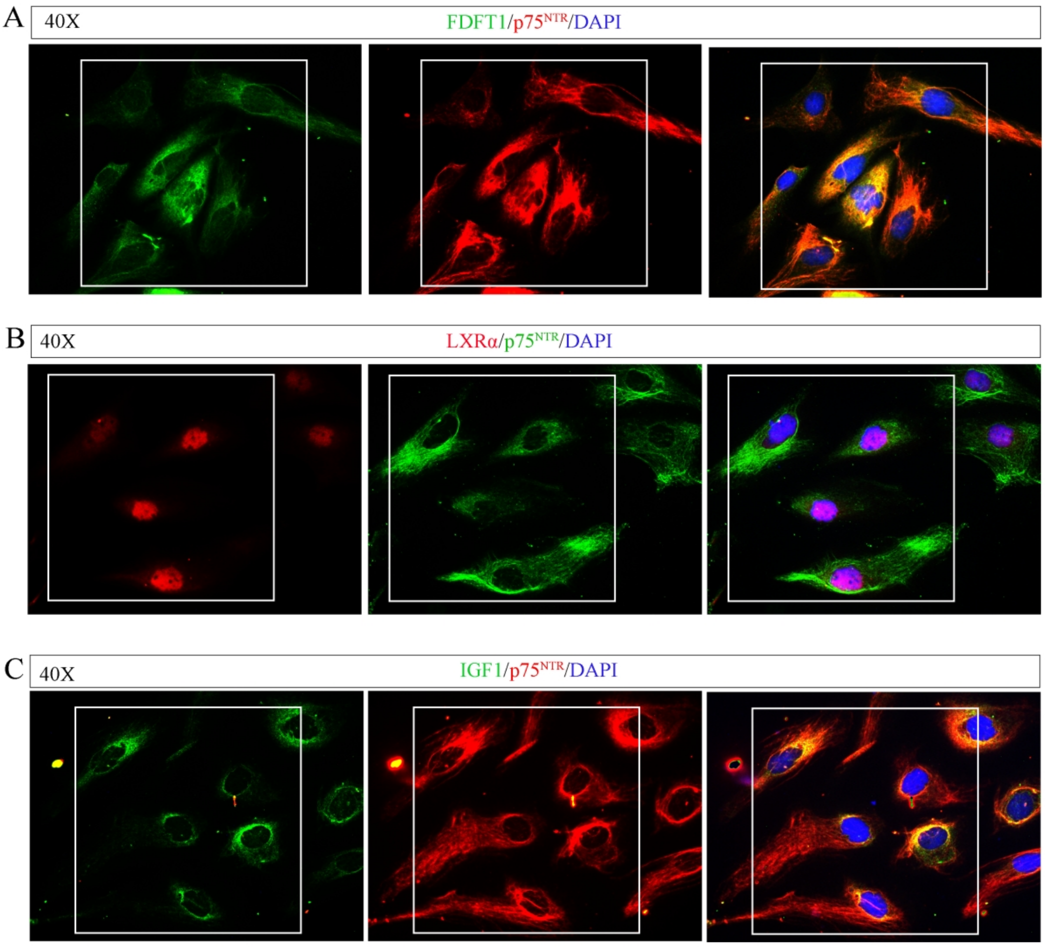

Supplement: Supplementary file 2 — Supporting File 2: advs73654‐sup‐0002‐Data.zip. [file ADVS-13-e20323-s001.zip › RAW DATA of Microscope Images.pdf]
