# Supplementary material for: Schwann Cell Synthesized Cholesterol Orchestrates Peripheral Nerve Regeneration via Structural and IGF1‐Dependent Signaling Mechanisms
Source: Adv Sci (Weinh). 2026 Jan 4;13(16):e20323. doi: 10.1002/advs.202520323 (PMC13042600; doi:10.1002/advs.202520323)
Supplement: Supplementary file 2 — Supporting File 2: advs73654‐sup‐0002‐Data.zip. [file ADVS-13-e20323-s001.zip › Cell Line Authentication STR Profiling.pdf]

Cell Line Authentication

STR Profiling

Sample information

Sample code :

Table 1. Sample code

Customer's code	Company Code
	IMMO-241365

Sample Number : 1

Sample Type : Cell line

Testing Type : STR

Sample From : Immocell Biotechnology, Xiamen, China

Testing Method:

1. Chelex 100 extraction of cell line genomic DNA.
2. Multiple PCR with SiFaSTR 23plex kit.
3. Capillary electrophoresis on ABI 3130 XL genetic analyzer.
4. Data analysis with GeneMapper® ID v3.2 software.
5. Data comparison in EXPASY database.

Test Results

1. Result

Table 2. Matching information on the cell lines

Sample Code	Multi-allele	Cell line matched	Cell Bank	EV value	Description
IMMO-241365	No	sNF96.2	EXPASY	1	Perfect match

- Multi-allele means some STR contain more than two loci.
- The results of each Genotyping in this test are good.

2. Sample Description

- **IMMO-241365 :** This cell line was identified as **the human cell line**, and the DNA of the cell lines found to **match exactly** he type of cell lines in a cell lineretrieval, EXPASY database shows that cells called **sNF96.2** corresponding to the cell number **CVCL_k281** Multiple alleles **weren't found and no cross contamination** were found in this cell line.

Note: The tested cell lines were compared with the STR reference data in ExPASy cell bank (ExPASy contains about 8808 STR data of human derived cells from ATCC, DSMZ, JCRB, ECACC, Riken and other databases, which were updated in Sep 2024). Cell lines that do not have STR data will not be able to be compared. According to the ATCC Standard Committee identification standard (ANSI/ATCC ASN-0002-2022), the matching degree EV≥80% is considered to be related, possibly derived from a common ancestor cell; The matching degree is between 55%-80%, and the correlation needs to be further identified with other methods.

3. Genotyping Result

Table 3.STR and Amelogenin Genotyping Results of Cell line IMMO-241365						
Marker	STR information of Cell			Cell Bank information		
	Sample：IMMO-241365			Cell Bank Cell name: sNF96.2		
	Allele1	Allele2	Allele3	Allele1	Allele2	Allele3
Amelogenin	X					
D3S1358	16			16		
D5S818	11			11		
D2S1338	17					
TPOX	11			11		
CSF1PO	12			12		
Penta D	14					
TH01	6			6		
vWA	17	19		17	19	
D7S820	10	11		10	11	
D21S11	30			30		
Penta E	15					
D10S1248	13					
D8S1179	13			13		
D1S1656	10					
D18S51	16			16		
D12S391	15	16				
D6S1043	12					

D19S433	13	14				
D16S539	11			11		
D13S317	10			10		
FGA	22			22		

* Although allelic data for all loci are listed in this table, the matching algorithm only compares 13 core loci.

Others

1. Genotyping Strategy and Site Distribution Attached

Table 4. Experimental Strategy and Sites

	Strategy 1	Strategy 2	Strategy 3	Strategy 4
1	D3S1358	Amelogenin	D10S1248	D19S433
2	D5S818	TH01	D8S1179	D16S539
3	D2S1338	vWA	D1S1656	D13S317
4	TPOX	D7S820	D18S51	FGA
5	CSF1PO	D21S11	D12S391	
6	Penta D	Penta E	D6S1043	

2. STR database comparison

Comparison of STR typing data was performed in the EXPASY database, which contained STR data of 8808 human cell lines from ATCC, DSMZ, JCRB, RIKEN and other databases (Cellosaurus release 50.0, Last updated Sep 2024). If the cells to be tested are not included in EXPASY cell Bank or a new cell line established by oneself, the STR typing data comparison will not be possible, and the user will need to compare the cell typing results with other databases.

Report Time: Nov 4, 2024

Applied Biosystems

GeneMapper ID v3.2

241104

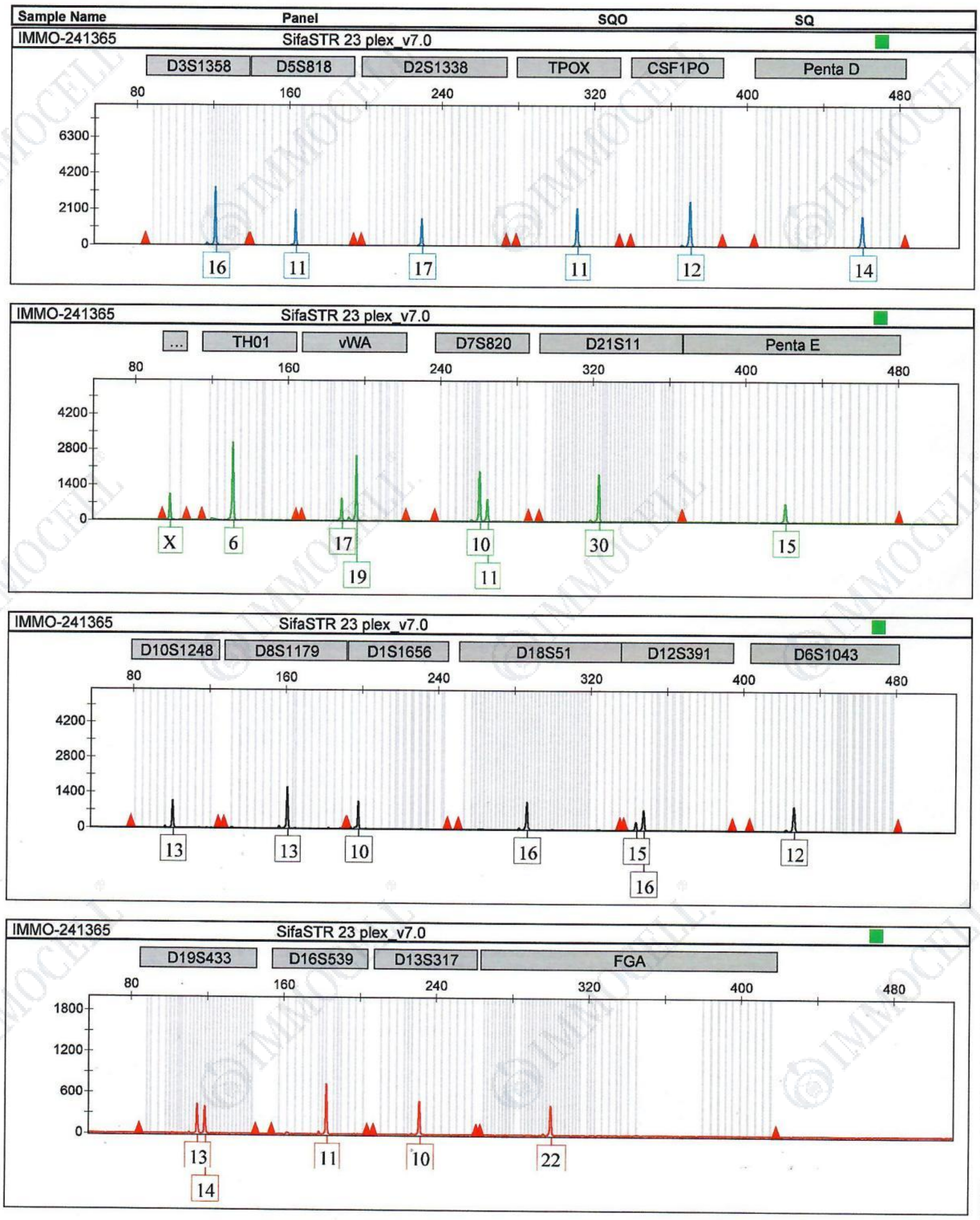

Mon Nov 04,2024 03:02PM, CST

Printed by: gmid

IMMOCELL
